# Single-cell low-pass whole genome sequencing accurately detects circulating tumor cells for liquid biopsy-based multi-cancer diagnosis

**DOI:** 10.1038/s41698-024-00520-1

**Published:** 2024-02-06

**Authors:** Xiaohan Shen, Jiao Dai, Lingchuan Guo, Zhigang Liu, Liu Yang, Dongmei Gu, Yinghong Xie, Zhuo Wang, Ziming Li, Haimiao Xu, Qihui Shi

**Affiliations:** 1 Key Laboratory of Whole-Period Monitoring and Precise Intervention of Digestive Cancer (SMHC), Minhang Hospital and Shanghai Key Laboratory of Medical Epigenetics, Institutes of Biomedical Sciences, Fudan University, Shanghai, 200032 China; 2 Department of Pathology, Zhejiang Cancer Hospital, Hangzhou Institute of Medicine (HIM), Chinese Academy of Sciences, Hangzhou, Zhejiang 310022 China; 3 Department of Pathology, The First Affiliated Hospital of Soochow University, Suzhou, 215000 China; 4 Shanghai Bone Tumor Institute and Department of Orthopedics, Shanghai General Hospital, Shanghai Jiao Tong University School of Medicine, Shanghai, 200080 China; 5 Shanghai Lung Cancer Center, Shanghai Chest Hospital, Shanghai Jiao Tong University School of Medicine, Shanghai, 200030 China; 6 Shanghai Engineering Research Center of Biomedical Analysis Reagents, Shanghai, 201203 China

**Keywords:** Next-generation sequencing, Molecular medicine

## Abstract

Accurate detection of circulating tumor cells (CTCs) in blood and non-blood body fluids enables generation of deterministic cancer diagnosis and represent a less invasive and safer liquid biopsy approach. Although genomic alternations have been widely used in circulating tumor DNA (ctDNA) analysis, studies on cell-based genomic alternations profiling for CTC detection are rare due to major technical limitations in single-cell whole genome sequencing (WGS) including low throughput, low accuracy and high cost. We report a single-cell low-pass WGS-based protocol (scMet-Seq) for sensitive and accurate CTC detection by combining a metabolic function-associated marker Hexokinase 2 (HK2) and a Tn5 transposome-based WGS method with improved cell fixation strategy. To explore the clinical use, scMet-Seq has been investigated with blood and non-blood body fluids in diagnosing metastatic diseases, including ascites-based diagnosis of malignant ascites (MA) and blood-based diagnosis of metastatic small-cell lung cancer (SCLC). ScMet-Seq shows high diagnostic sensitivity (MA: 79% in >10 cancer types; metastatic SCLC: 90%) and ~100% of diagnostic specificity and positive predictive value, superior to clinical cytology that exhibits diagnostic sensitivity of 52% in MA diagnosis and could not generate blood-based diagnosis. ScMet-Seq represents a liquid biopsy approach for deterministic cancer diagnosis in different types of cancers and body fluids.

## Introduction

Genomic alternations (e.g., somatic mutation, copy number alternation and methylation) play a vital role in the development of cancer^1–3^ and have been widely used for cancer detection and surveillance by assaying circulating tumor DNA (ctDNA) in body fluids such as blood^4,5^, urine^6^ and cerebrospinal fluid (CSF)^7^. However, tumor-derived ctDNA is present in very low concentrations compared to cell-free DNA (cfDNA) from non-tumor cells that accumulate somatic mutations due to clonal hematopoiesis^8–11^. Low abundance of ctDNA and clonal hematopoiesis generate false negative and positive results, respectively, and compromise the accuracy of ctDNA analysis^4–11^. As another primary technique of liquid biopsy, circulating tumor cells (CTCs) are intact tumor cells present in body fluids and can be individually isolated from a high background of non-tumor cells for whole genome sequencing (WGS). However, single-cell DNA sequencing methods have major limitations in low throughput, low accuracy and high cost. To this end, studies on cell-based genomic alternations profiling for accurate CTC detection and cancer diagnosis are rare^12,13^. Current methods for CTC detection are mostly based on epithelial markers (e.g. FDA-cleared CellSearch system) that cause false positive results in blood and couldn’t be used in non-blood body fluids containing numerous benign cells of epithelial origin^12–15^.

To address this challenge, we report a single-cell low-pass WGS-based method to accurately detect CTCs in blood and non-blood body fluids for establishing deterministic cancer diagnosis across many cancer types. This method, termed single-cell metabolic assay and sequencing (scMet-Seq), sensitively detects suspicious CTCs (sCTCs) with immunostaining of a metabolic function-associated marker, and determines genuine CTCs by single-cell low-pass WGS for profiling copy number alternations (CNAs). CNAs are nearly ubiquitous in solid tumors but occur sporadically in benign tissues^16–18^. If at least two sCTCs exhibit concordant CNA profiles, these cells are identified as genuine CTCs and a positive scMet-Seq test is generated for establishing deterministic cancer diagnosis. However, the main challenge of single-sCTC CNA profiling is how to maintain the genome integrity during sample processing and best amplify two copies of the genome (~6 pg of DNA) while minimizing sequence-dependent bias for achieving high CNA detection accuracy and reproducibility. To solve technical bottlenecks of single-sCTC WGS, a Tn5 transposome-based protocol with improved cell fixation strategy has been developed to significantly reduce processing time and cost with enhanced success rate.

To explore the unique clinical use of CTCs, scMet-Seq has been investigated with blood and non-blood body fluids in diagnosing metastatic diseases, including ascites-based diagnosis of malignant ascites (MA) and blood-based diagnosis of metastatic small-cell lung cancer (SCLC). Diagnosis of distant metastases has a major impact on the treatment strategy but ctDNA analysis fails to effectively discriminate cancer patients with or without metastases^8^. However, detection of malignant cells in ascites establishes a MA diagnosis that denotes cancer metastases^19,20^, and elevated CTC numbers in blood might associate with metastatic stage of SCLC that is characteristic of early metastasis and poor prognosis^21–26^. In clinic, cytology is the only diagnostic method for MA diagnosis with limited diagnostic sensitivity of 50–60%^19,20^, but could not generate blood-based diagnosis due to its low sensitivity in CTC identification by morphological analysis^12,13^. Overall, scMet-Seq establishes a CNA-based CTC definition for accurately detecting CTCs in body fluids distant from primary tumors and generating diagnosis of cancer metastases across many cancer types.

## Results

### Principle of scMet-Seq

**Fig. 1:**
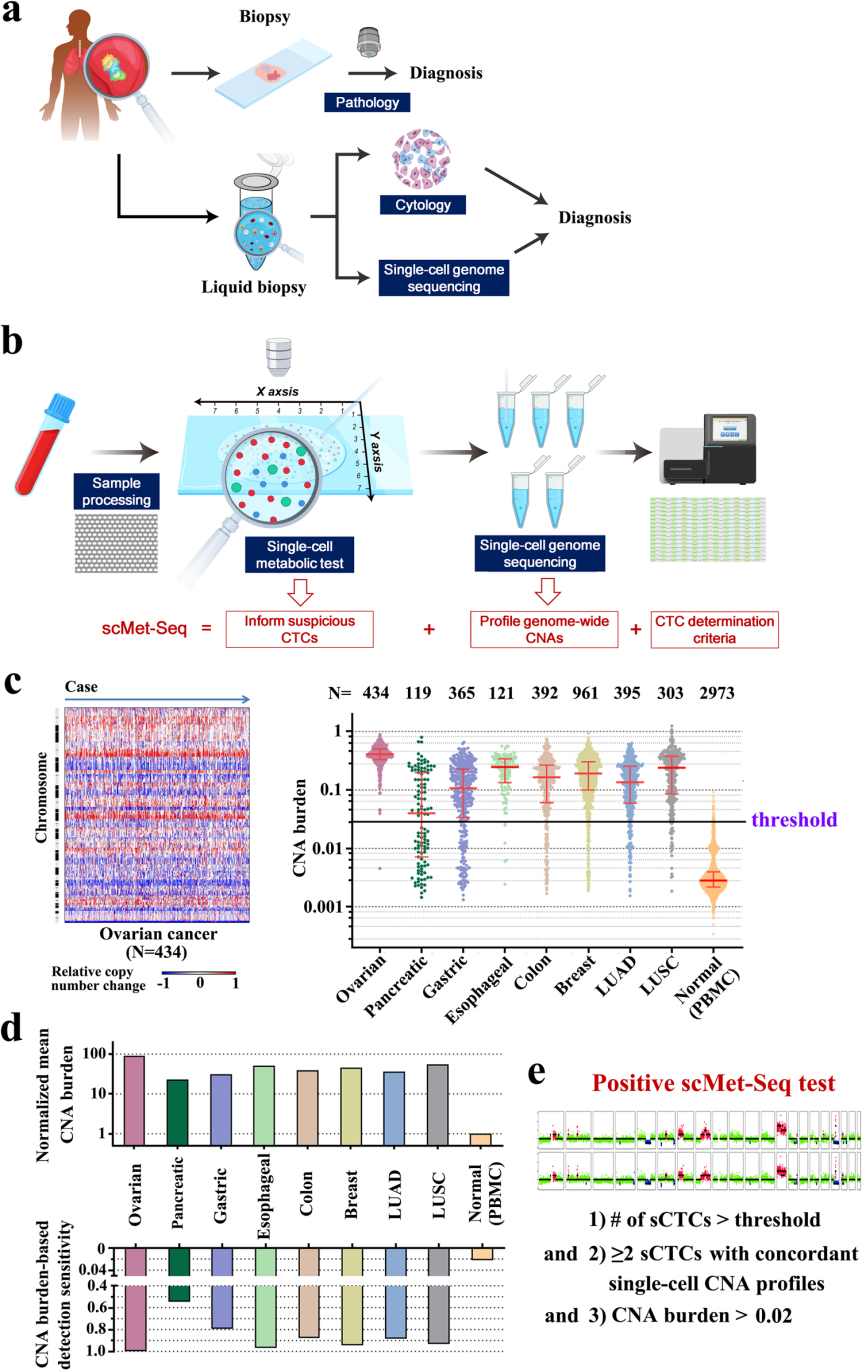
Principle of scMet-Seq. **a** Schematic illustration of biopsy- and liquid biopsy-based cancer diagnosis. **b** ScMet-Seq workflow including HK2-based immunostaining and single-cell WGS for detecting CTCs in body fluids. **c** Left, heat map of CNA profiles of ovarian cancers from Genomic Data Commons; Right, calculated CNA burden of eight types of cancers and matched normal controls (peripheral blood mononuclear cell, PBMC) from Genomic Data Commons. **d** Top, normalized mean CNA burden of eight cancer types and matched normal controls (PBMC); Bottom, CNA-based detection sensitivity at a CNA burden threshold at 0.02. **e** Definition of a positive scMet-Seq test. sCTCs: suspicious CTCs.

The scMet-Seq protocol rapidly detects sCTCs in body fluids with immunostaining of a metabolic function-associated marker, and determines genuine CTCs by single-cell low-pass WGS (mean depth: 0.2×, Fig. 1a, b). A positive scMet-Seq is defined as at least two sCTCs exhibiting concordant CNA profiles. Importantly, a CNA-based CTC definition is used in scMet-Seq instead of epithelial marker-based definition used in the past two decades, and this is essential for establishing deterministic cancer diagnosis^12,13^.

Aberrant energy metabolism is a key hallmark of many cancers and has been clinically exploited in positron emission tomography (PET) for detecting cancer metastases in vivo^27^. Critical to the aberrant metabolism, increased Hexokinase 2 (HK2) activity associated with elevated glycolysis is found in a wide range of cancers^28–30^. To this end, HK2 has the potential to be a general marker for sensitively detecting sCTCs in different types of body fluids and across many cancer types, in combination with cytokeratin (CK, epithelial marker), CD45 (leukocyte marker) and DAPI (nucleus). HK2-based immunofluorescence staining allows rapidly screening sCTCs from millions of cells in body fluids.

### CNA profile is a sensitive and highly specific molecular feature for accurately detecting CTCs

Somatic CNAs are found in a majority of solid tumors but occur sporadically in benign tissues^16–18^. Fig. 1c and Supplementary Fig. 1 show CNA burden (the percentage of the tumor autosomal genome with copy number altered) of a variety of cancers. Mean CNA burdens of these cancers vary from 23 to 90 times higher than that of normal controls (Fig. 1d). At a CNA burden threshold of 0.02, 100%, 55%, 79%, 97%, 88%, 94%, 88% and 93% of ovarian, pancreatic, esophageal, colon, breast, gastric cancers, lung adenocarcinoma (LUAD) and lung squamous cell carcinoma (LUSC) show detectable CNAs, respectively, whereas only 2.2% normal controls exhibit detectable CNAs (Fig. 1d). Since CNAs detected in non-tumor cells are mostly random and not recurrent^31,32^, two or more sCTCs exhibiting concordant CNA profiles as a criterion for CTC determination minimizes false positive CTC identification.

A positive scMet-Seq test is defined as the number of sCTCs higher than a threshold and that ≥ 2 sCTCs with detectable CNAs (CNA burden> 0.02) exhibit concordant CNA profiles (Fig. 1e). On the contrary, the number of sCTCs lower than the threshold or sCTCs absent of detectable or concordant CNAs lead to a negative scMet-Seq test. Positive scMet-Seq result enables generation of deterministic diagnosis of cancer or cancer metastasis.

### Low-cost Tn5-based protocol for single-cell WGS of CTCs with high success rate

**Fig. 2:**
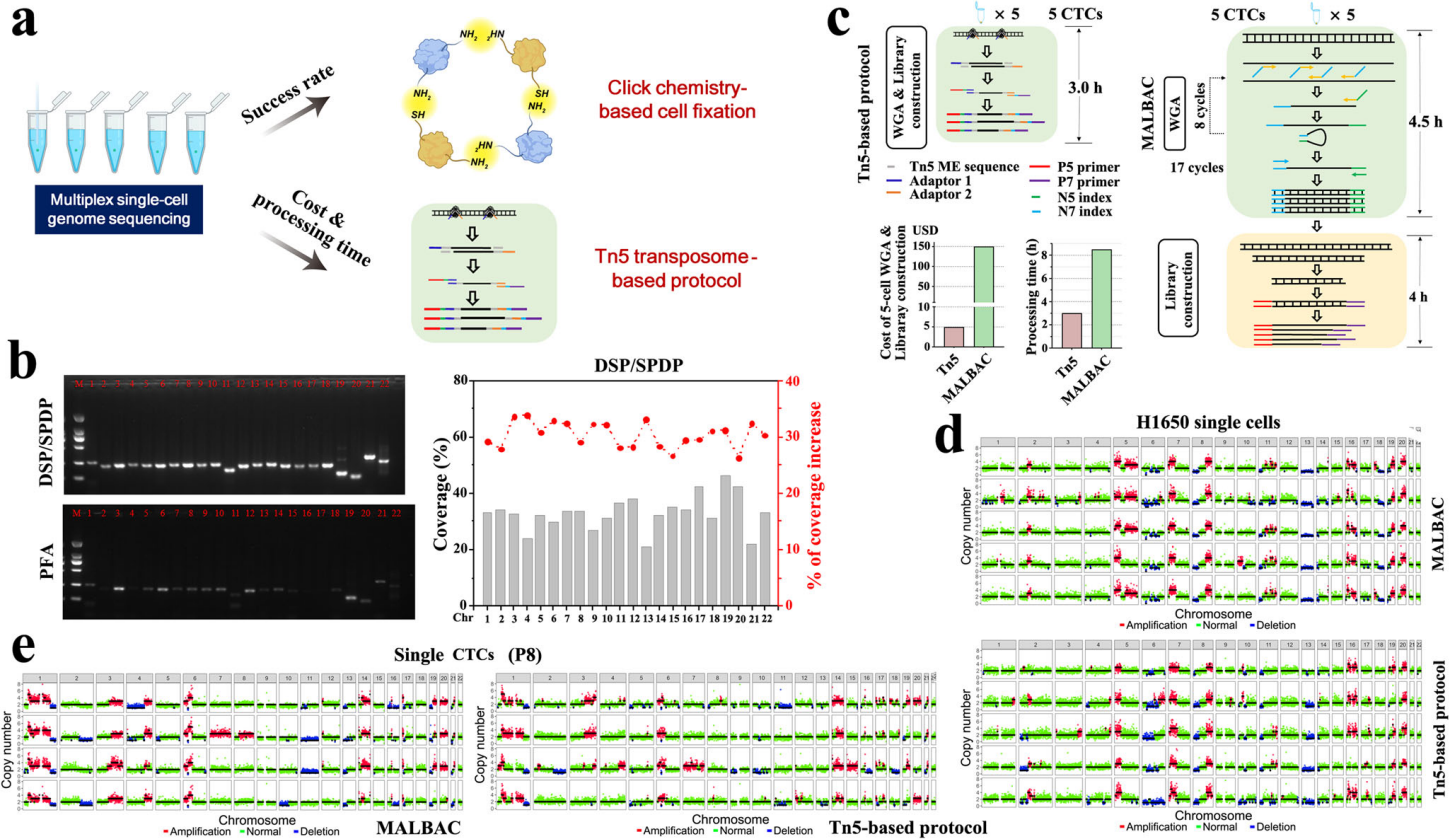
Tn5-based protocol for high efficient and low-cost single-cell WGS. **a** A click chemistry-based cell fixation method using dithiobis[succinimidylpropionate] (DSP) and N-succinimidyl 3-(2-pyridyldithio) propionate (SPDP) for increasing success rate of single-cell WGS, and a Tn5 transposome-based protocol for reducing cost and processing time. **b** Fresh RT4 cells (bladder cancer cell line) were fixed with different cell fixation methods (DSP/SPDP and 4% PFA), followed by immunostaining and stored at 4 ^o^C for 48 h. Left, single-cell WGA and PCR reaction were performed to evaluate the quality of single-cell WGS using 22 primer pairs (see Supplementary Table 1) for targeting 22 loci on different chromosomes. Right, sequencing coverage of the single cell fixed with DSP/SPDP and percentage of coverage increase compared with PFA fixation. **c** Schematic illustration of Tn5-based and MALBAC protocols, as well as comparison of cost and processing time between two protocols. **d** CNA profiles of DSP/SPDP-fixed single H1650 cells using MALBAC and Tn5-based protocols. **e** CNA profiles of DSP/SPDP-fixed single CTCs from P8 using MALBAC and Tn5-based protocols.

Different from cell lines or fresh tumor cells disassociated from tumor tissues, successful single-cell WGS of CTCs faces a daunting series of challenges including CTC enrichment, cell fixation, multicolor immunostaining, and on-slide storage at 4^o^C before CTC retrieval for sequencing. Obviously, low success rate of CTC sequencing significantly limits single-cell WGS as a means to identify CTCs. To resolve this bottleneck, we firstly developed a click chemistry-based cell fixation method to improve sequencing quality. Amine- and sulfhydryl-reactive crosslinkers are used to form amine-to-amine and amine-to-sulfhydryl crosslinks among biomolecules for cell fixation (Fig. 2a). Compared to traditional paraformaldehyde (PFA)-based cell fixation, the new cell fixation method exhibits a significant improvement in WGS of single tumor cells after immunostaining and storage steps that mimics clinical sample processing (Supplementary Table 1, Fig. 2b, Supplementary Fig. 2). At the similar sequencing depth (~2×), the new cell fixation method shows approximately 30% increase of sequencing coverage (Fig. 2b, Supplementary Fig. 3). Thus, the click chemistry-based cell fixation protects nucleic acid from degradation and keeps immunoreactivity of proteins in CTCs for immunostaining.

ScMet-Seq requires multiple single-cell WGS to determine genuine CTCs. Although low-pass WGS for genome-wide CNA profiling is inexpensive, the cost of single-cell genome amplification and library construction significantly increase overall cost. To address this challenge, we have developed a low-cost, Tn5 transposome-based single-cell WGS protocol that is modified from the Acoustic Cell Tagmentation (ACT) protocol^33^. This Tn5-based single-cell WGS protocol combines whole genome amplification (WGA) and sequencing library construction into a single step, and thereby significantly shortens time for library construction and reduces the cost (Fig. 2c). A total of 8.5 h of overall processing time in the MALBAC (multiple annealing and looping-based amplification cycles) protocol has been reduced to only 3 h. Meanwhile, the Tn5-based protocol has a 97% cost reduction compared with the MALBAC protocol. A model system of known DNA concentrations as input shows single-cell resolution of the Tn5-based protocol for CNA profiling (Supplementary Fig. 4). Importantly, the Tn5-based protocol shows genome-wide CNA profiles of H1650, H1975 single cells (lung cancer cell lines) and single CTCs from P8 comparable to those generated from the MALBAC protocol (Fig. 2d, e and Supplementary Fig. 5) but with significantly reduced processing time and cost. Overall, low-cost immunostaining and Tn5-based single-cell low-pass WGS define scMet-Seq an inexpensive diagnostic tool for clinical use.

### ScMet-Seq diagnoses MA with high diagnostic accuracy

MA refers to a fluid containing tumor cells in the abdomen and manifests end stage events with a poor prognosis (median survival: 5.6 months) because > 95% of MA originates from cancer metastases^19,20^. Many cancers are likely to cause MA and it is the initial presenting sign or symptom of malignancy in ~50% of cases^19,20^. Hence, diagnosis of MA denotes cancer metastases and is technically challenging because MA is involved with a variety of cancer types. To date, cytology remains the only diagnostic method with diagnostic sensitivity of only 50–60%^19,20^. A number of small-cohort studies have investigated the utility of ctDNA in ascites for identifying targetable alternations rather than generating diagnosis^8^. We performed a clinical study to assess the performance of scMet-Seq as a multi-cancer diagnostic method for MA diagnosis.

**Fig. 3:**
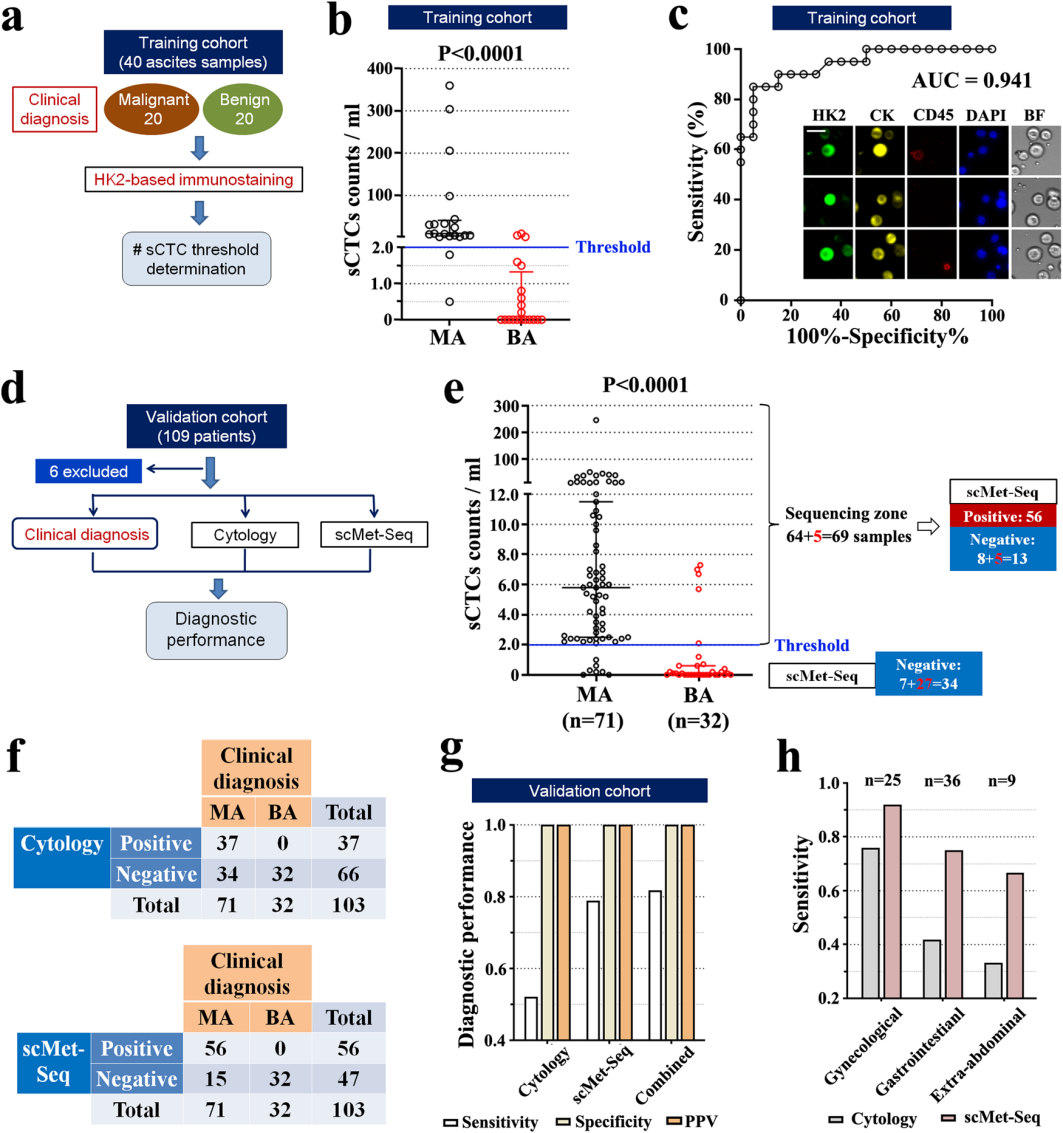
Diagnostic performance of scMet-Seq in diagnosing MA. **a** Flowchart of the training cohort establishment for determining the sCTC count threshold. **b** HK2-derived sCTC counts in clinically diagnosed MA (*n* = 20) and BA (*n* = 20) groups from the training cohort. **c** ROC curve generated from sCTC counts in the training cohort. Inset: representative fluorescence images of sCTCs. **d** Design of the validation cohort and results of cytology and scMet-Seq for assessing diagnostic performance. **e** HK2-derived sCTC counts in clinically diagnosed MA (*n* = 71) and BA groups (*n* = 32) from the validation cohort with median and interquartile range (IQR). The sCTC counts lower than the threshold generated negative scMet-Seq. Multiple sCTCs were retrieved for single-cell WGS if the sCTC counts greater than the threshold. **f**, **g** Diagnostic performance of cytology, scMet-Seq and a combination of these methods. **h** Diagnostic sensitivities of cytology and scMet-Seq in the subgroups of MA samples. sCTCs suspicious CTCs, BF bright field.

We firstly determined the sCTC counts threshold of scMet-Seq by enrolling 20 clinically diagnosed MA (including > 10 cancer types, Supplementary Table 2) and 20 benign ascites (BA, Supplementary Table 3) patients as a training cohort (Fig. 3a). Sample processing, HK2-based immunostaining and sCTC (HK2^high^/CK^+^/CD45^−^/DAPI^+^) identification were conducted according to the protocol described in the Methods and Supplementary Fig. 6. The sCTC counts per ml were significantly higher in MA samples compared with the BA group [9.6 (4.3–41.4) *vs*. 0.0 (0.0–1.3) sCTCs/ml; *P* < 0.0001] (Fig. 3b). The area under curve (AUC) of the receiver operating characteristic (ROC) curve, a global measure of the accuracy of a quantitative diagnostic test, was computed to be 0.941 (Fig. 3c). High AUC indicates excellent discrimination power of a test at discriminating malignant and benign samples. At a threshold of ≥2.0 sCTCs/ml, the diagnostic sensitivity and specificity was 0.90 and 0.85 in the training set, respectively. Obviously, subsequent single-cell WGS could eliminates false positive sCTCs and dramatically increase diagnostic specificity. Thus, we define a threshold of sCTC count ≥ 2.0/ml in ascites for conducting single-cell WGS. Meanwhile, sequencing 67 CK^+^ normal cells with epithelial origin from 14 patients in BA cohort generates a CNA burden threshold of normal cells at 0.02 (Supplementary Fig. 7) that equals the CNA burden threshold determined from tissue samples (Fig. 1c). Thus, a cell with detectable CNAs is defined as its CNA burden greater than 0.02.

To investigate the diagnostic performance of scMet-Seq, a validation cohort of 109 consecutive patients with ascites were prospectively enrolled with exclusion of 6 patients due to unobtainable diagnosis or sequencing results (Fig. 3d). All samples were prospectively collected and the clinical information was blinded to the operators. The measurement of this cohort generated 56 positive (#sCTC≥2.0/ml & ≥2 sCTCs showing consistent CNA profiles & CNA burden> 0.02) and 47 negative results of scMet-Seq (Fig. 3e, Supplementary Fig. 8). The MA and BA diagnoses were then clinically established in 71 subjects comprising >10 cancer types and 32 subjects (Table 1, Supplementary Tables 4, 5), respectively. ScMet-Seq showed diagnostic sensitivity, diagnostic specificity, positive predictive value (PPV), and negative predictive value (NPV) of 79%, 100%, 100%, and 68%, respectively (Fig. 3f, g, Supplementary Table 6). By contrast, ascites cytology exhibited diagnostic sensitivity of 52% in MA diagnosis. A combination of scMet-Seq and cytology achieved a 82% of diagnostic sensitivity, 100% of diagnostic specificity and PPV (Fig. 3g, Supplementary Table 6). Overall, scMet-Seq enables MA diagnosis across >10 cancer types with 100% of PPV and diagnostic specificity, and superior diagnostic sensitivity over cytology, especially in gastrointestianl and extra-abdominal cancers (Fig. 3h).

**Table 1:** Clinicopathological characteristics and sCTC counts of participants in the validation cohort of ascites study.

Patient variable	MA	BA	*p*-value
*N*	71	32	
Female (%)	73.2%	62.5%	0.27^*^
Age, median (IQR)	61 (54.5–68)	62.5 (52–72)	0.79
Cancer type			
1) Gynecological cancers (*n* = 25)			
Ovarian cancer	21		
Cervical cancer	2		
Endometrial cancer	2		
2) Gastrointestianl cancers (*n* = 36)			
Colorectal & small intestine cancer	9		
Gastric cancer	12		
Esophageal cancer	2		
Pancreatic cancer	5		
Hepatocellular carcinoma	4		
Cholangiocarcinoma	3		
Gallbaldder cancer	1		
3) Extra-abdominal tumors (*n* = 9)			
Breast cancer	2		
Lung cancer	1		
Nasopharyngeal cancer	1		
Lymphoma	2		
Sarcoma	1		
Cancers of unknown primary	2		
4) Primary peritoneal mesothelioma	1		
sCTC count/ml, median (IQR)	5.8 (2.5, 11.5)	0.1 (0, 0.6)	< 0.0001

*MA* Malignant ascites, *BA* Benign ascites, *IQR* Interquartile range. ^*^Pearson chi-square test was used for comparison.

### CTC-derived CNA profiles concordant with those of tumor tissues

**Fig. 4:**
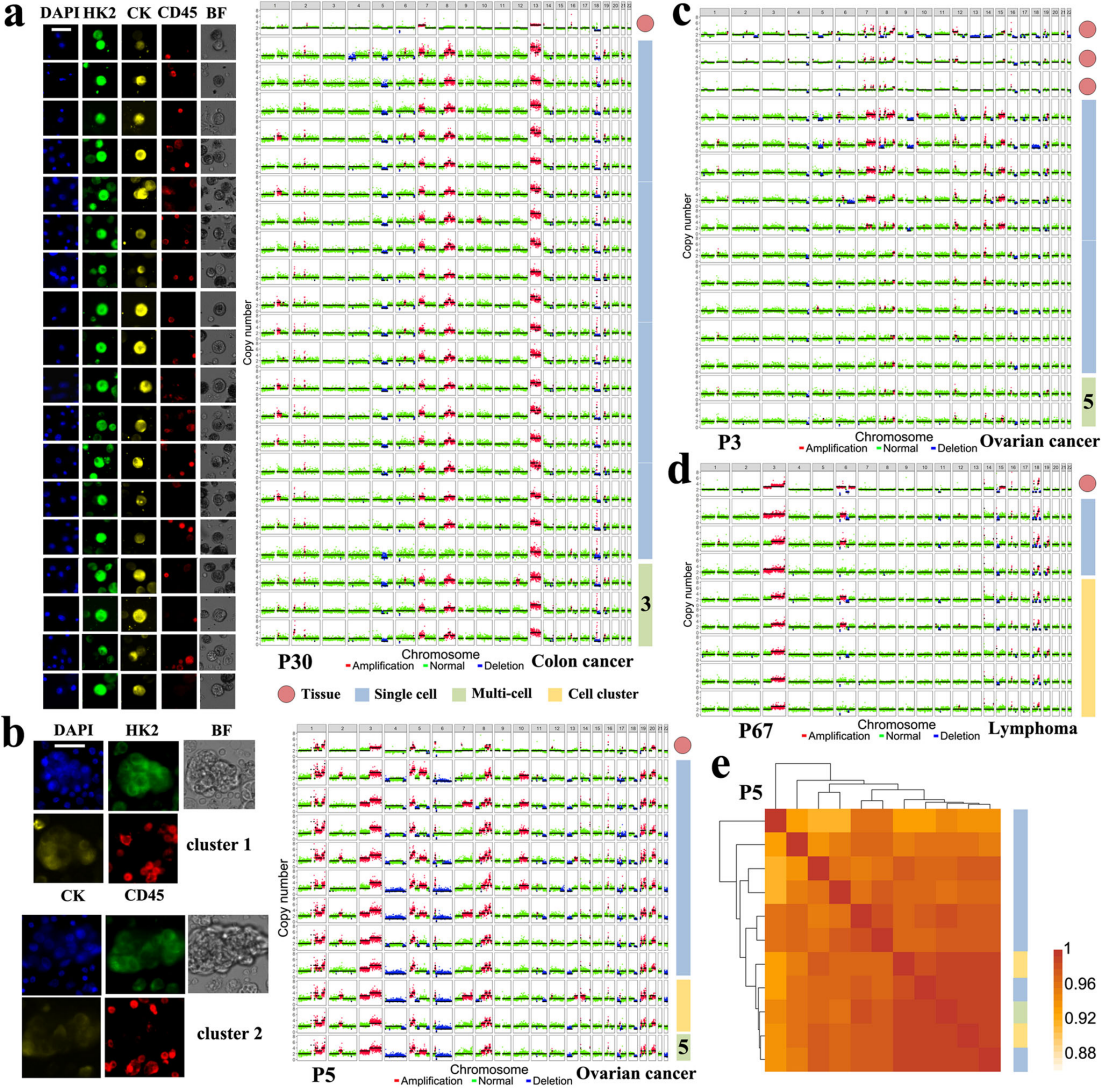
Single-cell low-pass WGS accurately detects CTCs for definitive MA diagnosis. **a** Left, representative fluorescence images of HK2-derived sCTCs from P30; Right, single-cell CNA profiles of 19 single CTCs, 3 three-CTC pools, as well as the bulk tumor tissue from primary site. **b** Left, representative images of sCTC clusters from P5; Right, single-cell CNA profiles of 9 single CTCs, 2 CTC clusters, one 5-CTC pool, as well as the bulk tumor tissue from primary site. **c**, **d** Single-cell CNA profiles of single CTCs, CTC clusters, mutli-CTC pools, as well as bulk tumor tissues from P3 and P67, respectively. The ascites cytology of P67 showed an atypical result. **e** Heat map of correlation coefficients of CNA profiles between single CTCs, multi-CTC pools and CTC clusters from P5. All correlation coefficients in this matrix are higher than 0.91. Scale bar: 50 μm.

High diagnostic specificity and PPV of scMet-Seq arises from the CTC determination criterion that requires consistent CNA profiles among multiple cells. As shown in Fig. 4a–d, reproducible gains and losses in CNA patterns were found in single CTCs, multi-CTC pools (3 ~ 5 cells) and CTC clusters (5 ~ 10 cells, Supplementary Figs. 9, 10). High correlation coefficients between CNA profiles (Fig. 4e, Supplementary Fig. 11) indicates high-quality of single-cell WGS and clonal expansion that is characteristic of malignant cells. Importantly, CTC-derived CNA profiles are found concordant with those of tumor tissues (Fig. 4a–d), and this provides compelling evidence of tumor origin of CTCs. It is noteworthy that only a fraction of cfDNA-derived genomic alternations can be identified in tumor tissues, leading to development of tumor-informed ctDNA analysis. In addition, no significant difference in CNA profiles were found between single CTCs and multiple CTCs (multi-CTC pools and CTC clusters), demonstrating high quality of genome-wide CNA profiling in single CTCs with the scMet-Seq protocol. Since CTC clusters are rare in body fluids for most cancers, ~5 single sCTCs are usually collected for performing single-cell low-pass WGS.

In the scenario of liquid biopsy, tumor tissues are unavailable for sequencing as the reference. Concordant CNA profiles in ≥2 sCTCs are used to identify genuine CTCs for establishing deterministic cancer diagnosis (Supplementary Figs. 12–48). Since benign cells are absent of detectable or concordant CNA profiles^31^, single-cell WGS eliminates false positive sCTCs and achieves ~100% of diagnostic specificity and PPV. However, a small fraction of tumors are found absent of detectable CNAs (Supplementary Fig. 32), leading to false negative of scMet-Seq and lowering its diagnostic sensitivity.

### Blood-based diagnosis of metastatic SCLC

SCLC represents ~15% of all lung cancer and is characterized by rapid growth, early metastasis and poor prognosis^21,26^. Approximately 60-70% of patients have metastatic disease at diagnosis^21^. Diagnosis of distant metastases has major implications for management and prognosis. Peripheral blood as a window of blood-borne tumor metastasis has the potential to be a non-invasive means for diagnosing metastatic SCLC. To date, the CellSearch^®^ System is the only FDA-cleared system for detecting and enumerating CTCs of epithelial origin in blood using a combination of epithelial markers (EpCAM and CK) and leukocyte marker (CD45). CellSearch and CellSearch-like methods have reported 60 ~ 70% and 0 ~ 20% of CTC-positive rates (CTC counts≧ 2/ml) in the extensive disease (ED) and the limited disease (LD) stages of SCLC, respectively^22–26^. However, these studies fail to determine cell malignancy of CTCs detected with epithelial markers and haven’t investigated the utility of CTCs in diagnosing metastatic SCLC.

**Fig. 5:**
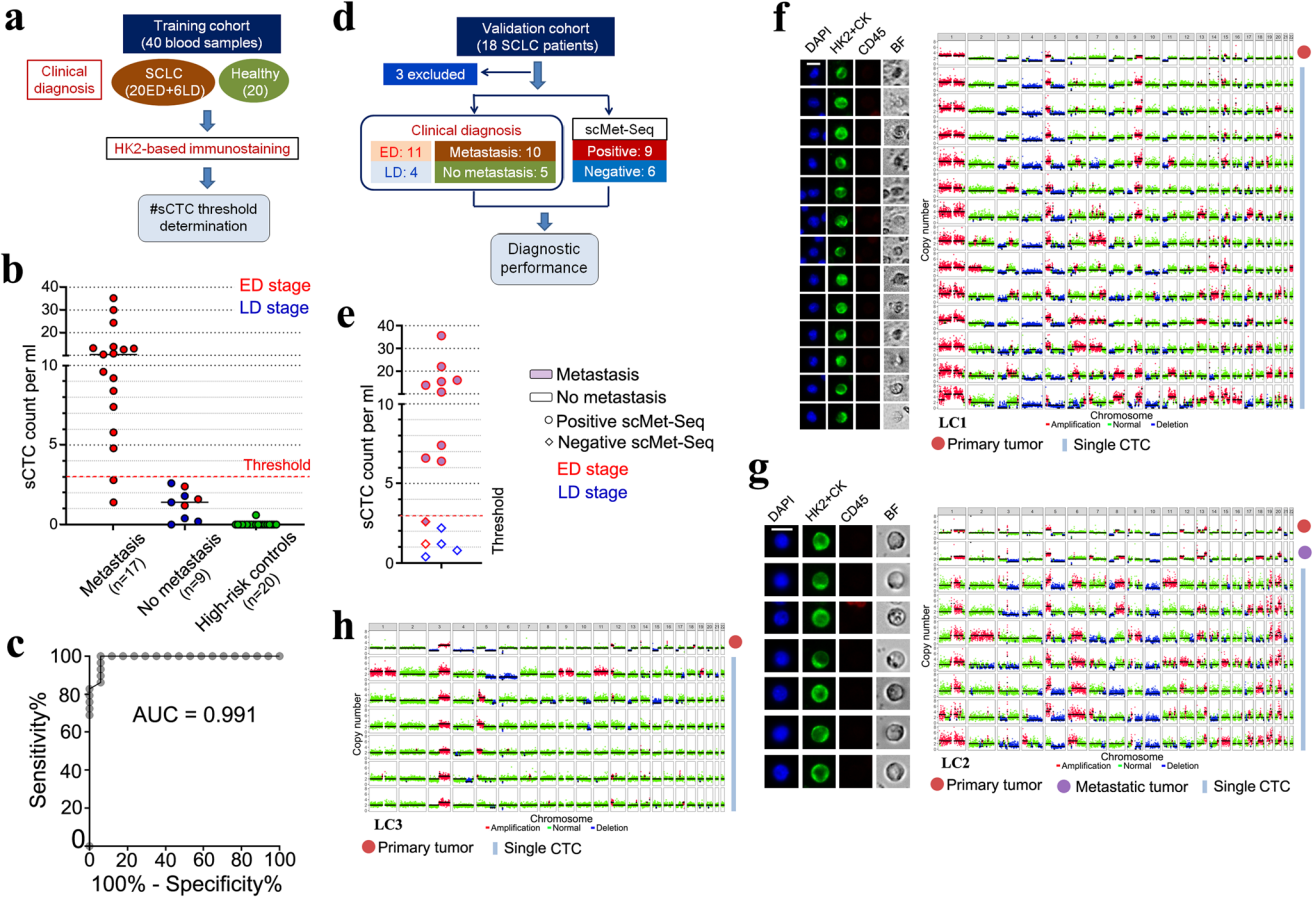
Blood-based diagnosis of metastatic SCLC via scMet-Seq. **a** Flowchart of the training cohort establishment for determining the sCTC count threshold for diagnosing metastatic SCLC. ED: extensive disease; LD: limited disease. **b** sCTC counts (HK2+CK^high^/CD45^−^/DAPI^+^) of SCLC patients (*n* = 26) and high-risk controls (*n* = 20). **c** ROC curve generated from sCTC counts in the training cohort including individuals with (*n* = 17) and without metastases (*n* = 29). **d** The validation cohort for assessing diagnostic accuracy of scMet-Seq in diagnosing metastatic SCLC. **e** scMet-Seq results of the validation cohort measurement. **f**, **g** Representative fluorescence images of CTCs and single-cell CNA profiles, along with the CNA profiles of bulk tumor tissues from patient LC1 and LC2. Scale bar: 10 μm. (**H**) CNA profiles of bulk tumor tissue and single CTCs from patient LC3.

We firstly generated a training cohort to determine the sCTC count threshold of diagnosing metastatic SCLC, including 26 treatment naïve SCLC patients (20 ED and 6 LD) and 20 high-risk controls (Fig. 5a, Supplementary Table 7). To increase detection sensitivity, we combined the metabolic (HK2) and epithelial (CK) markers in a single fluorescence color (Supplementary Fig. 49) because our previous study identified a HK2^+^/CK^−^ CTC subset in LUAD with identical CNA patterns of CK^+^ CTC population^17^. Approximately 88% (15/17) of SCLC patients with distant metastasis showed CTC counts ≧ 3.0/ml (median: 10.4/ml; IQR: 6.6–13.5/ml) while all patients without distant metastasis and high-risk controls exhibited CTC counts < 3.0/ml (Fig. 5b). Thus, a sCTC count threshold at 3.0/ml enabled discrimination of metastatic SCLC patients from non-metastatic SCLC patients, and the AUC of ROC curve was computed to be 0.991 (Fig. 5c).

We then performed a preliminary study by measuring a validation cohort of SCLC patients (N = 18) with exclusion of 3 patients due to unobtainable sequencing results (Fig. 5d, Supplementary Table 8). In metastatic patients, 90% (9/10) of them were found positive of scMet-Seq, and all patients without distant metastasis showed negative scMet-Seq results (Fig. 5e). CTCs exhibited identical CNA patterns compared with bulk tumor tissues (Fig. 5f–h, Supplementary Figs. 50–53), indicating tumor origin of CTCs and that peripheral blood as a liquid biopsy was equivalent to tumor biopsies. Overall, single-cell WGS determines cell malignancy of biomarker-derived sCTCs for establishing deterministic malignancy diagnosis, and elevated CTC counts are associated with metastatic stage of SCLC. ScMet-Seq enables blood-based diagnosis of metastatic SCLC at a diagnostic sensitivity, specificity and of 90%, 100% and 100%, respectively.

## Discussion

Biopsy collects tissue samples for microscopic examination by a pathologist. This is the gold standard for cancer diagnosis but is an invasive procedure with concerns of feasibility and safety, and requires high degree of expertise. Liquid biopsy represents a less invasive and safer alternative to biopsy, and is broadly thought of as collection of a body fluid sample to detect tumor-derived CTCs or ctDNA. Similar to ctDNA^8^, we use term CTC to refer more broadly to tumor cells present in blood and non-blood body fluids, especially those distant from primary tumors and associated with cancer metastasis (e.g., CSF, pleural effusion, ascites, peripheral blood). Since ctDNA is present with predominant non-tumor cfDNA in biological fluids, tremendous research has been devoted to increasing detection sensitivity and identifying tumor-specific genomic alternations for reducing false positives derived from non-tumor cfDNA^4–11^. By contrast, CTCs as intact tumor cells can be individually isolated for sequencing and this provides 100% of tumor content that is equivalent to tumor tissues. This study presents scMet-Seq as a HK2-informed, single-cell low-pass WGS protocol to accurately detect CTCs for deterministic cancer diagnosis in different types of body fluids and cancers.

ScMet-Seq has three major advantages over other liquid biopsy methods. First, scMet-Seq provides deterministic cancer diagnosis with nearly 100% of diagnostic specificity and PPV. Consistent single-cell CNA profiles among multiple cells demonstrate high technical accuracy of scMet-Seq and are biologically characteristic of malignant cells. CTC-derived CNA profiles are found consistent with those of tumor tissues, indicating tumor origin of CTCs. Thus, this criterion of CTC determination identifies genuine CTCs and generates deterministic cancer diagnosis. Clinical studies in ascites have shown 100% of diagnostic specificity and PPV of scMet-Seq, and improved diagnostic sensitivity that is superior to cytology. Second, scMet-Seq is a multi-cancer diagnostic method rather than specific for a specific cancer type. Somatic CNAs and increased HK2 activity are both found in a majority of solid tumors. For this reason, HK2-informed single-cell CNA profiling for CTC detection has high sensitivity in different kinds of body fluids and across many cancer types. In this study, we detected CTCs in ascites and blood across >10 cancer types, indicating wide applicability of scMet-Seq. Third, scMet-Seq is capable of diagnosing cancer metastasis by detecting CTCs in body fluids distant from primary tumors. However, ctDNA analysis fails to discriminate metastatic and localized diseases.

Although detection of targetable driver mutations hasn’t been included in the scMet-Seq^34^, CNA profile characterization have shown prognostic value because significant associations between increasing CNA burden and more severe phenotypes or reduced survival are reported^35,36^. ScMet-Seq could be a robust diagnostic tool for malignancy diagnosis and prognosis using liquid biopsies, as well as companion diagnostics if combined with detection of targetable driver mutations.

ScMet-Seq sensitively detects sCTCs in blood and non-blood body fluids and determines genuine CTCs with rapid and low-cost single-cell CNA profiling. Since CTCs are rare in body fluids, a complicated procedure involving CTC enrichment, cell fixation, immunostaining and storage is required to detect sCTCs, leading to DNA degradation and compromised sequencing quality. Although single-cell CNA profiling of CTCs were reported in previous studies^14,30,37–39^, only a small fraction of CTCs were sequenced due to low success rate and high cost of single-cell low-pass WGS. However, the definition of scMet-Seq requires high success rate and low cost of single-cell CNA profiling to generate deterministic cancer diagnosis. To address this technical challenge, we firstly developed a click chemistry-based cell fixation approach to minimize DNA degradation and achieved ~30% increase of sequencing coverage compared with traditional PFA-based cell fixation method. Second, a Tn5-based single-cell WGS method was developed in this study by combining single-cell WGA and sequencing library construction into a single step for significant reduction of processing time and cost compared to the MALBAC protocol. MALBAC, a commercial single-cell WGA protocol, is used in this study as the comparator to the Tn5-based method because MALBAC enables detection of single-cell CNA profiles in both fresh and fixed cells with high CNA detection accuracy and uniformity^40,41^. In this study, only four and three patients in the validation ascites (109 patients) and SCLC (18 patients) cohorts were excluded due to unsuccessful single-cell WGS of sCTCs, respectively, demonstrating high success rate of scMet-Seq. This high success rate paves the way of scMet-Seq for routine clinical use.

Genome instability is a characteristic of most cancers and CNAs are major genome aberrations found in nearly all cancer cells but occur sporadically in benign tissues^16–18^. Although low-frequency somatic CNAs are found in a small fraction of normal cells, these CNAs are randomly distributed in the genome and inconsistent between different cells^31^. Thus, the criterion of two or more sCTCs exhibiting concordant CNA profiles enables identification of false positive sCTCs that could be attributed to low-frequency random CNAs or amplification bias during single-cell WGS. Meanwhile, multiple cells with concordant CNA profiles indicates clonal expansion that is a characteristic of cell malignancy. To save time and cost on single-cell CTC manipulation and CNA profiling, we have established a criterion that at least two sCTCs exhibiting concordant CNA profiles generates positive scMet-Seq. In the study, we have sequenced more sCTCs in a fraction of sample for validating our hypothesis that CTCs present in blood and non-blood body fluids harbor consistent CNA profiles across the genome in a variety of cancer types.

A notable limitation of scMet-Seq is that a small fraction of tumors are absent of detectable CNAs, and this reduces diagnostic sensitivity of scMet-Seq (Fig. 1c). Second, clinical utility of scMet-Seq is still preliminary as the cohort size is small, thereby warranting a large-scale prospective clinical trial. Third, CNA profiling in scMet-Seq is unable to trace the tissue origin of CTCs and identify the cancer type, but clinical imaging techniques usually provide clues for tissue origin of CTCs. Although scMet-Seq is a diagnostic method with wide applicability in different types of body fluids and cancers, the threshold of sCTCs has to be determined individually based on a small sized training cohort for achieving the best diagnostic accuracy. In addition, HK2 contributes to increased glucose consumption and thereby is relatively insensitive for diagnosing tumors with low glycolytic activity^42,43^. Meanwhile, there are a fraction of CTCs with low glycolytic activity and HK2 levels in body fluids due to cell death, quiescence and diverse metabolic dependencies^30^, escaping from detection with scMet-Seq.

In conclusion, we report a single-cell, low-pass WGS-based method (scMet-Seq) for accurately detecting CTCs in body fluids and establishing deterministic cancer diagnosis in many cancer types. The scMet-Seq protocol utilizes a CNA-based CTC definition that is superior to traditional epithelial marker-based CTC definition because it accurately determines cell malignancy and minimizes false positive results of CTC detection. A Tn5-based protocol with improved cell fixation method has been developed to enhance success rate of single-CTC WGS with significant reduction of cost and processing time. The CNA-based CTC definition demonstrates nearly 100% of diagnostic specificity and PPV in clinical studies. ScMet-Seq shows diagnostic sensitivity of 79% and 90% in diagnosing MA and metastatic SCLC, respectively, representing a major diagnostic improvement over current methods. Overall, scMet-Seq unlocks an innovative low-cost multi-cancer diagnostic method for liquid biopsy that complements traditional biopsy-based cancer diagnosis.

## Methods

### Study design and patient enrollment

The goal of this study was to explore whether single-cell low-pass WGS might accurately detect CTCs and establish deterministic cancer diagnosis. We conducted an ascites study at Zhejiang Cancer Hospital between March 2022 to November 2022. The study was performed according to the principles of the Helsinki Declaration and was approved by the institutional review board (#IRB2022145). A total of 149 patients (≥18 years) with ascites were enrolled in this study with written informed consent. All clinical diagnoses of ascites were based on the following criteria. A malignant ascites diagnosis includes: 1) positive cytology; or 2) positive peritoneal biopsy; or 3) hispathological confirmation of primary tumor and the patient had clinical or radiographic evidence of metastatic disease and had no alternative cause for the ascites. A benign ascites diagnosis is defined as: 1) the patient had no evidence of malignancy; and 2) a strong etiology of benign disease could explain the ascites. Patients with unobtainable diagnosis or sequencing results (all sequenced sCTCs fail to pass the quality control procedure) were excluded in the analysis. The SCLC study was conducted at Shanghai Chest Hospital between March 2022 to December 2022 in compliance with the principles of the Helsinki Declaration and approved protocol (#IS21109). A total of 44 SCLC patients and 20 high-risk controls (8 subjects with benign nodules and 12 current smokers) were enrolled in the study with written informed consent. The study is compliant with the ‘Guidance of the Ministry of Science and Technology (MOST) for the Review and Approval of Human Genetic Resources’.

### Sample collection and the brief scMet-Seq protocol

For each patient with ascites, 10 ml of ascites was collected and sent to the lab for scMet-Seq at 4 ^o^C within 4 hours, and an ascites sample (50 ml) was also sent for cytologic examination at the same time. For each SCLC patient and high-risk individual, 5 ml of peripheral blood was collected and sent to the lab at 4 ^o^C within 4 h. All samples were anonymously coded and technicians were blinded to the clinical information. Received ascites or blood samples were processed with CTC enrichment, on-slide cell fixation and immunostaining, followed by imaging all cells in bright field and fluorescent colors. For ascites samples, HK2^high^/CK^+^/DAPI^+^ cells were identified as sCTCs. For peripheral blood samples, HK2+CK^high^/CD45^−^ cells were identified as sCTCs. These sCTCs were then retrieved for single-cell low-pass WGS. Details on sample processing, sCTC detection, single-cell low-pass WGS, and CNA identification are provided in the following Methods.

### Ascites processing and the single-cell metabolic test

Received ascites samples (10 mL) were filtered by a membrane with a pore size of 150 μm and centrifuged at 300 g for 10 min to separate cell pellets. Cells were treated with red blood cell (RBC) lysing buffer (BD Biosciences) to remove RBCs, followed by processing with a membrane with pore size of 5 μm (Supplementary Fig. 6) if the number of remaining cells was greater than 0.5 million. Cells were then re-suspended in 0.5 mL of PBS and mixed with 0.5 mL of DSP/SPDP in PBS (5 mM). DSP and SPDP were dissolved in anhydrous DMSO at 50 mM as stock solutions. The cell suspension was added into a chamber for cell fixation and sedimentation. After 30 min, the fixed cells were prepared as a cell monolayer on a poly-L-lysine glass slide by 5 min of centrifuging at 110 g, and incubated with 100 μl of Tris-HCl (100 mM) at room temperature for 10 min, followed by washing with PBS for three times. On-slide cells were blocked with 3% BSA and 10% Normal Goat Serum for 1 h, followed by immunostaining with anti-HK2 antibody (1:100 dilution), anti-pan-CK antibody eFluor 570 (1:200 dilution) and anti-CD45-APC (1:50 dilution) in 0.1% BSA/PBS overnight at 4 °C. After extensive washing with PBS, cells were treated with Alexa Fluor 488-conjugated secondary antibody (1:400 dilution) at room temperature for 1 h and DAPI for 5 min. After PBS washing, ImageXpress Micro XLS Wide field High Content Screening System (Molecular Devices) scanned the glass slides and imaged all cells in bright field and fluorescent colors. HK2^high^/CK^+^/DAPI^+^ cells were identified as sCTCs, followed by retrieved with a motorized micromanipulator (XenoWorks). The HK2^high^ threshold is defined by the average HK2 intensity plus five times the standard deviation (SD) of CK^−^ cells (CK intensity <400).

### Peripheral blood-based CTC detection

Fresh blood samples (5 mL) were drawn and preserved in TransFix/EDTA Vacuum Blood Collection Tubes (Cytomark). Blood samples were initially centrifuged at 500 g for 5 min. The supernatant was discarded and the cell pellet was re-suspended in an equivalent volume of HBSS and mixed with 25 μl antibody cocktail (RosetteSep^TM^ CTC Enrichment Cocktail, Stemcell Technologies) at room temperature for 20 min, followed by adding 15 ml of HBSS with 2% FBS and mixing well. The mixture was carefully added along the wall of the Sepmate tube (SepMate^TM^-50) after adding 15 ml density gradient liquid (Lymphoprep^TM^) into the tube through the middle hole. After centrifuging at 1200 g for 20 min, the topmost supernatant (~10 ml) was discarded, and the remaining liquid (~10 ml) above the barrier of the Sepmate tube was rapidly poured out into a new centrifuge tube. After centrifuging at 600 g for 8 min, the supernatant was removed and 1 ml of RBC lysing buffer was then added for 5 min to lyse RBCs. After centrifuging at 250 g for 5 min, the nucleated cell pellet was re-suspended in HBSS containing DSP (5 mM). The cell suspension was applied onto a 3% BSA-treated poly-L-lysine glass slide. A 30 min waiting period was allowed for cell fixation and cells sitting down to the slide. After DSP-based cell fixation, cells on the slide were blocked with a blocking solution containing 3% BSA and 10% Normal Goat Serum for 1 h, followed by incubation with APC-conjugated anti-CD45 antibody (mouse), Alexa Fluor 488-conjugated anti-pan-CK (mouse) and anti-HK2 antibody (rabbit) in PBS overnight at 4 ^o^C. After extensive washing with PBS, cells on the chip were treated with Alexa Fluor 488-conjugated goat-anti-rabbit secondary antibody in PBS for 1 h and DAPI for 10 min followed by washing with PBS. ImageXpress Micro XLS Wide field High Content Screening System (Molecular Devices) scanned the chip and imaged all cells in bright field and fluorescent colors (CD45: CY5; HK2 + CK: FITC; Nucleus: DAPI). A computational algorithm analyzed the images and identified HK2+CK^high^/CD45^−^ cells as sCTCs based on the cut-off of 5 SD above the mean HK2 + CK fluorescence intensity of CD45^+^ leukocytes in the samples.

### Single-cell WGS for genome-wide CNA profiling using MALBAC

Single-cell low-pass WGS was used to characterize genome-wide CNA profiles. Single-cell whole genome amplification (WGA) was firstly conducted with the MALBAC® Single Cell WGA Kit (Yikon Genomics). To assess the WGA coverage of amplified product, 22 primer pairs were designed to target 22 loci located on different chromosomes (Supplementary Table 1). Six primer pairs were randomly selected for PCR and successful amplification of at least four out of six primer pairs generated a positive quality control (QC)-PCR. WGA products that passed QC-PCR were then used to construct WGS library with the NEBNext® Ultra™ DNA Library Prep Kit for Illumina (New England Biolabs) or MGIEasy Universal DNA Library Prep Kit (MGI Tech) according to the manufacturer’s protocol. The concentrations of purified fragmented DNA or libraries were quantified with Qubit dsDNA HS Assay Kit (Invitrogen). Libraries were analyzed by Illumina Navoseq6000 with 150 bp pair-end reads (Genewiz, China) or MGI2000 sequencer with 100 bp single-end read (JunHealth, China). FASTQ files were aligned to the major chromosomes of human (hg19) using BWA (version 0.7.17) with default options. PCR duplicates were removed with Samtools (version 1.11). Aligned reads were counted in fixed bins averaging 500 kb. Bin counts were normalized for GC content with lowess regression and bin-wise ratios were calculated by computing the ratio of bin counts to the sample mean bin count. The diploid regions were determined using HMMcopy (version 0.1.1). Segmentation was performed with circular binary segmentation (CBS) method (alpha=0.0001 and undo.prune=0.05) from R Bioconductor ‘DNAcopy’ package. Copy number noise was quantitated using the mean absolute pairwise difference (MAPD) algorithm. MAPD reliably measures the quality of the amplified genome, and high MAPD scores associate with poor-quality samples. Samples with MAPD ≤ 0.45 (500 kb bin size) passed the MAPD QC and were included in single-cell genome-wide CNA analyses. The success rate of the single-cell WGS is defined as the percentage of sequenced sCTCs that pass the two-step QC procedure (QC-PCR and MAPD QC).

### Tn5 transposome assembly

Commercial Tn5 transposase was purchased from Novoprotein (China). Transposon DNA oligonucleotides were synthesized by Genewiz (China) and diluted with annealing buffer to a concentration of 100 mM. To form Tn5 transposome, Tn5-ME (CTGTCTCTTATACACATCT, 10 μl) and Tn5-adaptor1 (TCGTCGGCAGCGTCAGATGTGTATAAGAGACAG, 10 μl) or Tn5-adaptor2 (GTCTCGTGGGCTCGGAGATGTGTATAAGAGACAG, 10 μl) oligonucleotides were mixed together at an equimolar ratio, and annealed by gradual cooling (75 ^o^C 15 min, 60 ^o^C 10 min, 50 ^o^C 10 min, 40 ^o^C 10 min, and 25 ^o^C 30 min). The preannealed transposon oligonucleotides mixture (4 μl) was subsequently mixed with 20 μl of Tn5 transposase (10 mM), followed by incubation for 1 h at room temperature. The assembled Tn5 transposome were stored at −20 ^o^C.

### Single-cell WGS for genome-wide CNA profiling using Tn5 transposome

For single-cell WGS, a sCTC was identified and collected by a micromanipulator (Eppendrof TransferMan 4r) into a low binding PCR tube (200 μl, Axygen) containing 2.0 μl of cell lysis buffer (60 mM Tris-Ac pH 8.3, 2 mM EDTA pH 8.0, 15 mM DTT, 0.5 uM carrier ssDNA, 20 mg/uL QIAGEN protease). The PCR tube was incubated at 55 ^o^C for 1 h to lyse the cell and release genomic DNA, followed by denaturing protease at 85 ^o^C for 15 min. Exposed genomic DNA was tagmented by adding 0.5 μl of Tn5 transposome (1:200 diluation) that introduced PCR adaptor to DNA fragments. After DNA fragmentation, gap filling of fragmented genomic DNA was conducted at 72 ^o^C for 10 min with NEBNext® Ultra™ II Q5^®^ Master Mix (#M0544, New England Biolabs), followed by polymerase-based fragmented DNA and library amplification. PCR condition was 98 ^o^C for 1 min, 20 cycles of 98 ^o^C for 10 s, 60 ^o^C for 15 s and 72 ^o^C for 30 s. Amplified single-cell sequencing library was purified with Agencourt^®^ AMPure XP beads. High-throughput sequencing was conducted on NovaSeq 6000 (PE150).

### CNA burden calculation

CNA burden is defined as the percentage of the tumor autosomal genome with copy number altered. To calculate CNA burden for a sample, segments of copy number gains and losses are determined (see Code Availability for codes), and their total genomic length is summed and calculated as a percentage of the size of the autosomal genome.

### Cell lines

All lung cancer cell lines (H1650, H1975, HCC827, H2228, PC-9) used in this study were obtained from American Type Culture Collection (ATCC) and bladder cancer cell line RT4 was purchased from Cell Bank of the Chinese Academy of Sciences (Shanghai, China). Cell lines were routinely maintained in ATCC-formulated cell culture medium containing 10% fetal bovine serum (FBS, Gibco) and 1× Penicillin-Streptomycin-Glutamine (Gibco) in a humidified atmosphere of 5% CO_2_ and 95% air at 37 ^o^C. Cell lines were authenticated by DNA short tandem repeat (STR) profiling analysis and were tested negative for mycoplasma contamination. RT4 is listed in the database of commonly misidentified cell lines. RT4 cells were used in the study for validating the single-cell WGS protocols rather than biological function investigation.

### Statistical analysis

The normality of the data was tested by the Kolmogorov-Smirnov test. Data without normal distribution was presented as median with inter-quartile range (IQR). The Mann-Whitney test was performed for the non-parametric test between two groups that were not normally distributed. The receiver operating characteristic (ROC) curve was generated to compute the area under the curve (AUC) with 95% Wald confidence interval (CI). All statistical analyses were performed with GraphPad Prism 8.

### Reagents

Anti-HK2 primary antibody was obtained from Abcam (#ab209847). Pan Cytokeratin Monoclonal Antibody (AE1/AE3), eFluor 570 (#41-9003-82), Pan Cytokeratin Monoclonal Antibody (AE1/AE3), Alexa Fluor 488 (#53-9003-82), Alexa Fluor 488-conjugated goat-anti-rabbit secondary antibody (#A11008), APC-conjugated anti-CD45 (#17-0459-42), Dithiobis (succinimidyl propionate) (DSP, #22585), Succinimidyl 3-(2-pyridyldithio) propionate (SPDP, #21857) and Anhydrous DMSO (#D12345) were obtained from Thermo Fisher Scientific. RosetteSep CTC Enrichment Cocktail Containing Anti-CD36 (#15167), SepMate^TM^-50 (#85450) and Lymphoprep^TM^ (#07801) were purchased from STEMCELL Technologies. DAPI (#C1006) was obtained from Beyotime Biotechnology. BSA (#B2064) was obtained from Sigma. MALBAC Single Cell WGA Kit (#KT110700150) was purchased from Yikon Genomics. NEBNext Ultra DNA Library Prep Kit for Illumina (#E7645L) and NEBNext® Ultra™ II Q5^®^ Master Mix (#M0544) were purchased from New England Biolabs. MGIEasy Universal DNA Library Prep Kit (#1000017571) was obtained from MGI Tech. Qubit dsDNA HS Assay Kit (#Q32854) was obtained from Invitrogen. Red blood cell lysing buffer (#555899) was obtained from BD Biosciences. Agencourt® AMPure XP beads (#A63881) were purchased from Beckman Coulter. Poly-L-lysine glass slides (#P4981-001) were purchased from Epredia. Normal Goat Serum (#E510009-0100) was obtained from Sangon Biotech.

### Reporting summary

Further information on research design is available in the Nature Research Reporting Summary linked to this article.

### Supplementary information

Supplementary Information

Reporting summary

## Data Availability

Single-cell WGS data and bulk DNA WGS data of tumor tissues generated in this study have been deposited in the Genome Sequence Archive (GSA) under accession number HRA004452. Other data are available in the main text or the supplementary materials.

## Code availability

R scripts for single-cell CNA analysis is accessible on GitHub at the following URL: https://github.com/Shilab-wangzhuo/CNV.
