# Supplementary material for: Single-cell low-pass whole genome sequencing accurately detects circulating tumor cells for liquid biopsy-based multi-cancer diagnosis
Source: NPJ Precis Oncol. 2024 Feb 6;8:30. doi: 10.1038/s41698-024-00520-1 (PMC10847465; doi:10.1038/s41698-024-00520-1)
Supplement: Supplementary file 1 — Supplementary Information [file 41698_2024_520_MOESM1_ESM.pdf]

Supplementary Information

Single-cell low-pass whole genome sequencing accurately detects circulating tumor cells for liquid biopsy-based multi-cancer diagnosis

Xiaohan Shen[1]†, Jiao Dai[2]†, Lingchuan Guo[3]†, Zhigang Liu[1], Liu Yang[4], Dongmei Gu[3], Yinghong Xie[3], Zhuo Wang[1], Ziming Li[5]*, Haimiao Xu[2]*, Qihui Shi[1,6]*

[1]Key Laboratory of Whole-Period Monitoring and Precise Intervention of Digestive Cancer (SMHC), Minhang Hospital and Shanghai Key Laboratory of Medical Epigenetics, Institutes of Biomedical Sciences, Fudan University, Shanghai, 200032, China; [2]Department of Pathology, Zhejiang Cancer Hospital, Hangzhou Institute of Medicine (HIM), Chinese Academy of Sciences, Hangzhou, Zhejiang 310022, China; [3]Department of Pathology, The First Affiliated Hospital of Soochow University, Suzhou, 215000, China; [4]Shanghai Bone Tumor Institute and Department of Orthopedics, Shanghai General Hospital, Shanghai Jiao Tong University School of Medicine, Shanghai, 200080, China; [5]Shanghai Lung Cancer Center, Shanghai Chest Hospital, Shanghai Jiao Tong University, Shanghai, 200030, China; [6]Shanghai Engineering Research Center of Biomedical Analysis Reagents, Shanghai, 201203, China.

†These authors contributed equally to this work.

*Correspondence authors: Q. S. (qihuishi@fudan.edu.cn) or H. X. (xuhm@zjcc.org.cn) or Z L. (liziming1980@hotmail.com)

I. Supplementary Figures 1-53

II. Supplementary Tables 1-8

Supplementary Figures

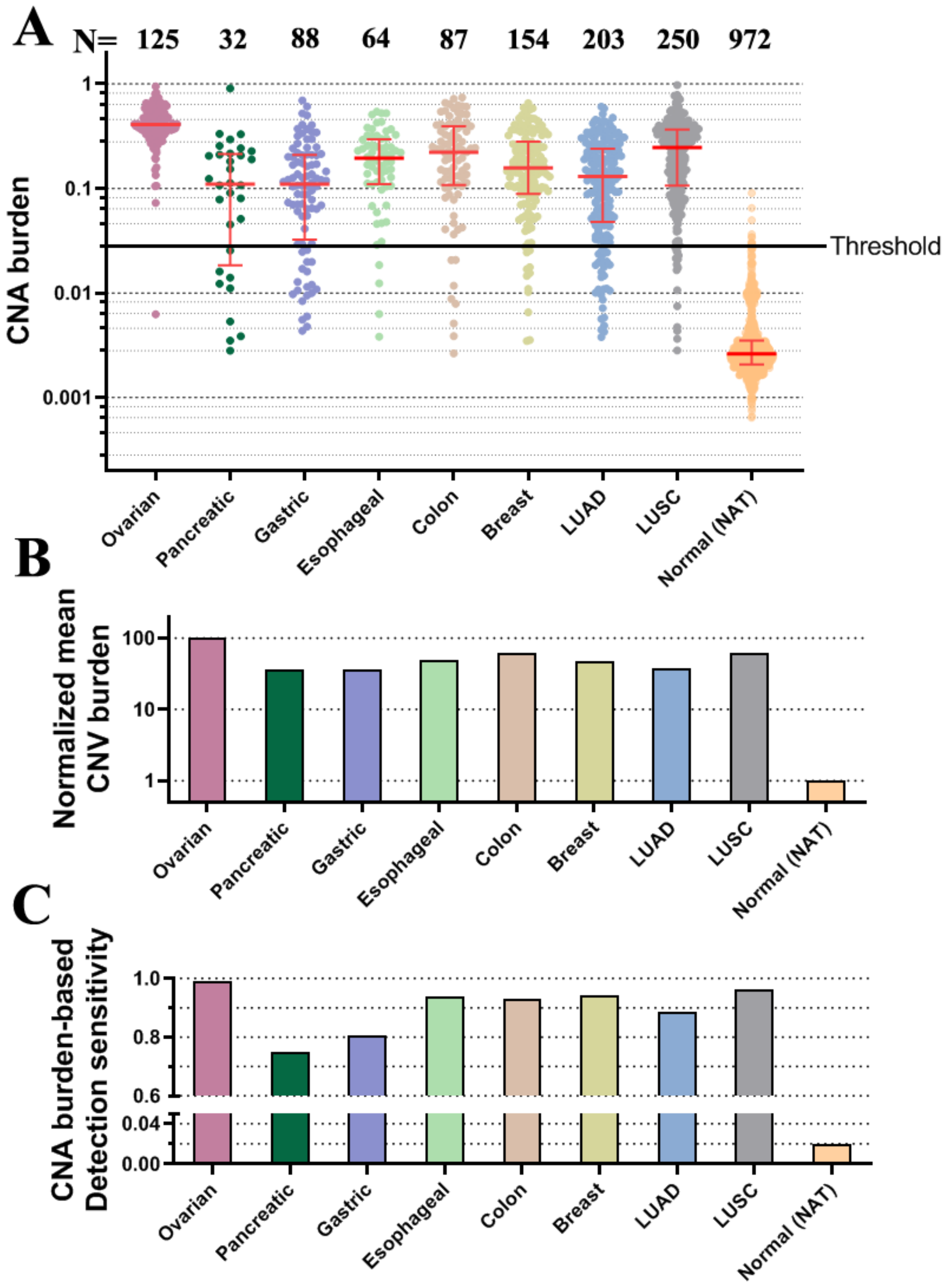

Supplementary Figure 1. (A) Calculated CNA burden of eight types of cancers and matched normal controls (normal tissue adjacent to the tumor, NAT) from Genomic Data Commons; (B) Normalized mean CNA burden of eight types of cancers and matched normal controls (NAT); (C) CNA-based detection sensitivity at the threshold of CNA burden at a threshold of 0.02.

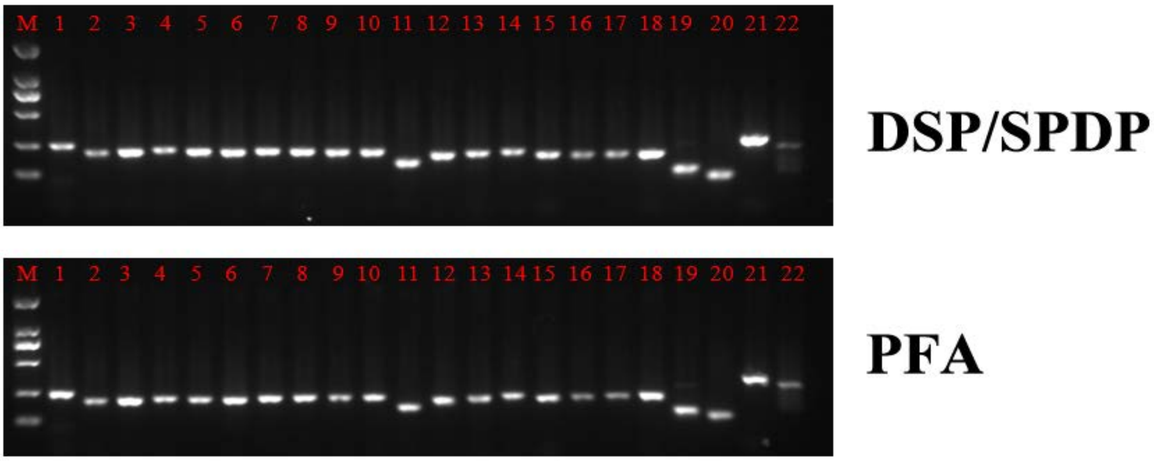

Supplementary Figure 2. For assessing the quality of single-cell WGS, 22 primer pairs (see Table S1) were designed to target 22 loci located on different chromosomes. Fresh RT4 cells (bladder cancer cell line) were fixed with different cell fixation methods (DSP/SPDP method and 4% PFA), followed by single-cell whole genome amplification (WGA) and PCR reaction. No immunofluorsence staining and storage were conducted. Results show that no significant difference in WGA quality was found between different cell fixation methods.

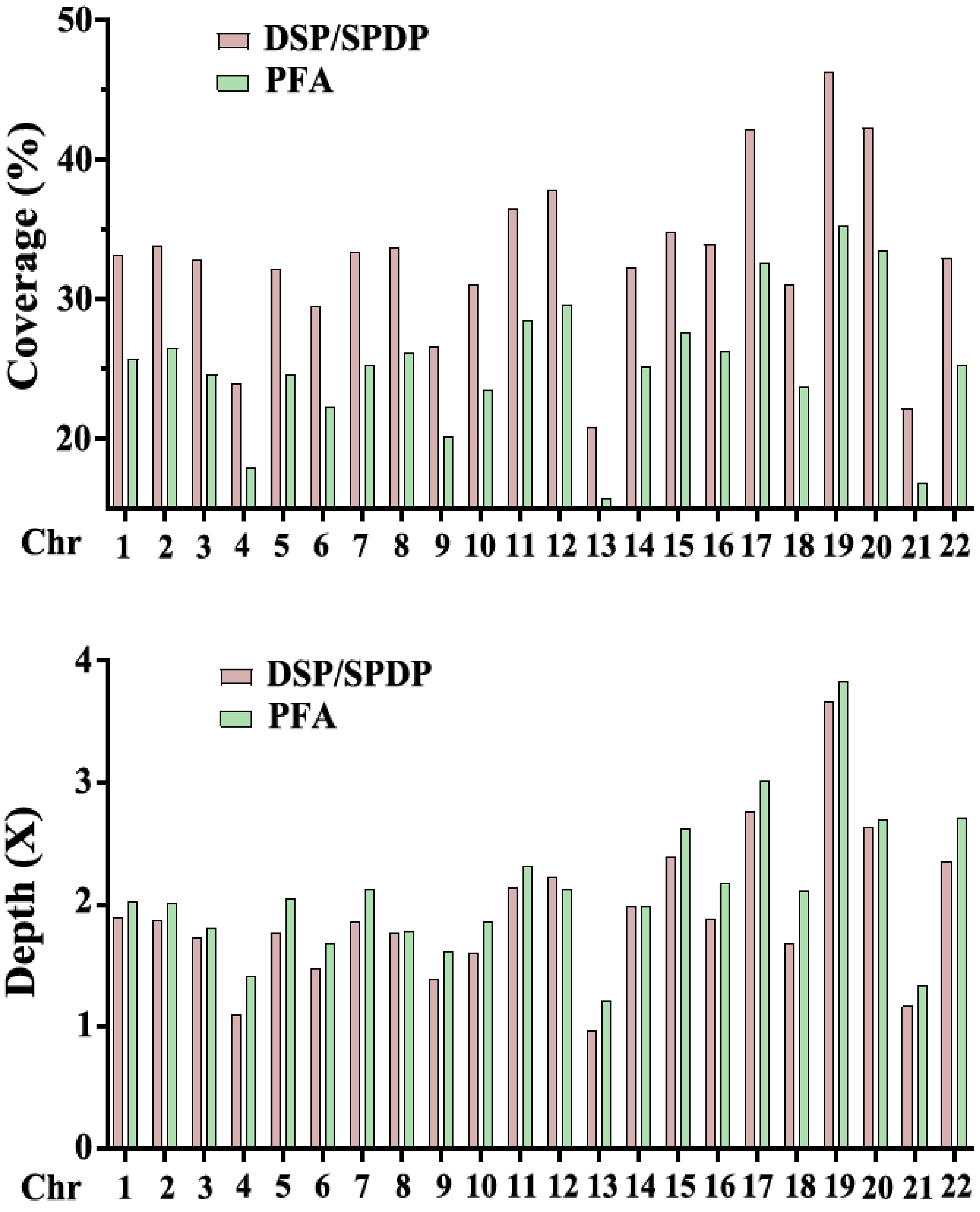

Supplementary Figure 3. Fresh RT4 cells were fixed with different cell fixation methods (DSP/SPDP method and 4% PFA), followed by immunofluorescence staining according to the protocol and stored at 4°C for 48 h. To assess the quality of single-cell WGS between two cell fixation methods, single-cell whole genome amplification, library preparation and sequencing were then performed. Sequencing coverage (top) and depth (bottom) of both methods are shown in the figure. Average sequencing depths of DSP/SPDP and PFA methods are 1.9 and 2.1, respectively (bottom). Average coverage of DSP/SPDP and PFA methods are 33% and 25%, respectively (top).

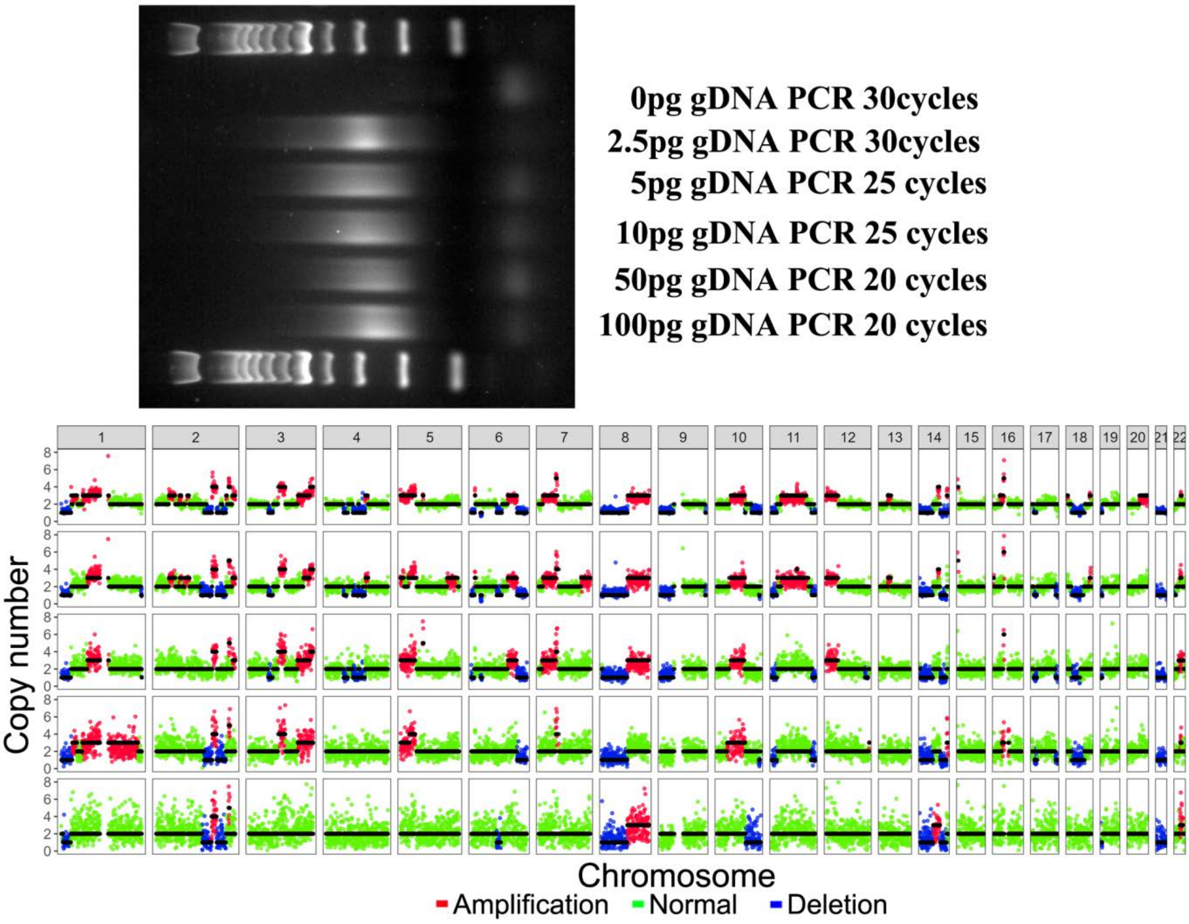

Supplementary Figure 4. Gel electrophoresis (top) and CNA profiles (bottom) based on the Tn5-based protocol from 100pg, 50pg, 10pg, 5pg, and 2.5pg of genomic DNA extracted from PC-9 cells, respectively.

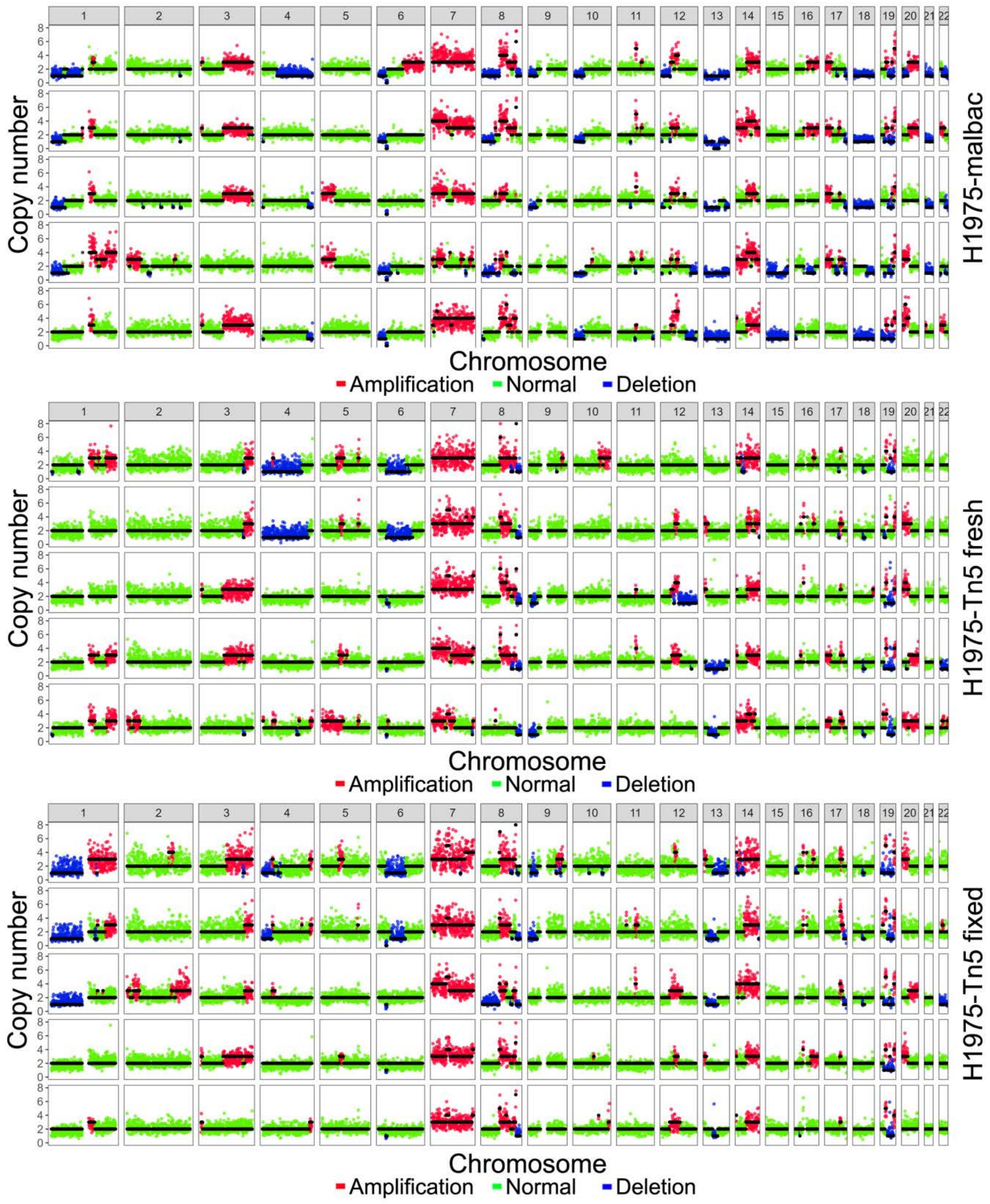

Supplementary Figure 5. Genome-wide CNA profiles derived from single-cell WGS of 5 DSP/SPDP-fixed H1975 single cells with the commercial MALBAC kit (top), 5 fresh H1975 single cells (middle) and 5 DSP/SPDP-fixed H1975 single cells (bottom) with Tn5-based protocol developed in this study.

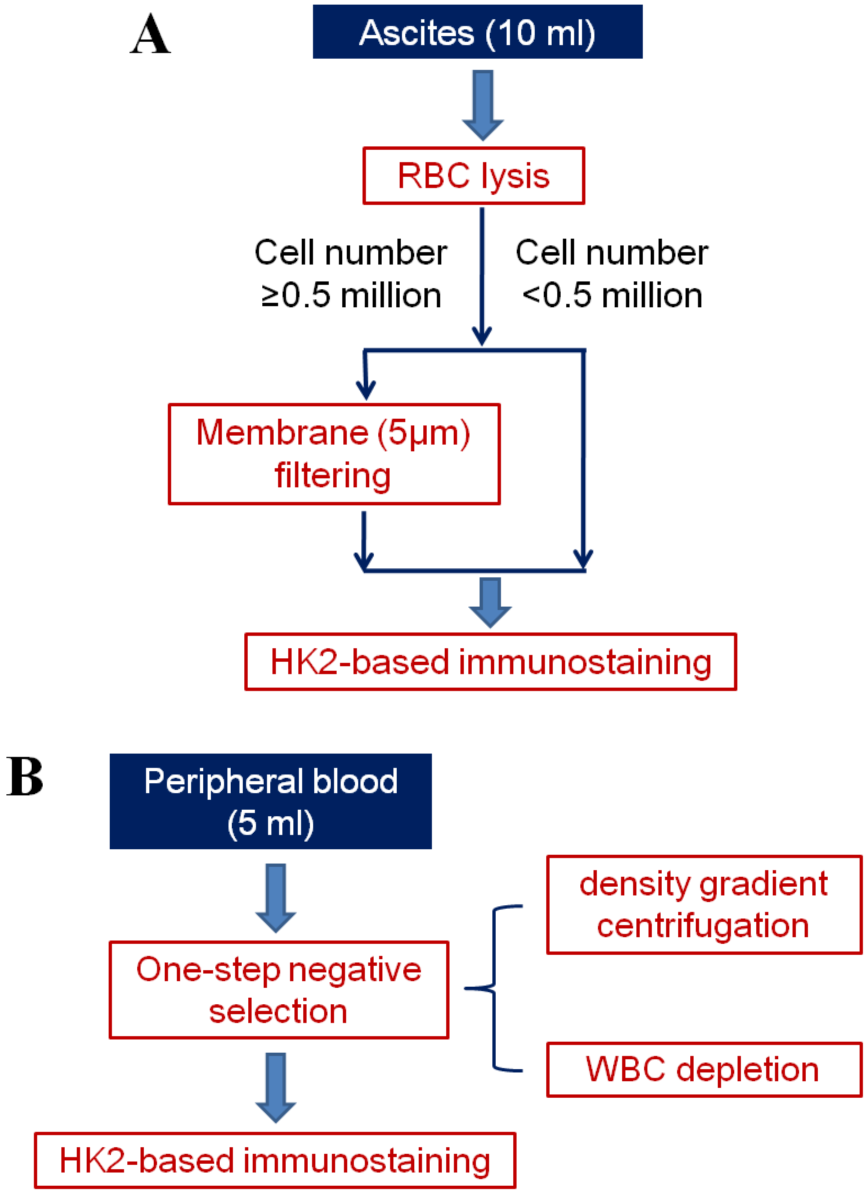

Supplementary Figure 6. Sample processing schemes for ascites (top) and peripheral blood (bottom). See Methods for detailed protocols.

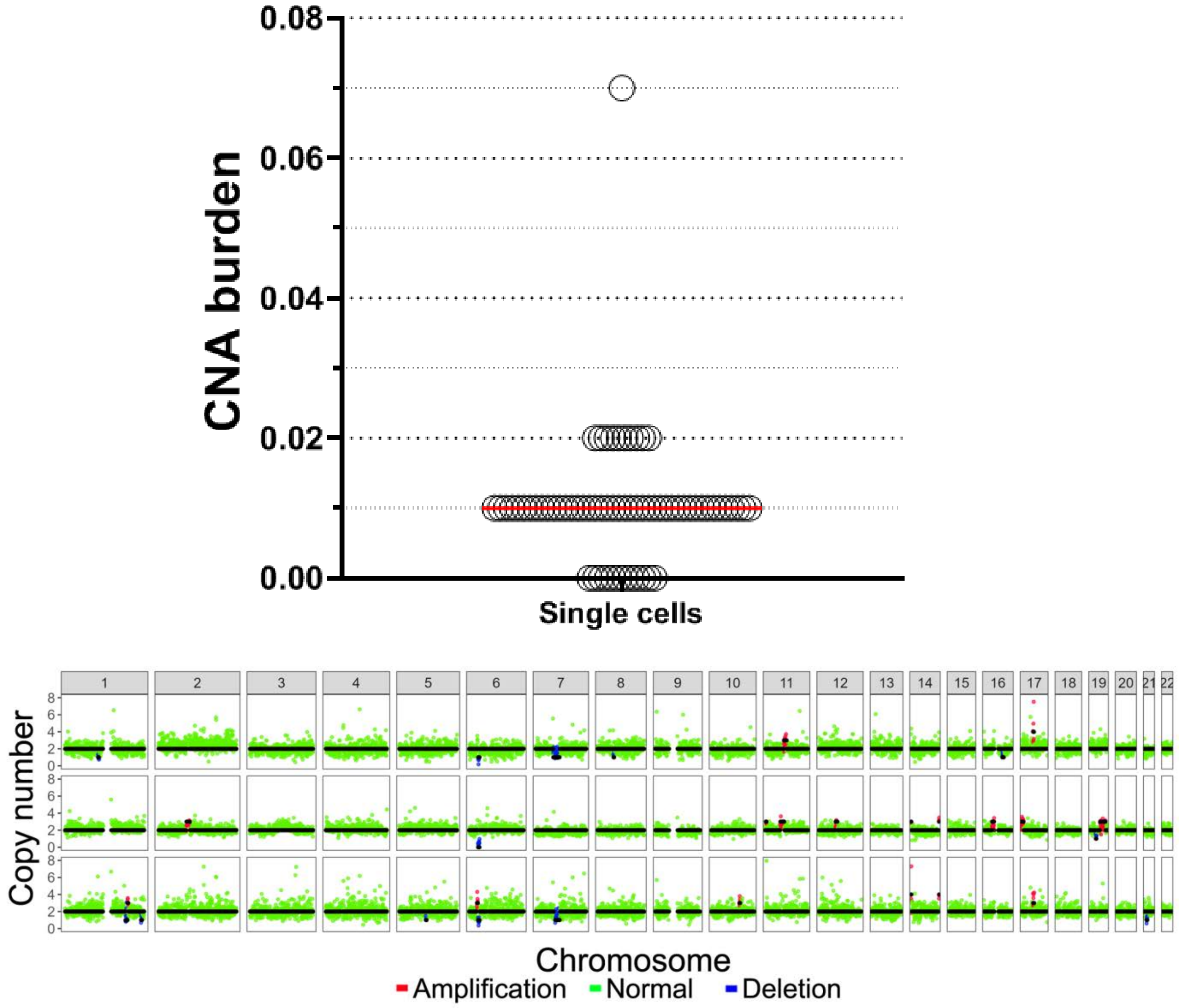

Supplementary Figure 7. Top, CNA burden of 67 CK^{+} cells from 14 patients in BA group of ascites training cohort, including 45 sCTCs from 9 BA patients and 22 randomly selected CK^{+} cells from 5 BA patients. A CNA burden threshold of normal cells was then determined at 0.02. Bottom, representative single-cell CNA profiles of CK^{+} cells with the CNA burden of 0.02.

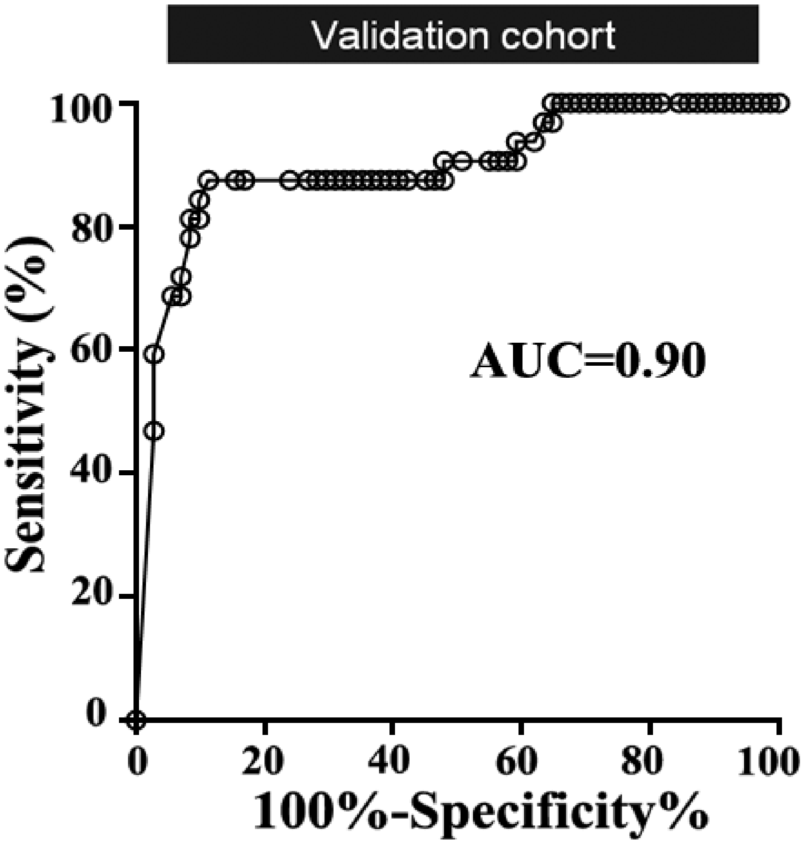

Supplementary Figure 8. ROC curve generated from sCTCs counts in the validation cohort of the ascites study. The AUC was computed to be 0.90.

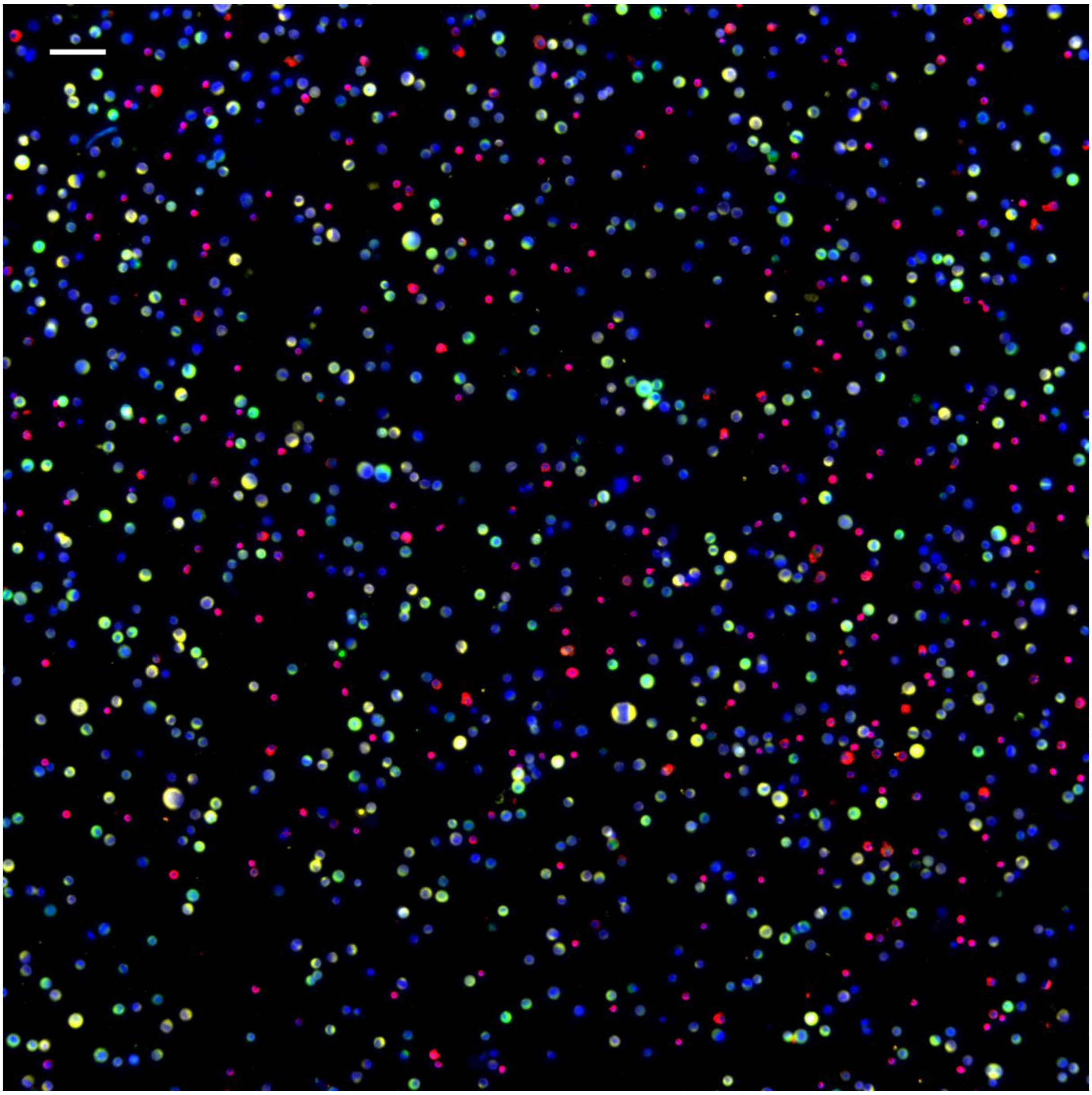

Supplementary Figure 9. Typical overlaid fluorescence image of cells from a malignant ascites sample (P30, Green: HK2; Yellow: CK; Red: CD45; Blue: DAPI). Scale bar: 100 μm.

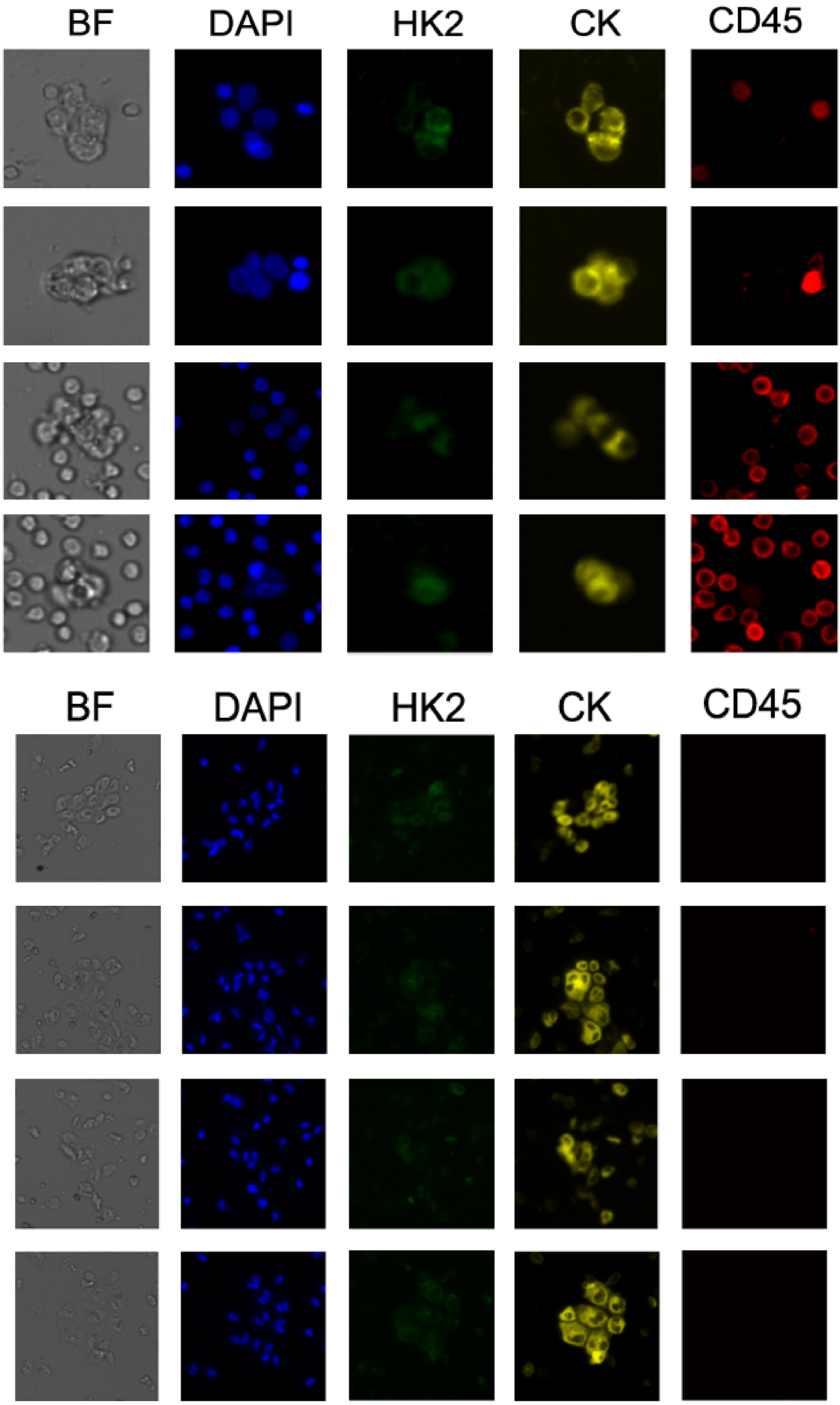

Supplementary Figure 10. Images of cell clusters found in ascites samples from patients with benign diseases. These clusters were $HK2^{low}/CK^{+}/CD45^{-}/DAPI^{+}$. Top, B3; Bottom, B27. BF, bright field.

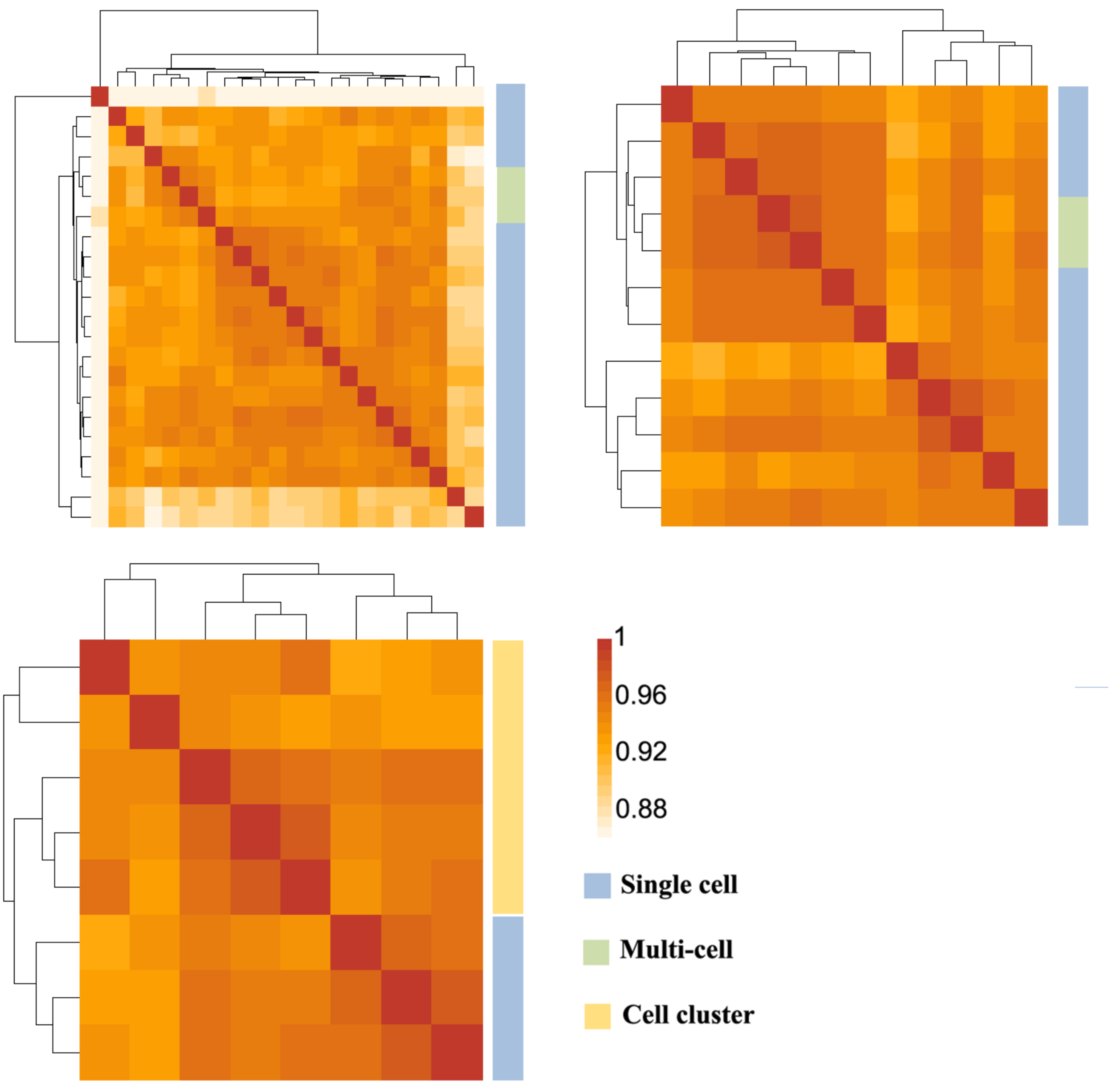

Supplementary Figure 11. Heat maps of correlation coefficients of CNA profiles between single CTCs, multi-CTC pools and CTC clusters from P30 (top left), P3 (top right) and P67 (bottom left). The CNA profiles are shown in Figure 4B-4D.

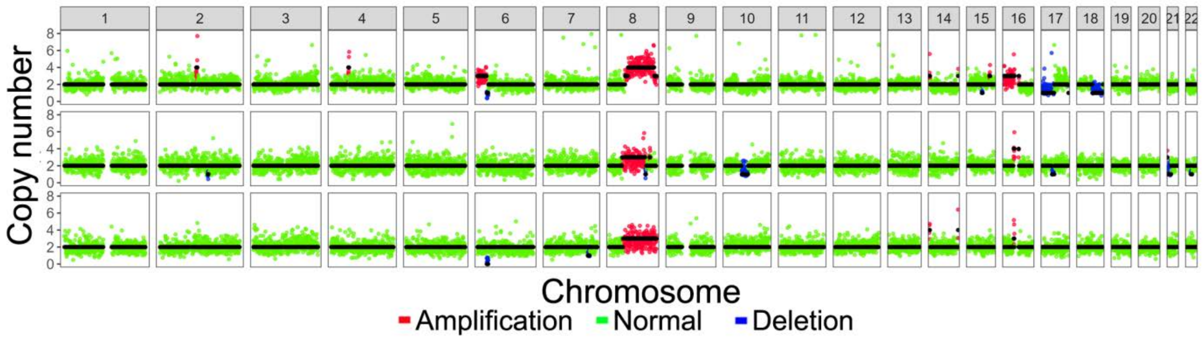

Supplementary Figure 12. Single-cell CNA profiles of 3 single HK2-derived CTCs across the genome from P1 (ovarian cancer).

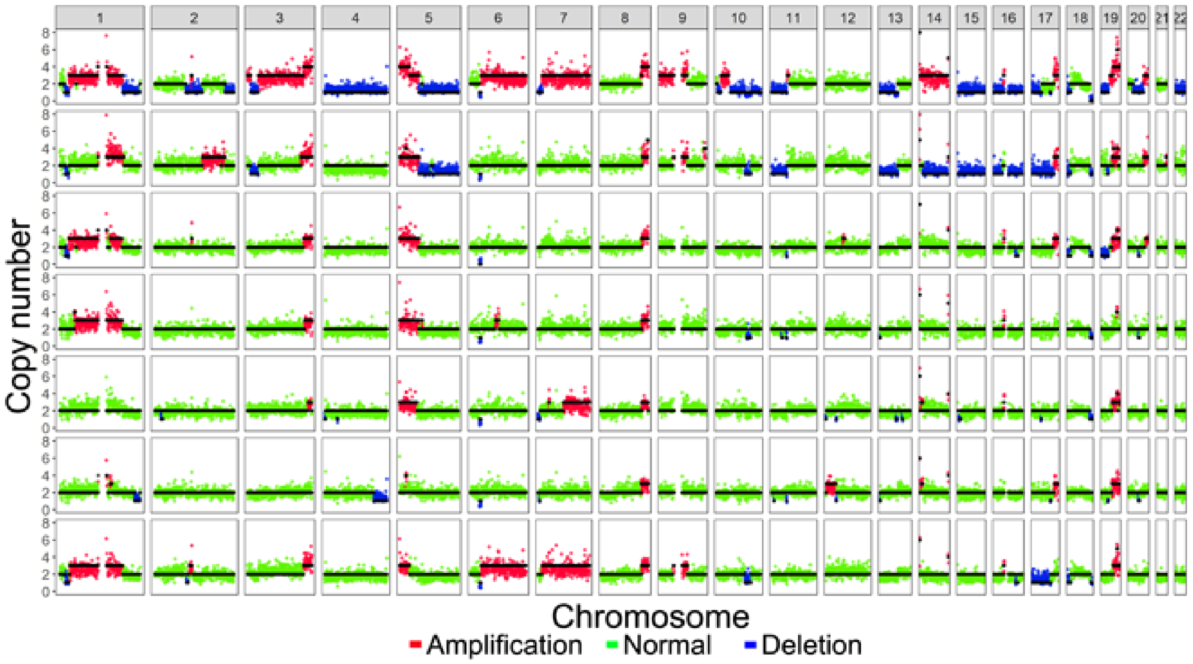

Supplementary Figure 13. Single-cell CNA profiles of 6 single HK2-derived CTCs and a 3-CTC pool (last) across the genome from P2 (ovarian cancer).

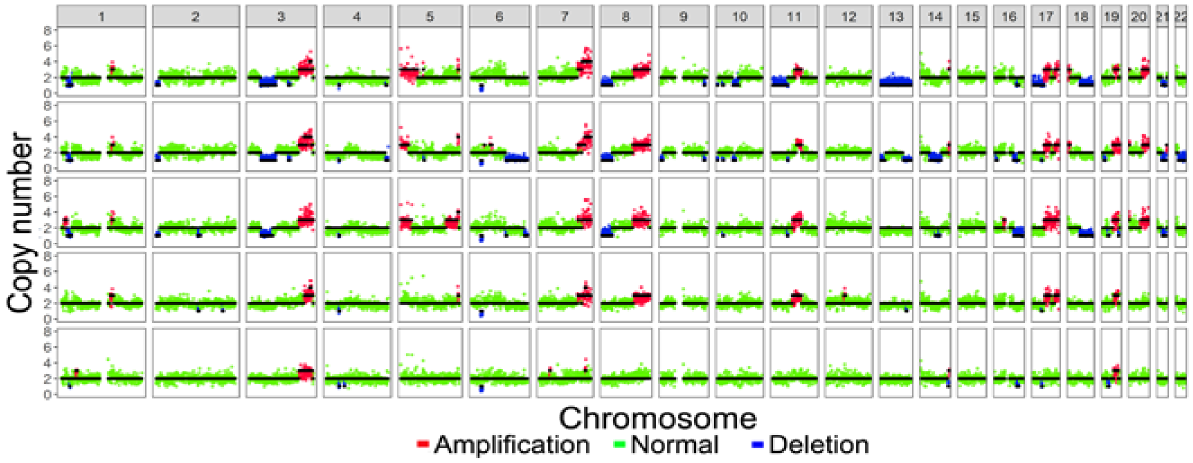

Supplementary Figure 14. Single-cell CNA profiles of 5 single HK2-derived CTCs across the genome from P4 (ovarian cancer).

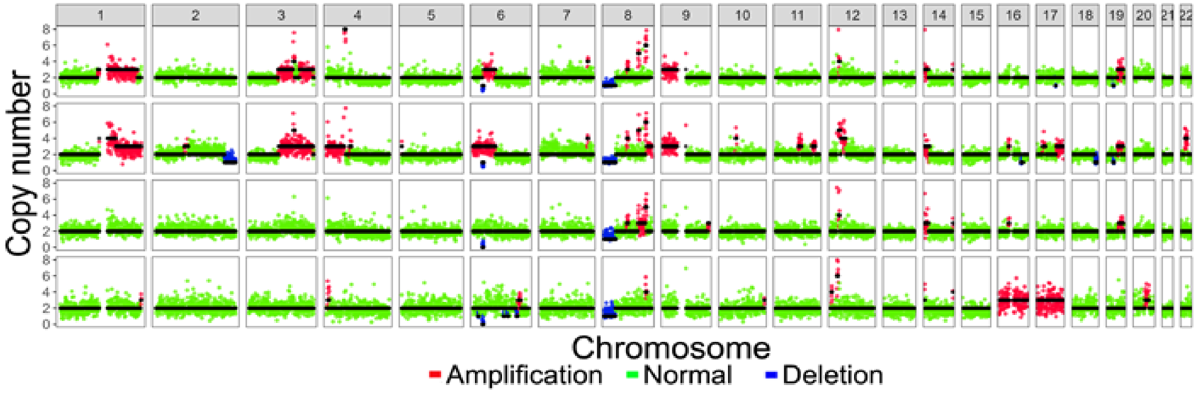

Supplementary Figure 15. Single-cell CNA profiles of 4 single HK2-derived CTCs across the genome from P6 (ovarian cancer).

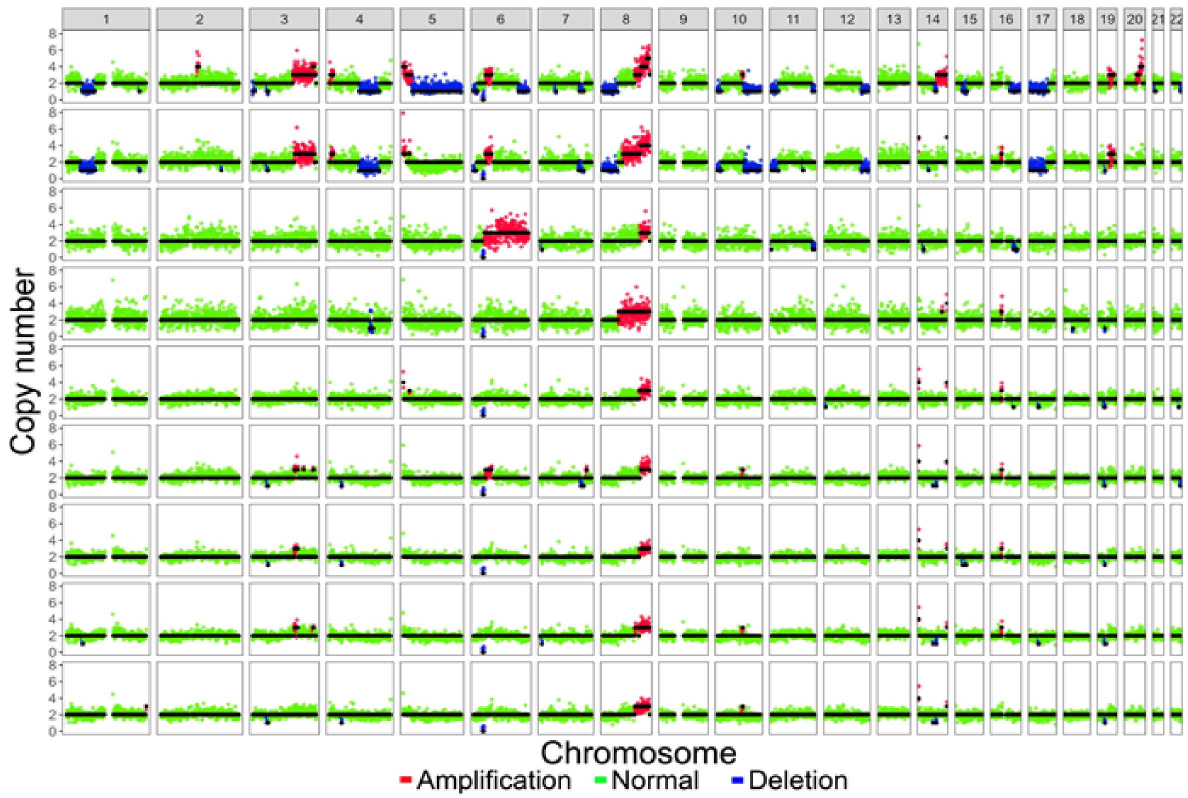

Supplementary Figure 16. Single-cell CNA profiles of 9 single HK2-derived CTCs across the genome from P7 (ovarian cancer).

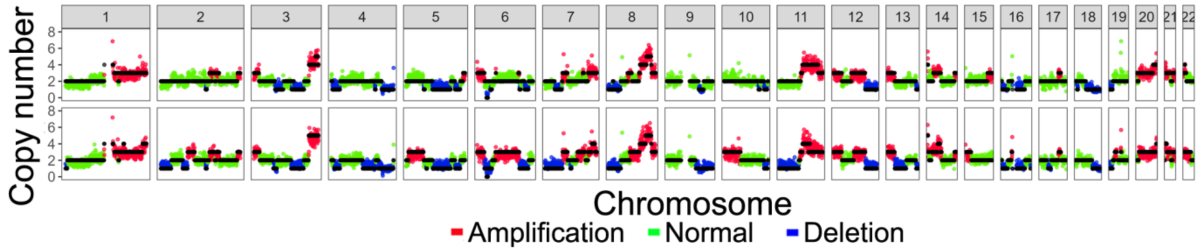

Supplementary Figure 17. Single-cell CNA profiles of 2 single HK2-derived CTCs across the genome from P9 (ovarian cancer).

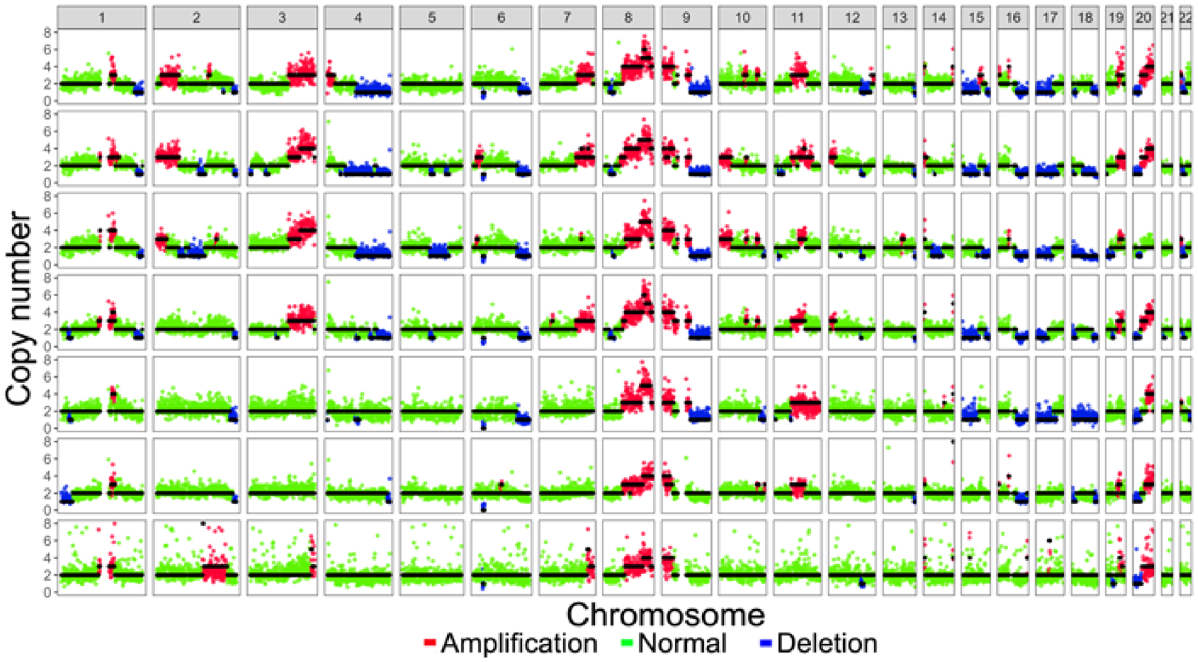

Supplementary Figure 18. Single-cell CNA profiles of 7 single HK2-derived CTCs across the genome from P10 (ovarian cancer).

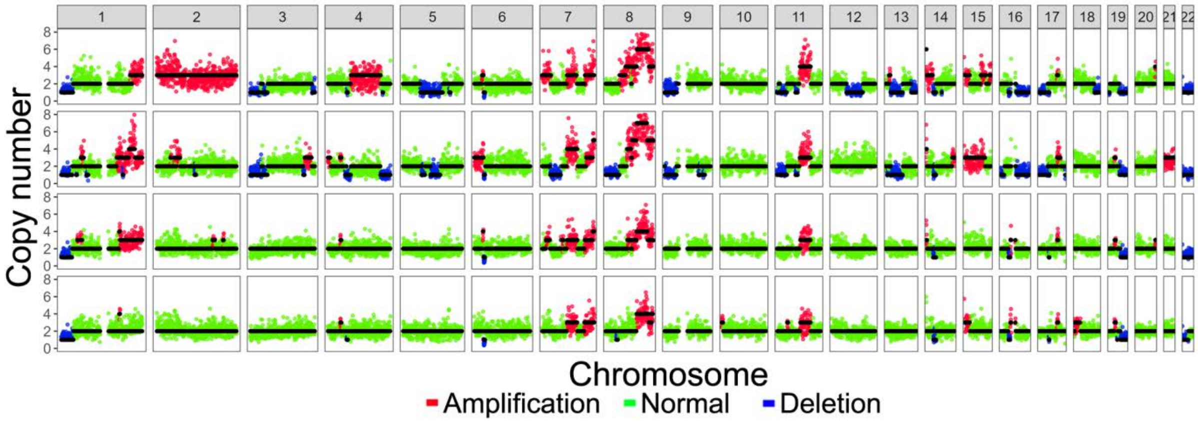

Supplementary Figure 19. Single-cell CNA profiles of 4 single HK2-derived CTCs across the genome from P11 (ovarian cancer). The ascites cytology showed a negative result. Single-cell genome sequencing provides compelling evidence of cell malignancy that generates a MA diagnosis.

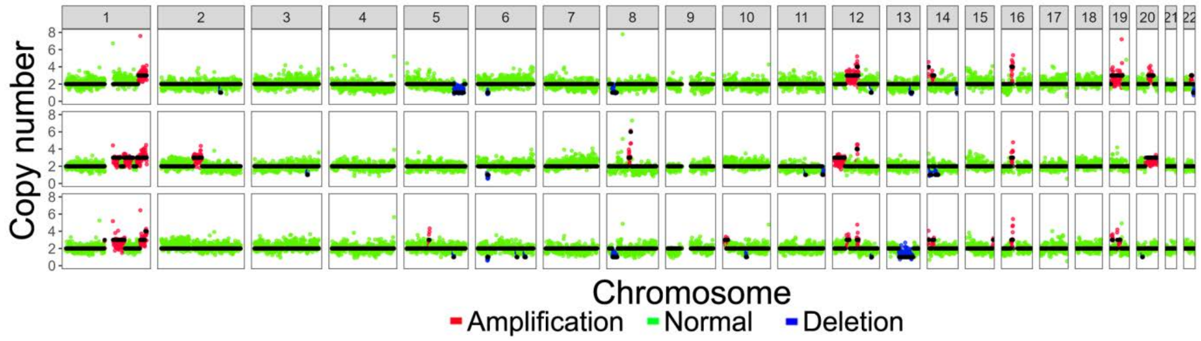

Supplementary Figure 20. Single-cell CNA profiles of 3 single HK2-derived CTCs across the genome from P12 (ovarian cancer).

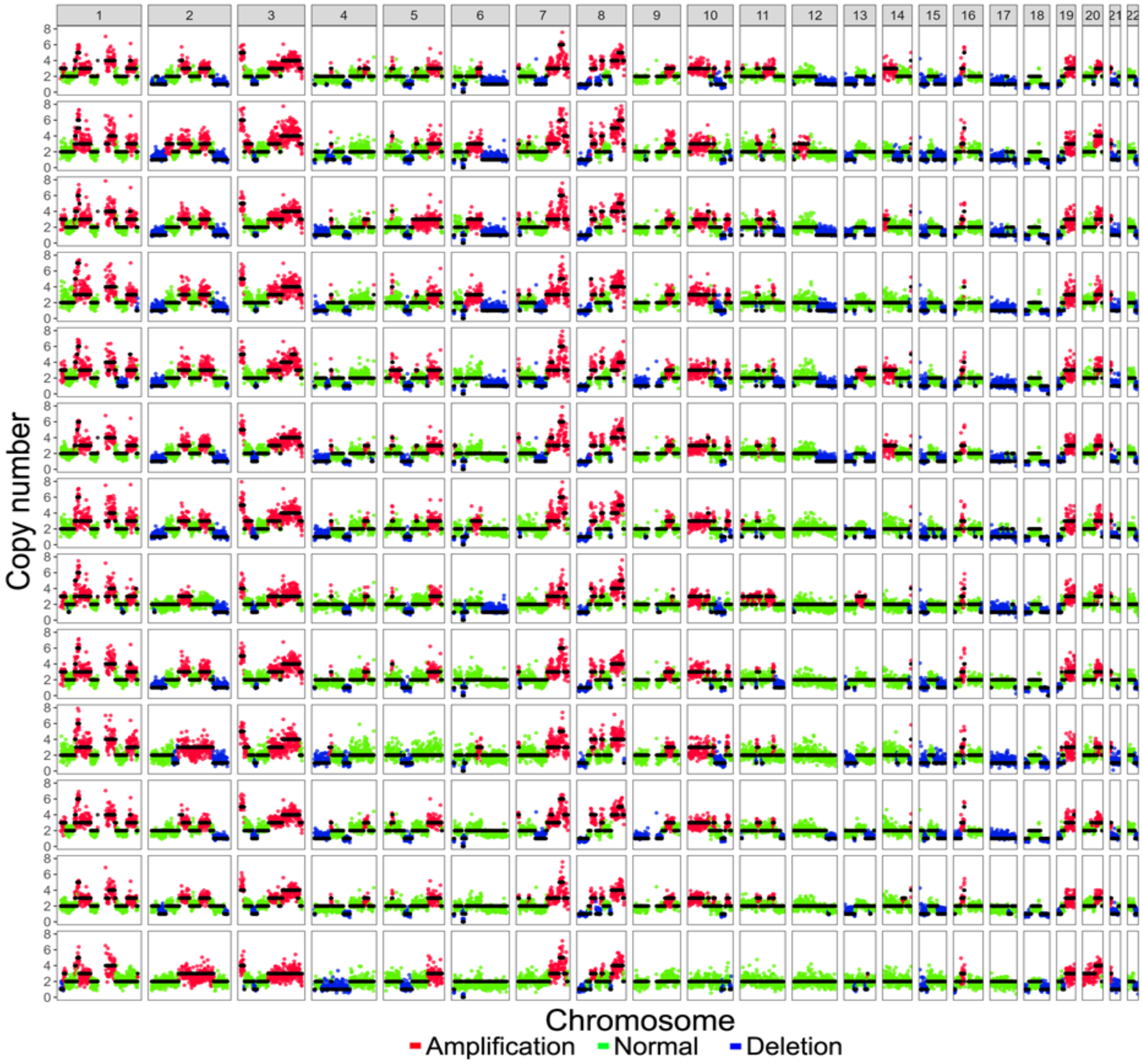

Supplementary Figure 21. Single-cell CNA profiles of 13 single HK2-derived CTCs across the genome from P13 (ovarian cancer). The ascites cytology showed a negative result. Single-cell genome sequencing provides compelling evidence of cell malignancy that generates a MA diagnosis.

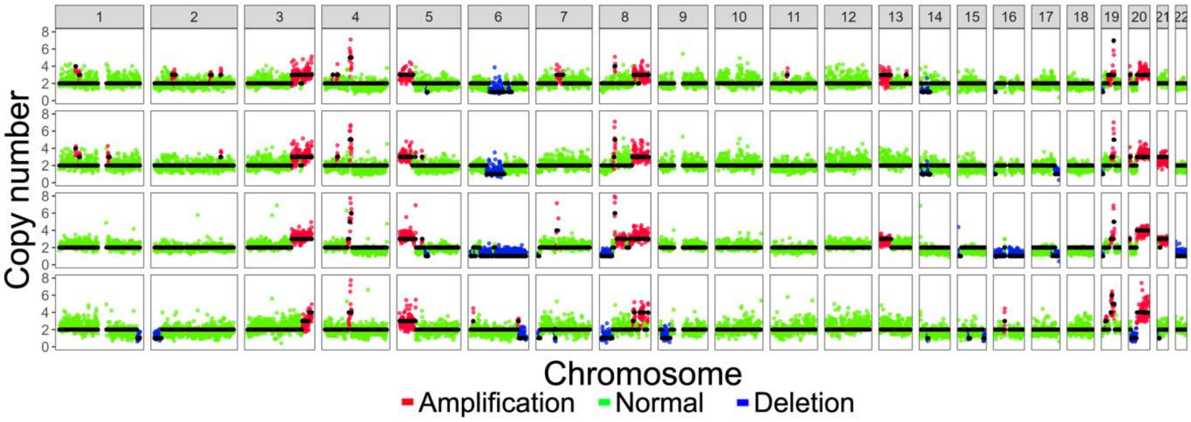

Supplementary Figure 22. Single-cell CNA profiles of 4 single HK2-derived CTCs across the genome from P15 (ovarian cancer). The ascites cytology showed an atypical result. Single-cell genome sequencing provides compelling evidence of cell malignancy that generates a MA diagnosis.

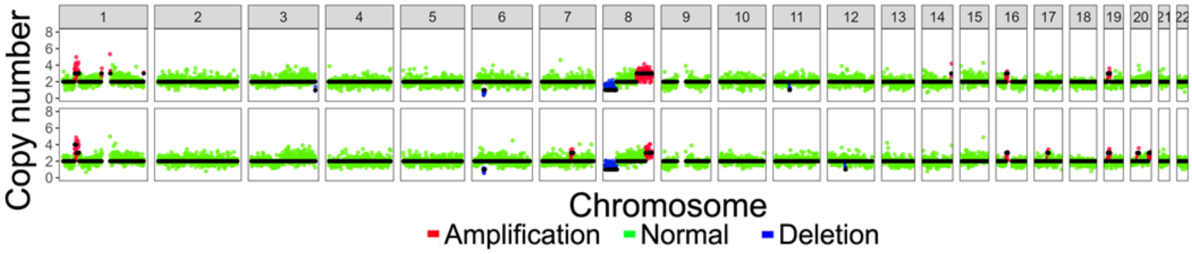

Supplementary Figure 23. Single-cell CNA profiles of 2 single HK2-derived CTCs across the genome from P18 (ovarian cancer).

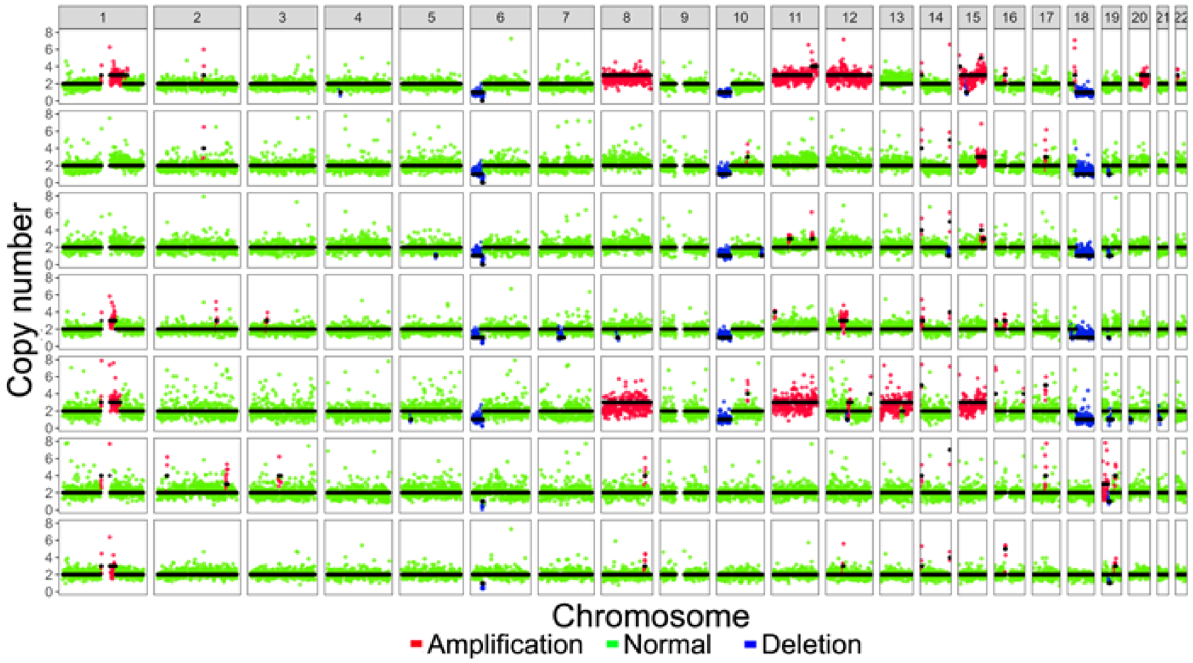

Supplementary Figure 24. Single-cell CNA profiles of 4 single HK2-derived CTCs (top four) and 3 CTC clusters (last three) across the genome from P22 (cervical cancer). The ascites cytology showed an atypical result. Single-cell genome sequencing provides compelling evidence of cell malignancy that generates a MA diagnosis.

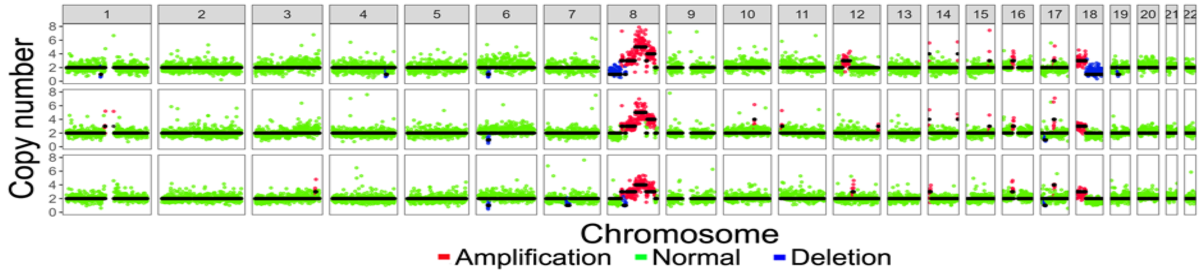

Supplementary Figure 25. Single-cell CNA profiles of 3 single HK2-derived CTCs across the genome from P24 (endometrial cancer).

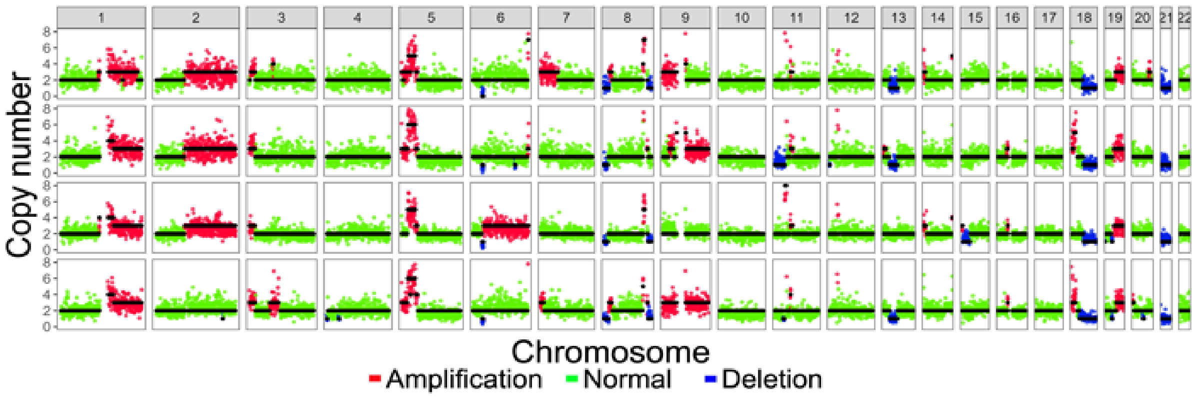

Supplementary Figure 26. Single-cell CNA profiles of 4 single HK2-derived CTCs across the genome from P26 (colorectal cancer).

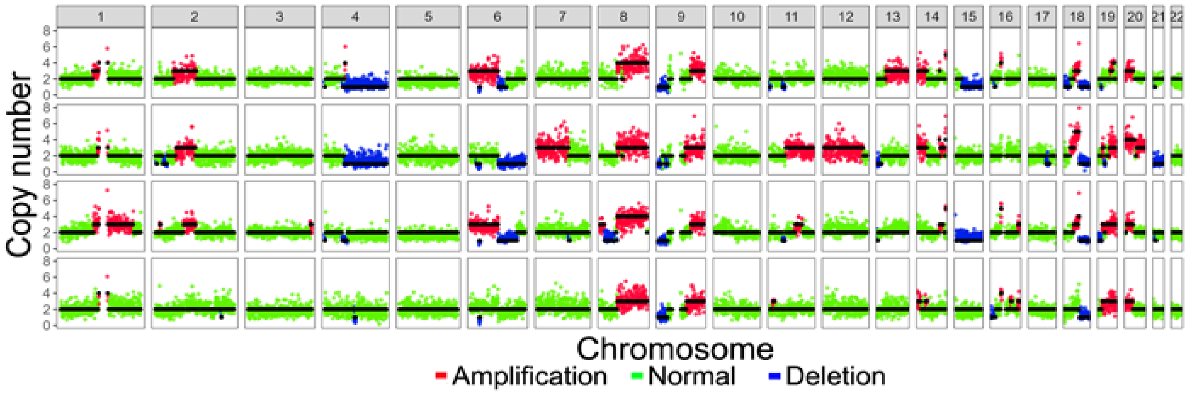

Supplementary Figure 27. Single-cell CNA profiles of 4 single HK2-derived CTCs across the genome from P34 (small intestine cancer). The ascites cytology showed a negative result. Single-cell genome sequencing provides compelling evidence of cell malignancy that generates a MA diagnosis.

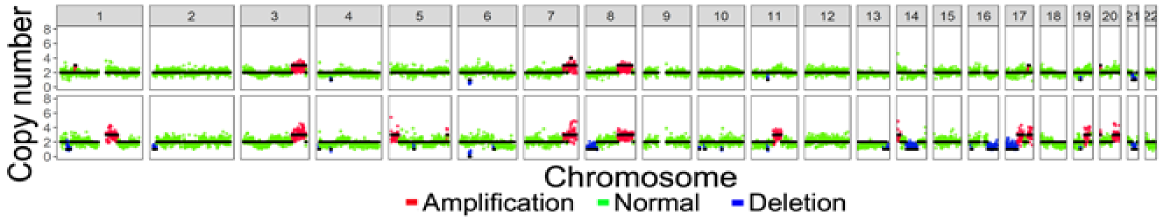

Supplementary Figure 28. Single-cell CNA profiles of 2 single HK2-derived CTCs across the genome from P35 (gastric cancer). The ascites cytology showed a negative result. Single-cell genome sequencing provides compelling evidence of cell malignancy that generates a MA diagnosis.

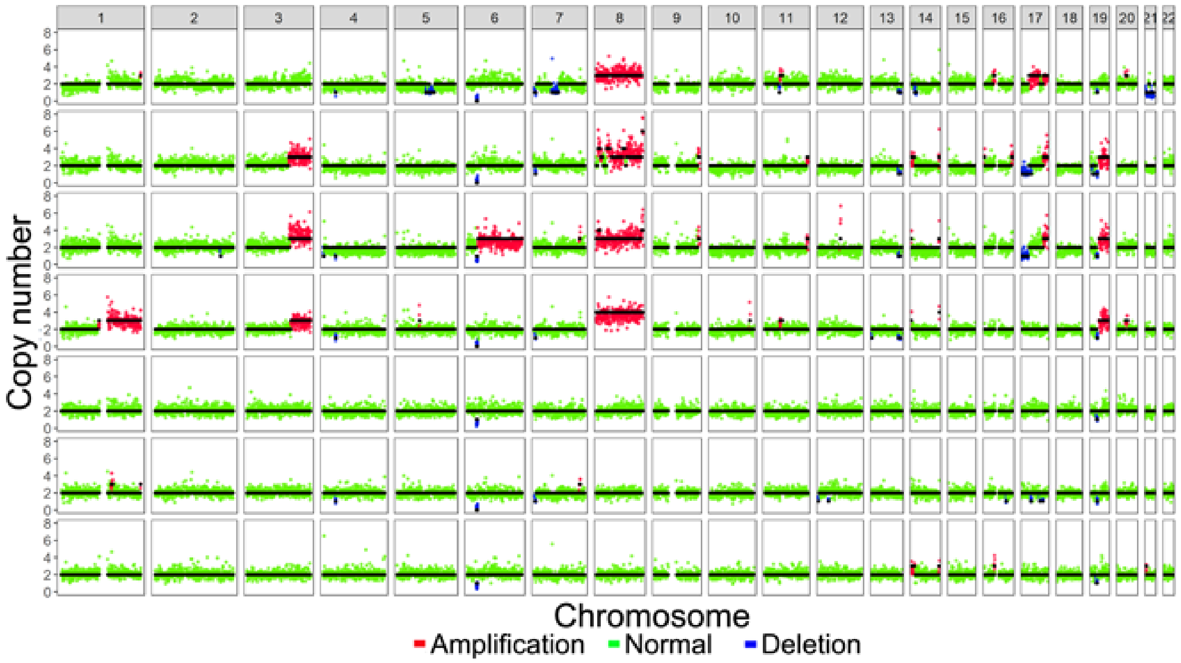

Supplementary Figure 29. Single-cell CNA profiles of 7 single HK2-derived CTCs across the genome from P36 (gastric cancer).

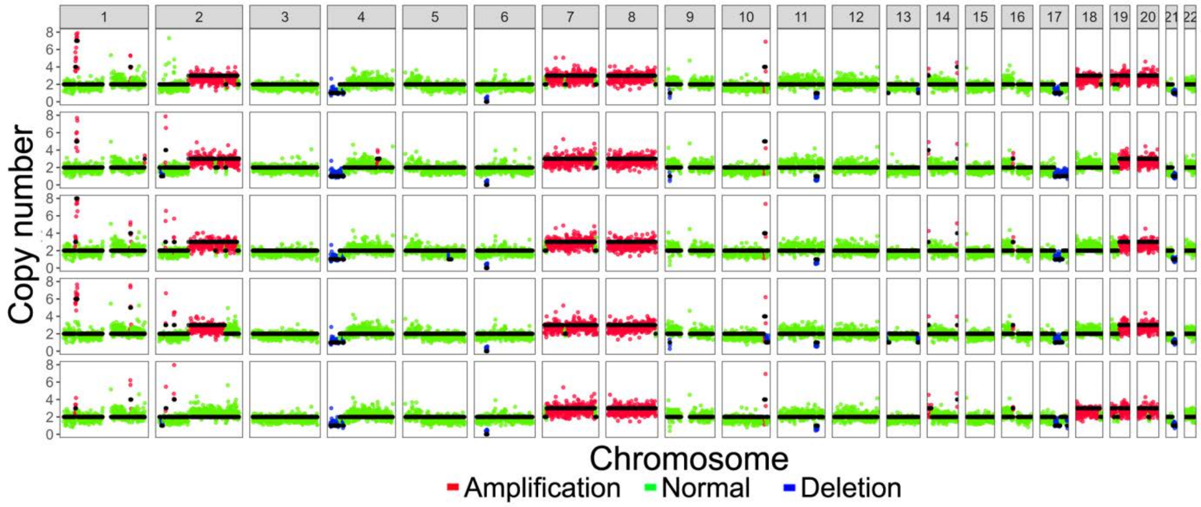

Supplementary Figure 30. Single-cell CNA profiles of 5 single HK2-derived CTCs across the genome from P37 (gastric cancer).

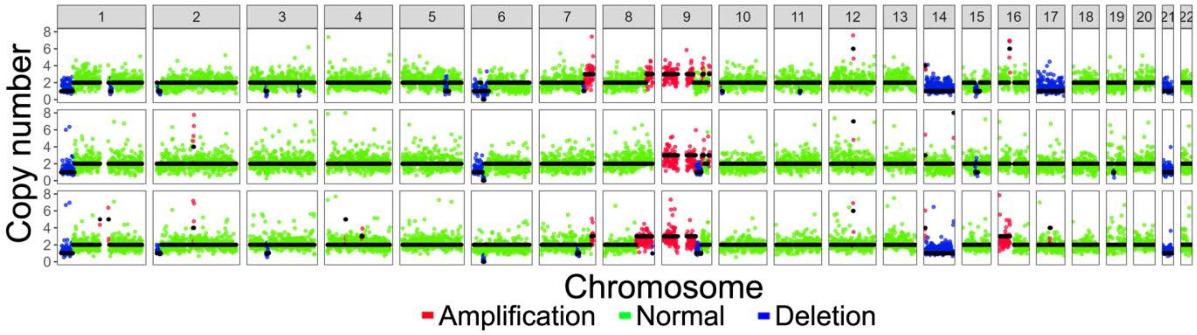

Supplementary Figure 31. Single-cell CNA profiles of 3 single HK2-derived CTCs across the genome from P38 (gastric cancer). The ascites cytology showed an atypical result. Single-cell genome sequencing provides compelling evidence of cell malignancy that generates a MA diagnosis.

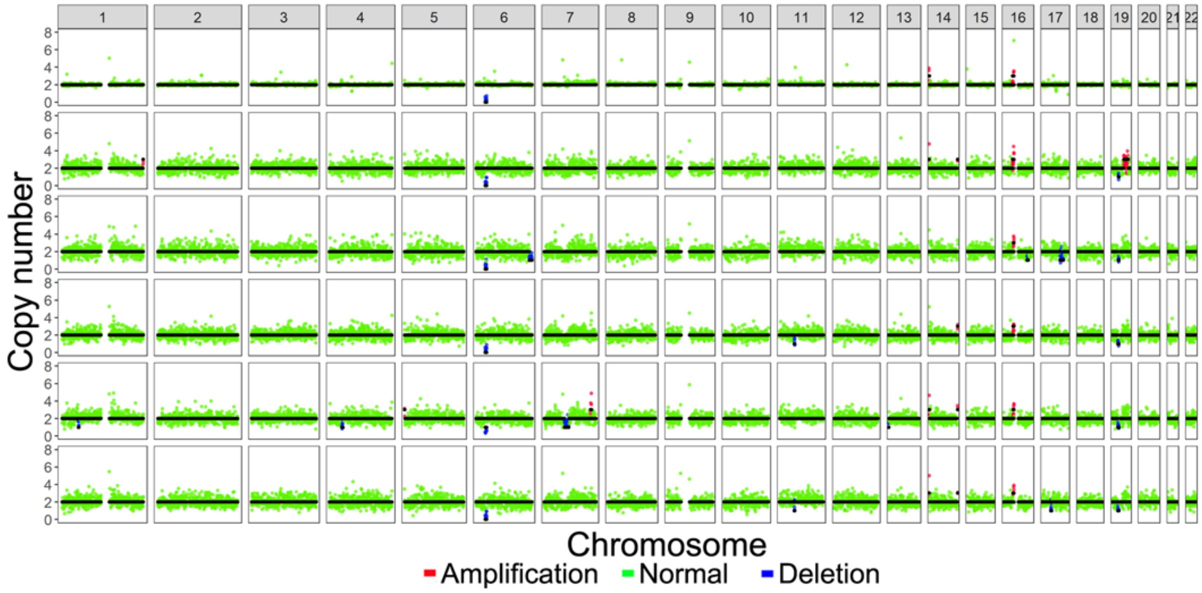

Supplementary Figure 32. Single-cell CNA profiles of primary tumor tissue (top), 3 single HK2-derived CTCs, and two 3-CTC pools (bottom two) across the genome from P39 (gastric cancer). The ascites cytology showed a positive result. The scMet-Seq shows negative results because CNA burden of sCTCs is smaller than threshold. However, CNA profiles of sCTCs are consistent with that of primary tissues. Tumors that are absent of detectable CNAs generate false negative of scMet-Seq.

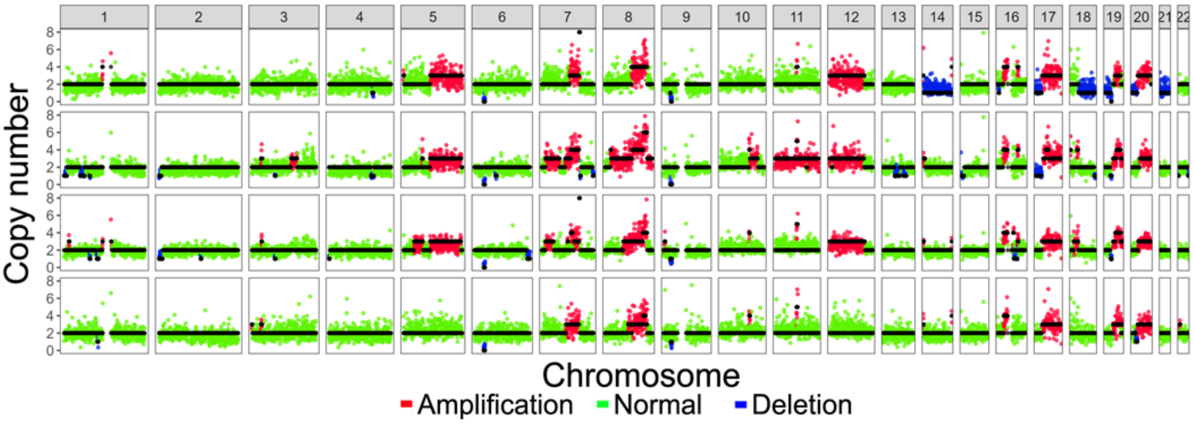

Supplementary Figure 33. Single-cell CNA profiles of 4 single HK2-derived CTCs across the genome from P40 (gastric cancer).

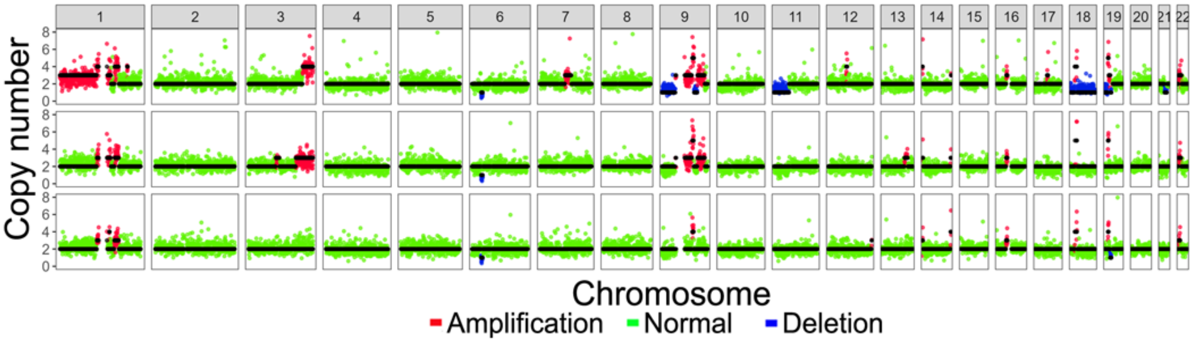

Supplementary Figure 34. Single-cell CNA profiles of 3 single HK2-derived CTCs across the genome from P42 (gastric cancer).

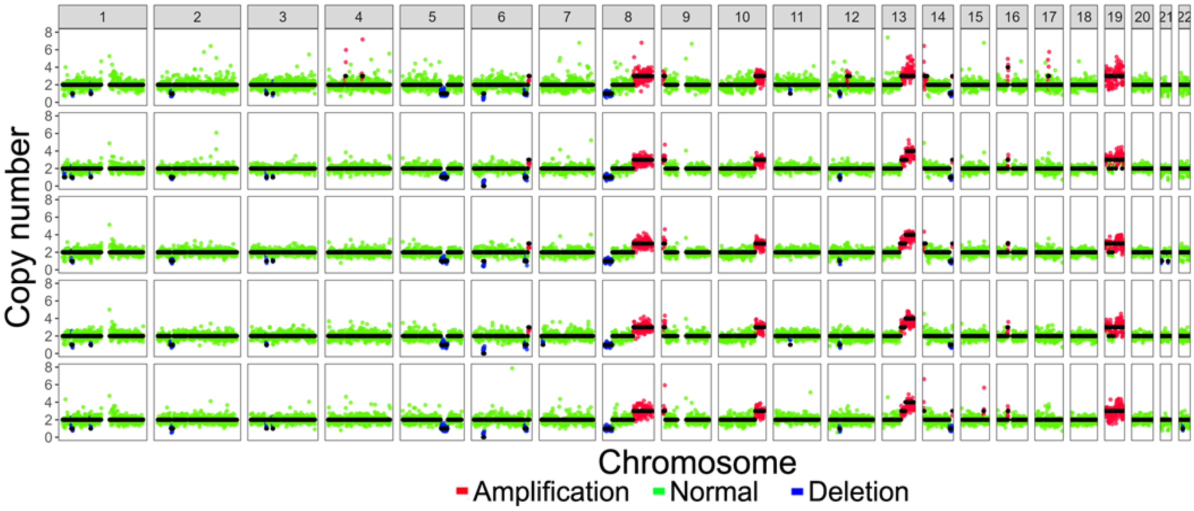

Supplementary Figure 35. Single-cell CNA profiles of 5 single HK2-derived CTCs across the genome from P43 (gastric cancer).

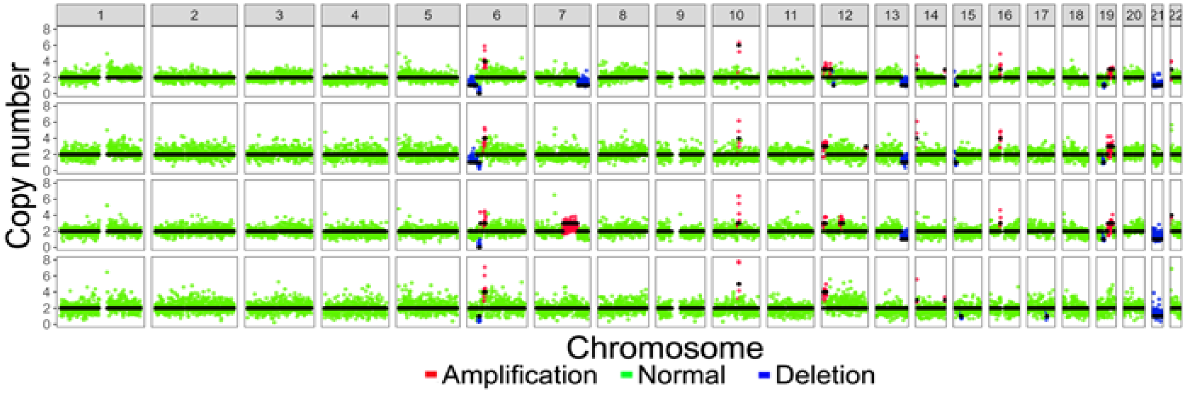

Supplementary Figure 36. Single-cell CNA profiles of 4 single HK2-derived CTCs across the genome from P45 (gastric cancer). The ascites cytology showed an atypical result. Single-cell genome sequencing provides compelling evidence of cell malignancy that generates a MA diagnosis.

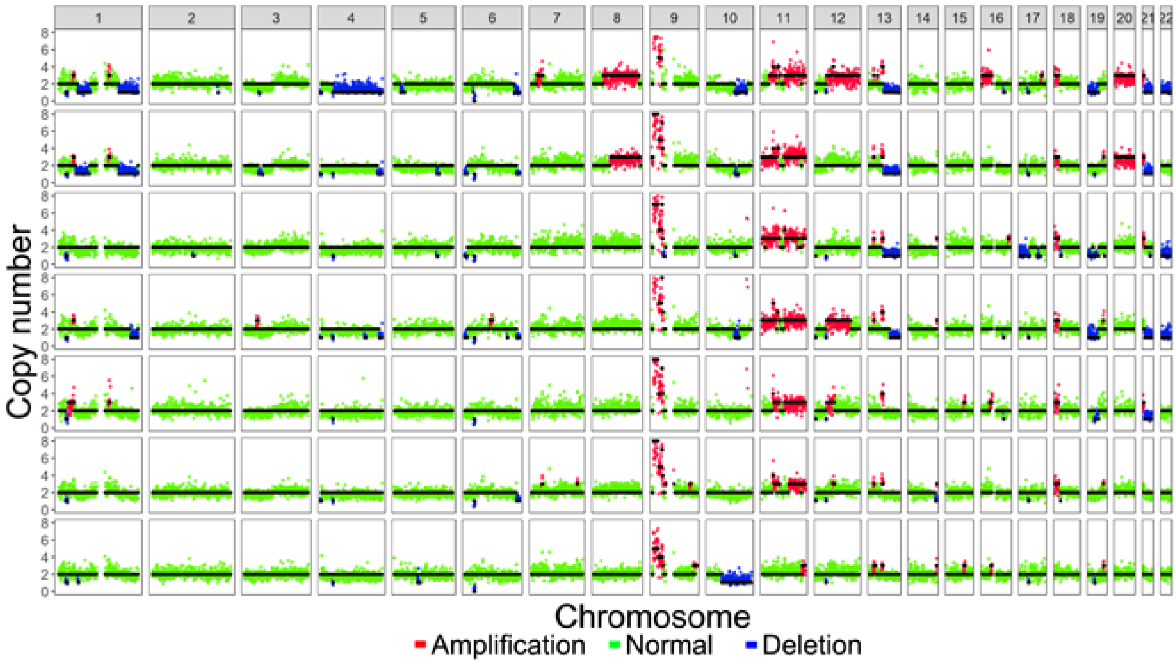

Supplementary Figure 37. Single-cell CNA profiles of 7 single HK2-derived CTCs across the genome from P48 (esophagogastric junction carcinoma).

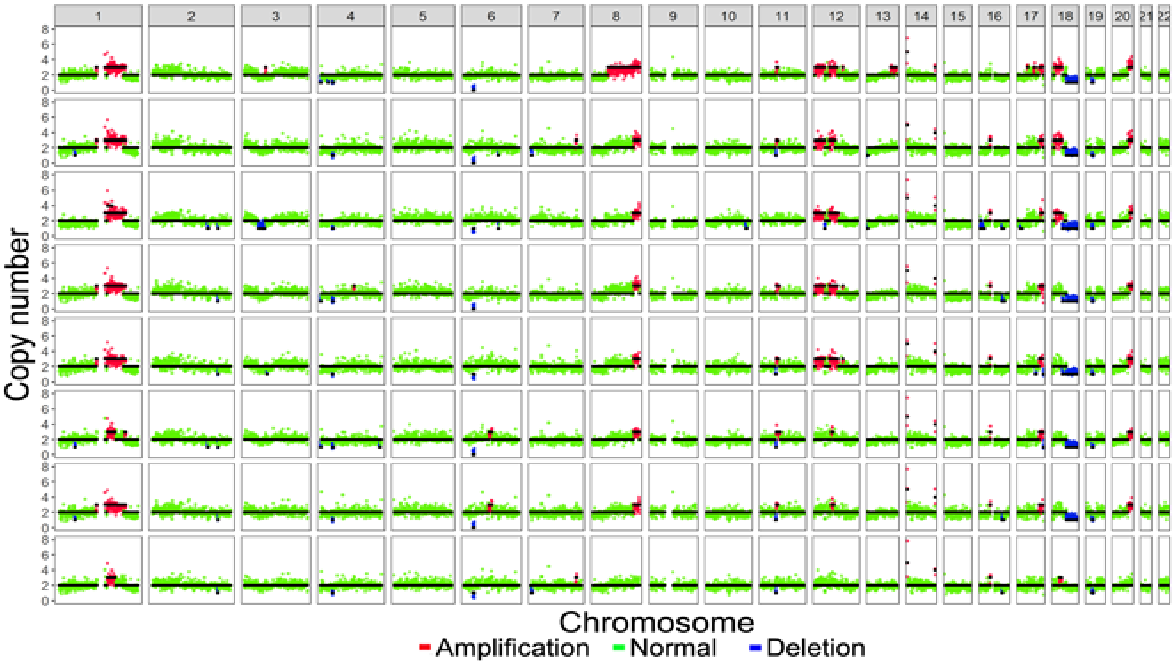

Supplementary Figure 38. Single-cell CNA profiles of 8 single HK2-derived CTCs across the genome from P49 (pancreatic cancer).

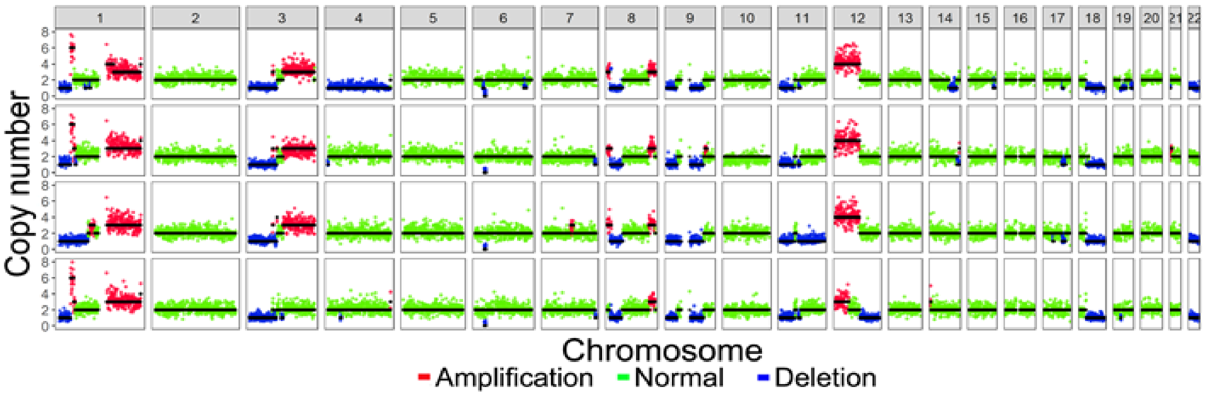

Supplementary Figure 39. Single-cell CNA profiles of 4 single HK2-derived CTCs across the genome from P51 (pancreatic cancer).

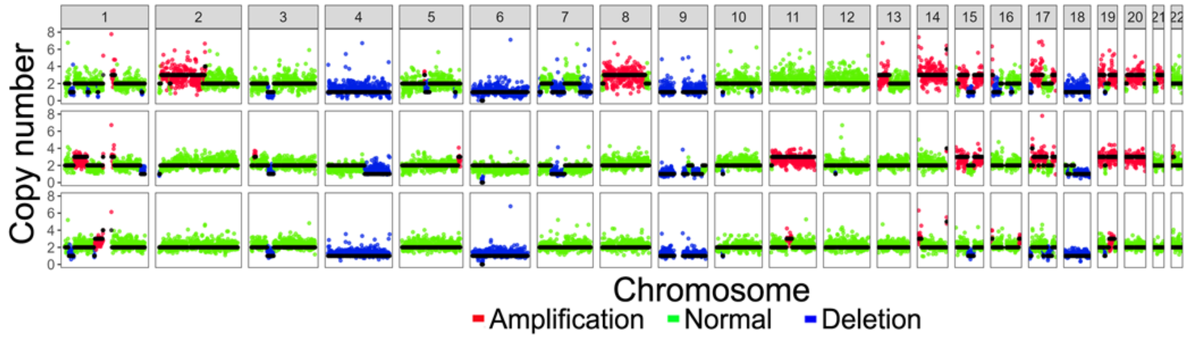

Supplementary Figure 40. Single-cell CNA profiles of 3 single HK2-derived CTCs across the genome from P52 (pancreatic cancer).

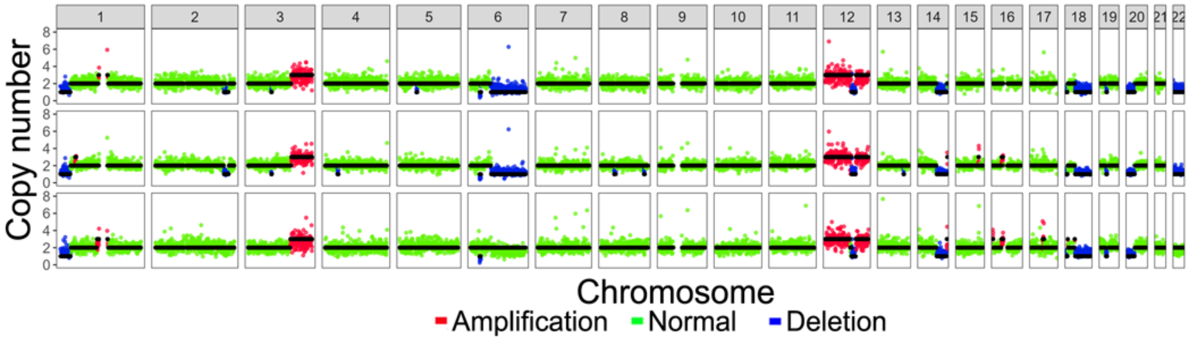

Supplementary Figure 41. Single-cell CNA profiles of 3 single HK2-derived CTCs across the genome from P54 (hepatocellular carcinoma). The ascites cytology showed a negative result. Single-cell genome sequencing provides compelling evidence of cell malignancy that generates a MA diagnosis.

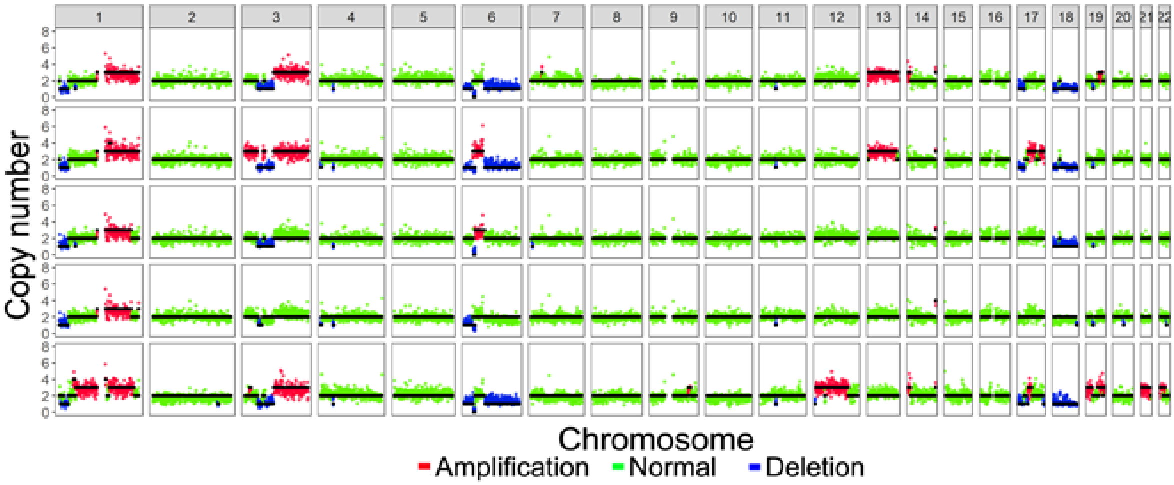

Supplementary Figure 42. Single-cell CNA profiles of 5 single HK2-derived CTCs across the genome from P56 (hepatocellular carcinoma). The ascites cytology showed a negative result. Single-cell genome sequencing provides compelling evidence of cell malignancy that generates a MA diagnosis.

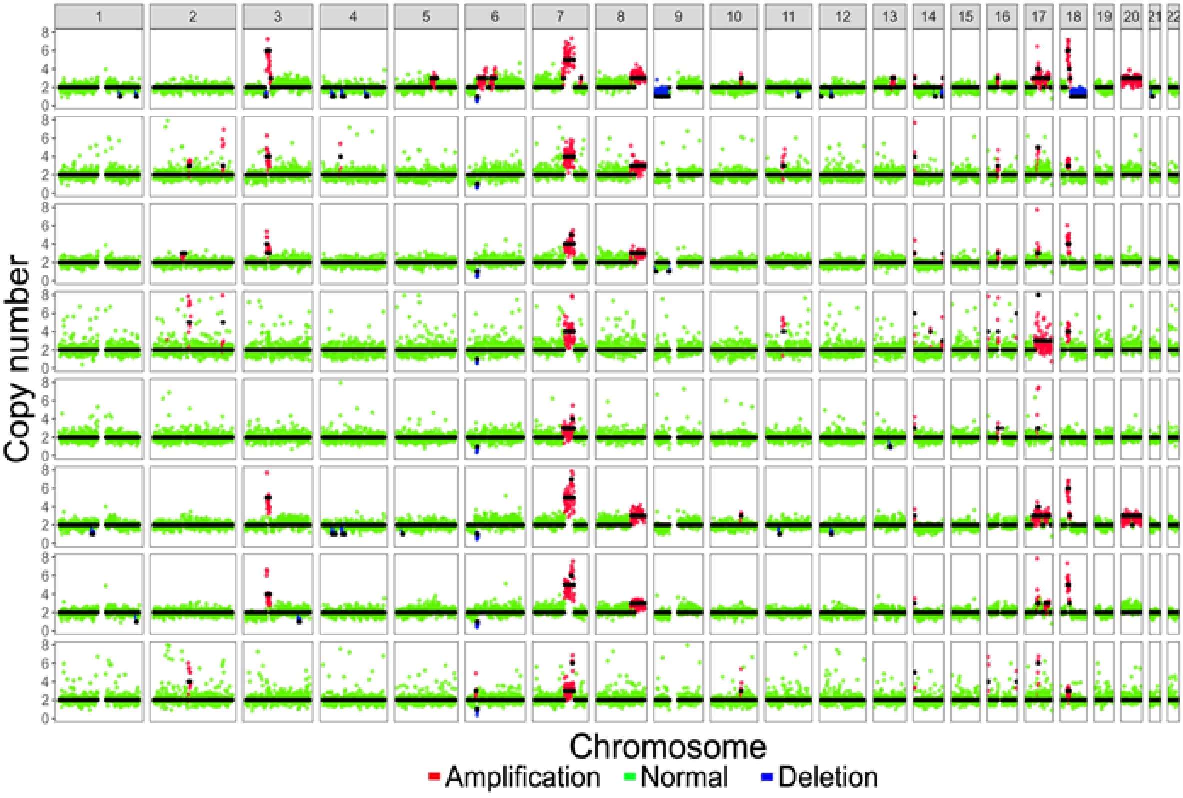

Supplementary Figure 43. Single-cell CNA profiles of 5 single HK2-derived CTCs and 3 CTC clusters (last three) across the genome from P61 (gallbladder cancer).

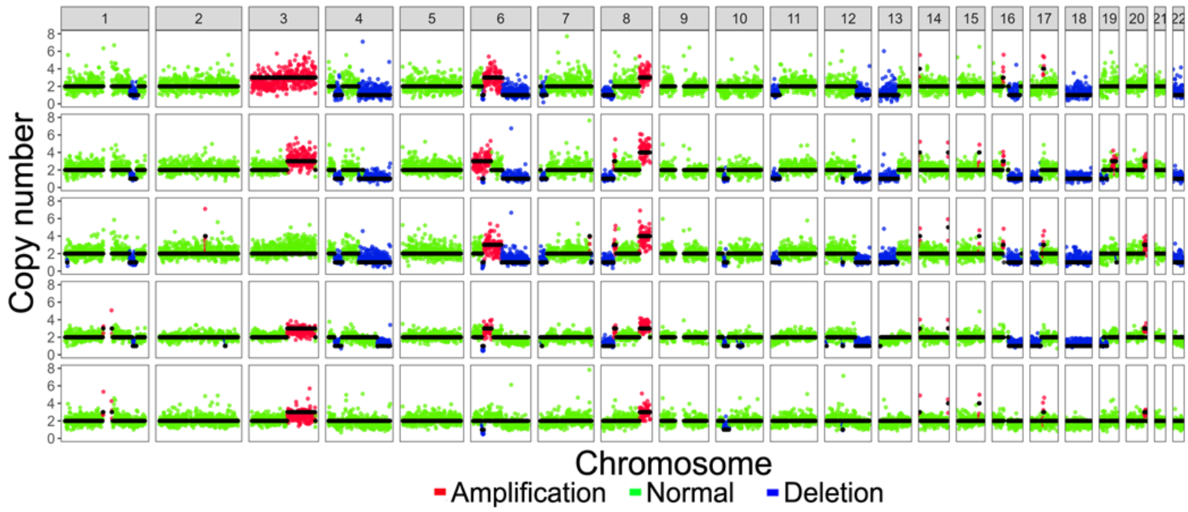

Supplementary Figure 44. Single-cell CNA profiles of 5 single HK2-derived CTCs across the genome from P62 (breast cancer).

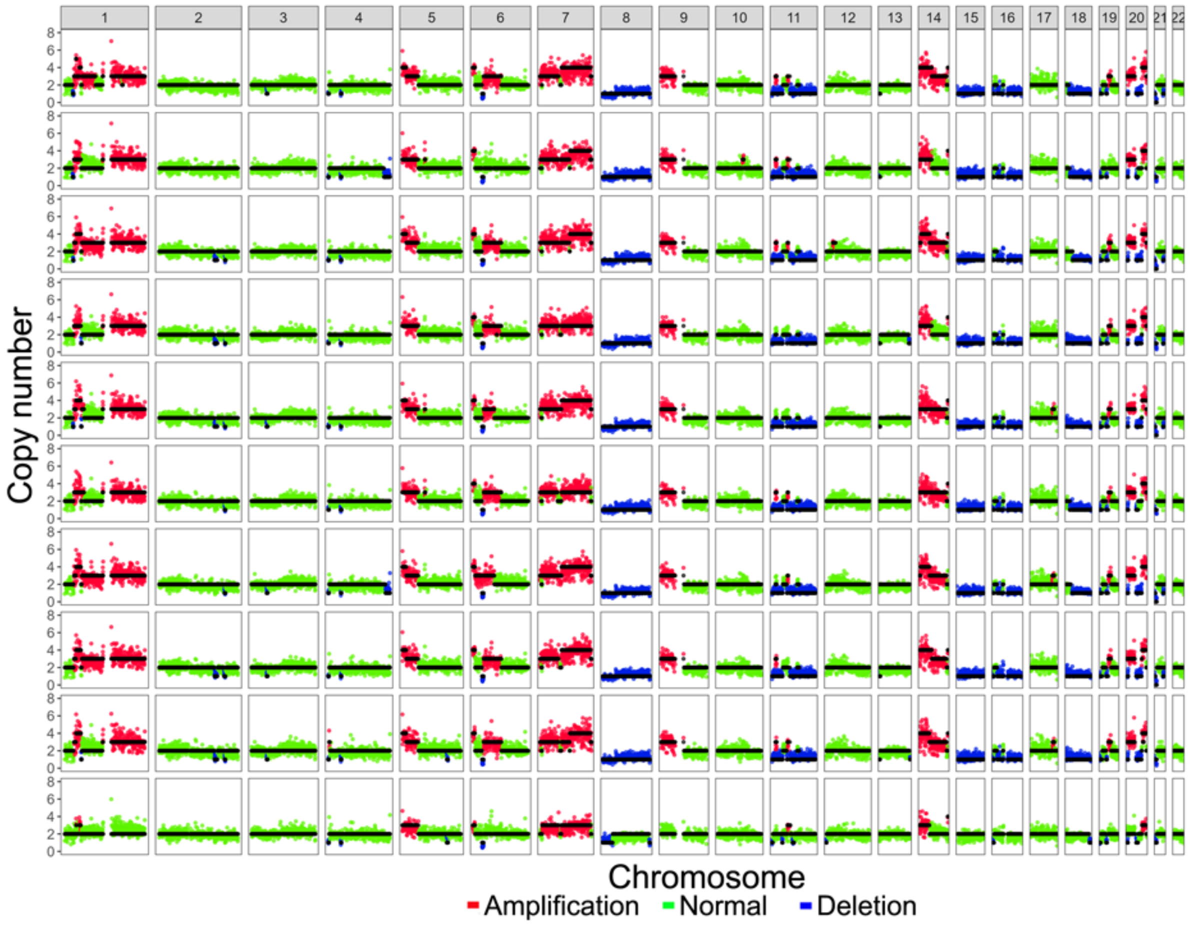

Supplementary Figure 45. Single-cell CNA profiles of 10 single HK2-derived CTCs across the genome from P64 (lung cancer)

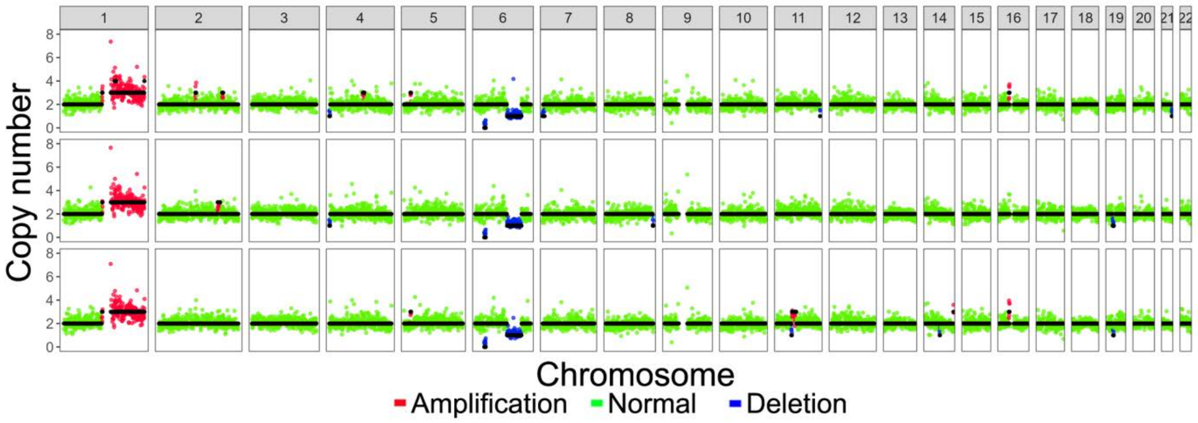

Supplementary Figure 46. Single-cell CNA profiles of 3 single HK2-derived CTCs across the genome from P68 (lymphoma).

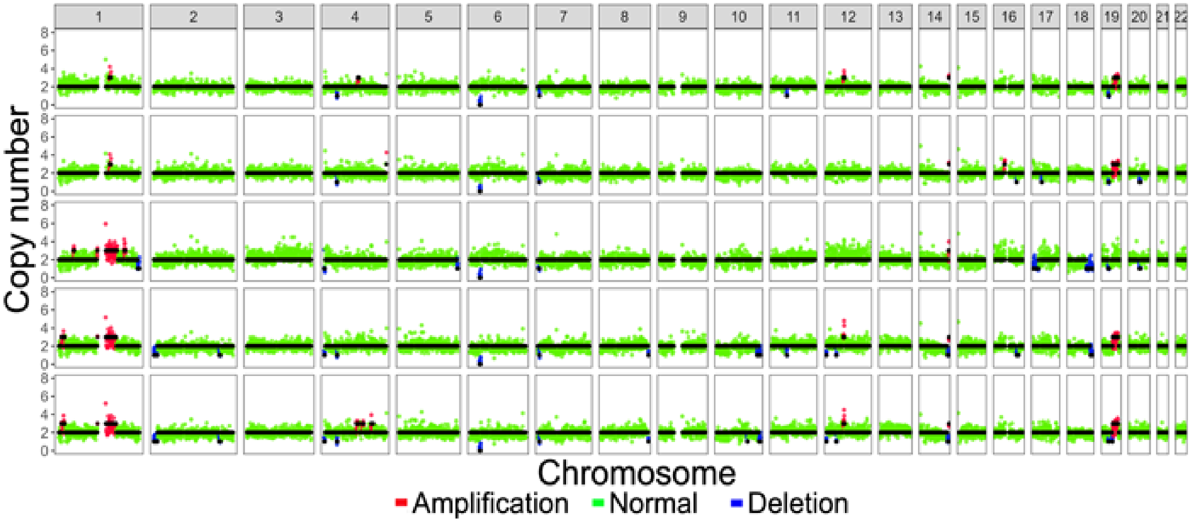

Supplementary Figure 47. Single-cell CNA profiles of 5 single HK2-derived CTCs across the genome from P70 (cancer of unknown primary). The ascites cytology showed a negative result. Single-cell sequencing provides compelling evidence of cell malignancy that generates a MA diagnosis.

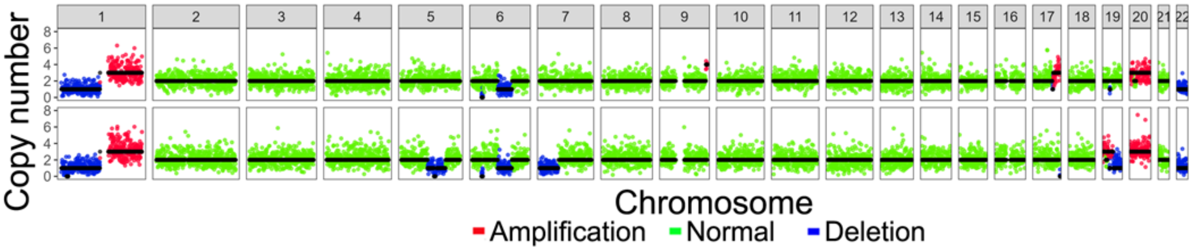

Supplementary Figure 48. Single-cell CNA profiles of 2 single HK2-derived CTCs across the genome from P71 (cancer of unknown primary). The ascites cytology showed anatypical result. Single-cell genome sequencing provides compelling evidence of cell malignancy that generates a MA diagnosis.

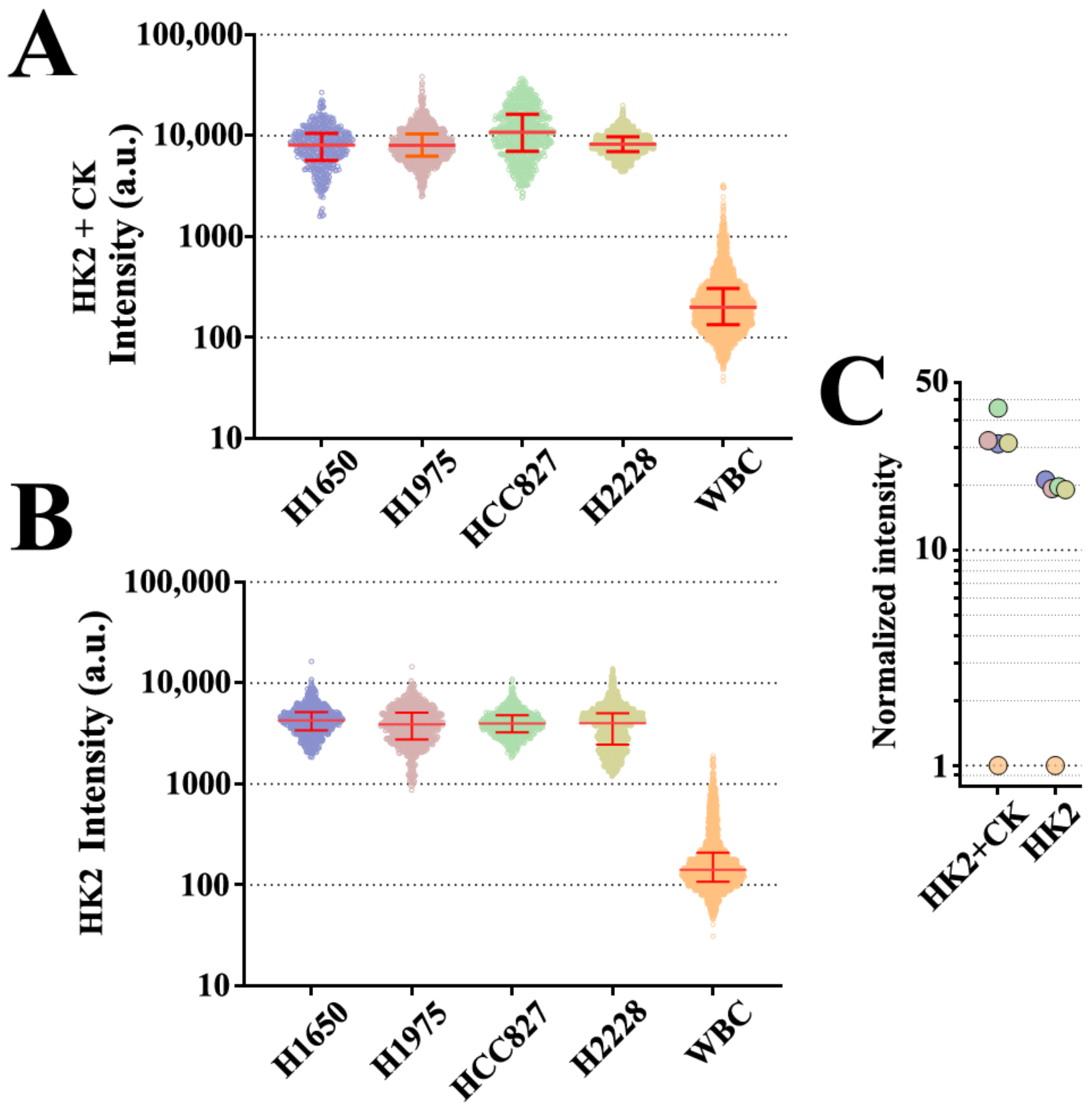

Supplementary Figure 49. (A) Fluorescence intensity of HK2 + CK level in lung cancer cell lines (H1650, H1975, HCC827 and H2228) and white blood cells (WBCs) from a healthy donor as the negative control; (B) Fluorescence intensity of HK2 level in lung cancer cell lines (H1650, H1975, HCC827 and H2228) and WBCs. (C) Normalized intensities of HK2 + CK level and HK2 level in lung cancer cells as normalized to the intensities of WBCs. Obviously, HK2 and CK combined in the same fluorescence channel significantly enhanced signals of cancer cell lines than HK2 alone, as compared to negative controls.

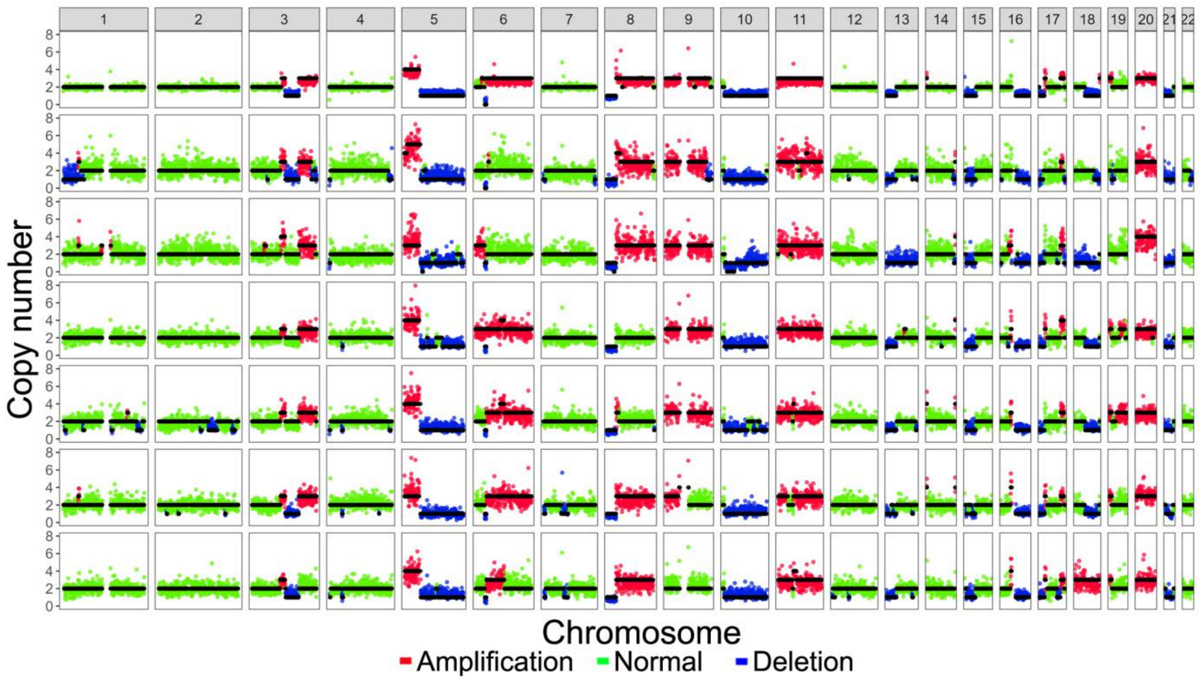

Supplementary Figure 50. CNA profiles of 6 single CTCs and the paired tumor tissue (top) across the genome from LC4.

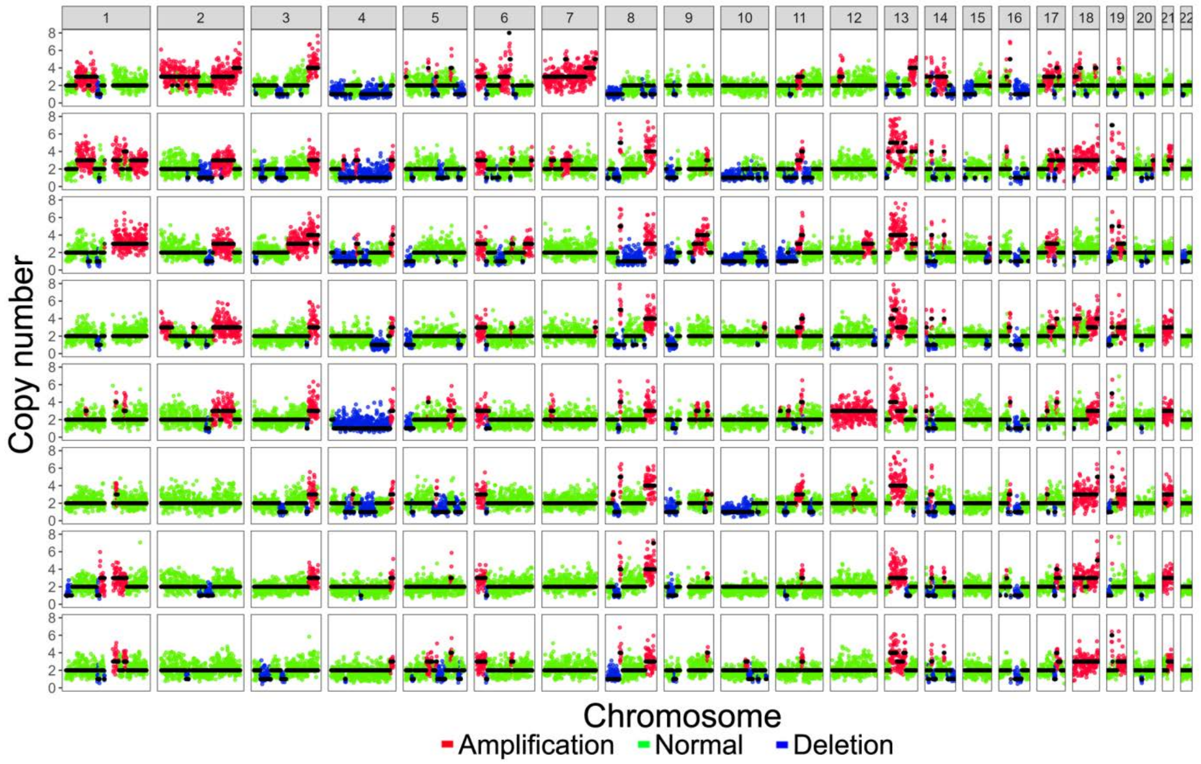

Supplementary Figure 51. Single-cell CNA profiles of 8 single CTCs across the genome from LC5.

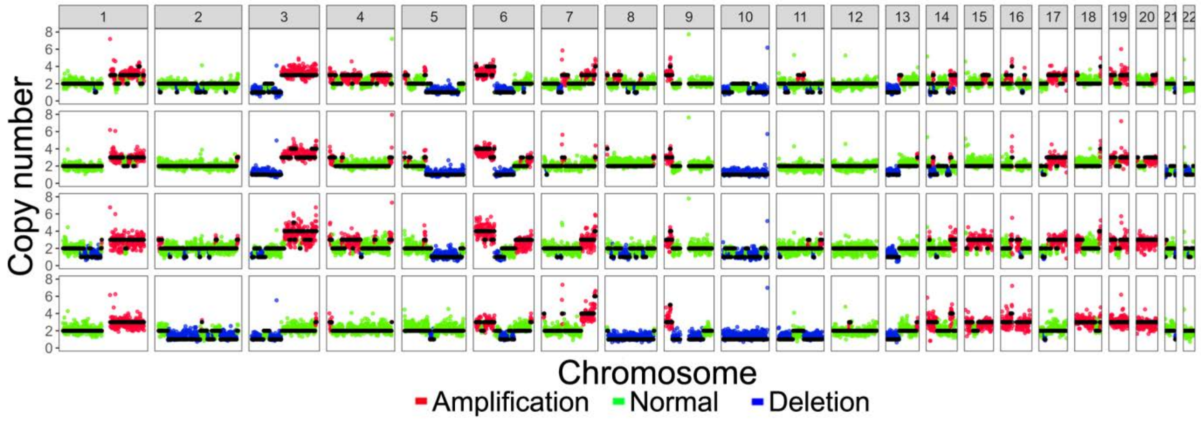

Supplementary Figure 52. Single-cell CNA profiles of 4 single CTCs across the genome from LC6.

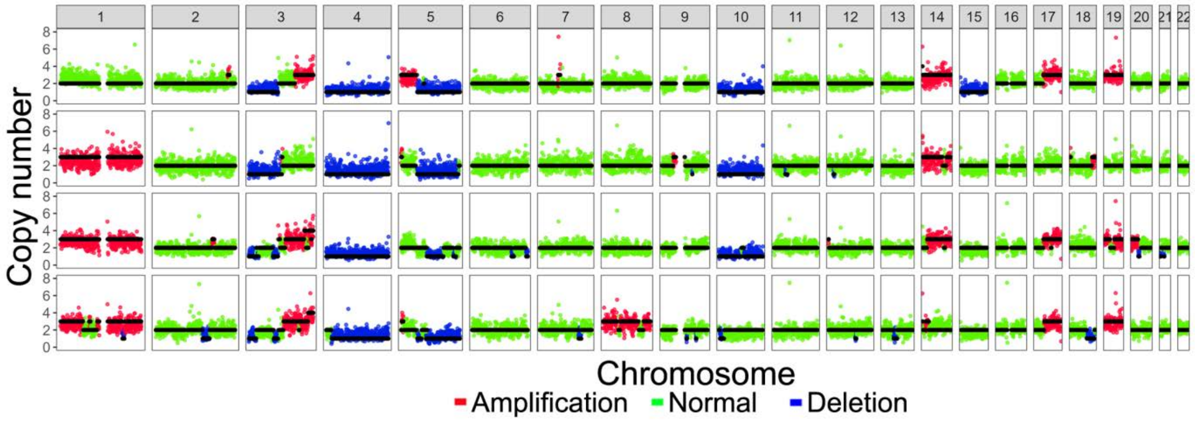

Supplementary Figure 53. Single-cell CNA profiles of 4 single CTCs across the genome from LC9.

Supplementary Tables

Supplementary Table 1. Primers used in this study for targeting 22 loci on different chromosomes. All primers were synthesized by Genewiz (Suzhou, China).

**Name**	**Sequence**	**Name**	**Sequence**
Chr1F	TTTAGGCGTCATCTGAGGGTA	Chr12F	ATCATGGAAATGCAGCCTCT
Chr1R	TGGCAGCAGTATGGAGAATGTA	Chr12R	AGAACCCAGCTGGAATGATG
Chr2F	AGCGGGAGGGACTATTTCAC	Chr13F	TGTTTCATGGAGTCCTGCTG
Chr2R	GGATCGTTCAAAGGGAACTG	Chr13R	GGAGGCAAGAACCAAACAAA
Chr3F	CCCTTGTACTGGCTCGTGTT	Chr14F	AGCCAAGACGTACCCTCTCA
Chr3R	CTTGCACATGAAGGTCTGGA	Chr14R	TGCTTTACACCAATCCCACA
Chr4F	GAGCATCTCTTGGCTCTGCT	Chr15F	TCAGCATGGGTTATGGGTTT
Chr4R	TTGGGAAAGCACAGATCCTT	Chr15R	CCCAGATGATGGAGAGGAAA
Chr5F	ACGGACAGTGGACAGATTGC	Chr16F	GCCTGTGTTTGCTGATGAAA
Chr5R	CCACTGTGCCACCCCATT	Chr16R	GGGCAACGACCGTACTTAAA
Chr6F	GAGGAGGGCAAGGAGAGAGT	Chr17F	TCCTGGGCTAGCCTTTTACA
Chr6R	ACCCTCCAGTGTGCAAAAAC	Chr17R	ATCGCTTGAGCACTGAAGGT
Chr7F	CTTCCTGCCATTCCACAAGT	Chr18F	AGACGAGCCTTTCTCTGTCG
Chr7R	CCCACTTTCATGCCTCTGAT	Chr18R	TCGAGACCATCCCCACTAAC
Chr8F	CTTCCCTGCCTTGCTCTCTA	Chr19F	AGTTGAGGAGATGGTGGAGC
Chr8R	CGGGACATTTCAGCAATCTT	Chr19R	AACAGGAGCCTTGGTCAGTC
Chr9F	CTGTGGAGCAGCTGTTTCTG	Chr20F	CTGGTCAAACATCTCCCTCGT
Chr9R	GAATTCACAAAGCCCCAAGA	Chr20R	CTCCACGCATCTTACATCACCT
Chr10F	CCCCTCATTCAAATCAGCAT	Chr21F	GGACTTTGCTGACGGGATTA
Chr10R	CAGGCAAAAGCTGGAGTTTC	Chr21R	GAACTAACGACCTCACGCTTG
Chr11F	TGAATGAGAACGCAGATGTGA	Chr22F	TCCACAACCCCTTATCTTACCC
Chr11R	CACAAAGCATCCAGGGTCATT	Chr22R	ACCTCAGGTGATCTACCCGC

Supplementary Table 2. Clinicopathological characteristics and HK2-derived sCTC numbers of patients with malignant ascites (MA) in the training cohort. A MA diagnosis is defined as: 1) positive cytology; or 2) positive peritoneal biopsy; or 3) hispathological confirmation of primary tumor and the patient had clinical or radiographic evidence of metastatic disease and had no alternative cause for the ascites. The sCTC counts lower than the threshold are shown in red.

No	Sex	Age	Cancer type	Stage	Metastasis	Treatment	Cytology	sCTC# / ml
T1	F	47	ovarian cancer	IV	Y	N	Positive	33.4
T2	F	72	ovarian cancer	IV	Y	N	Positive	206.0
T3	F	40	ovarian cancer	IV	Y	N	Positive	5.8
T4	F	59	ovarian cancer	IV	Y	N	Positive	1.8
T5	F	72	ovarian cancer	IV	Y	N	Positive	31.0
T6	F	70	endometrial cancer	III	N	Y	Positive	10.0
T7	F	46	endometrial cancer	IV	Y	N	Positive	98.7
T8	F	64	colorectal cancer	IV	Y	Y	Positive	2.5
T9	F	50	cecum cancer	IV	Y	Y	Positive	8.4
T10	F	59	gastric cancer	IV	Y	Y	Positive	5.7
T11	F	69	gastric cancer	IV	Y	Y	Positive	9.2
T12	M	51	gastric cancer	IV	Y	Y	Positive	5.9
T13	F	76	pancreatic cancer	IV	Y	N	Positive	2.1
T14	M	71	pancreatic cancer	IV	Y	N	Atypical	360.0
T15	F	78	gallbladder cancer	IV	Y	Y	Positive	0.5
T16	M	67	lung cancer	IV	Y	Y	Positive	32.0
T17	F	48	lung cancer	IV	Y	Y	Positive	304.0
T18	M	39	lymphoma	IV	Y	N	Positive	3.8
T19	M	66	leiomyosarcoma	IV	Y	Y	Negative	24.5
T20	F	71	primary peritoneal carcinoma	II	N	N	Positive	44.0

Supplementary Table 3. Clinicopathological characteristics and HK2-derived sCTC numbers of patients with benign ascites (BA) in the training cohort. A BA diagnosis is defined as: 1) the patient had no evidence of malignancy; and 2) a strong etiology of benign disease could explain the ascites. The sCTC counts greater than the threshold are shown in red.

No	Sex	Age	Etiology	Cytology	sCTC# / ml
T21	M	89	hepatic diseases	Negative	0.0
T22	M	68	hepatic diseases	Negative	9.7
T23	M	79	hepatic diseases	negative	1.6
T24	F	75	hepatic diseases	negative	5.8
T25	M	59	hepatic diseases	Negative	2.2
T26	M	57	hepatic diseases	Negative	0.6
T27	M	63	hepatic diseases	Negative	0.0
T28	F	56	hepatic diseases	Negative	0.0
T29	M	71	hepatic diseases	Negative	0.8
T30	F	52	inflammation	Negative	0.0
T31	M	34	inflammation	Negative	0.0
T32	F	71	inflammation	Negative	0.4
T33	F	94	inflammation	negative	0.0
T34	M	65	inflammation	Negative	0.0
T35	M	58	inflammation	Negative	0.0
T36	F	64	others	Negative	0.2
T37	F	39	others	Negative	1.5
T38	F	55	others	Negative	0.0
T39	F	68	others	Negative	0.0
T40	F	48	others	Negative	0.0

Supplementary Table 4. Clinicopathological characteristics, HK2-derived sCTC counts and scMet-Seq results of patients with malignant ascites (MA) in the validation cohort. False negative results of the scMet-Seq are shown in red.

No	Sex	Age	Cancer type	Stage	Metastasis	Treatment	Cytology	sCTC# /ml	sCTC# > cut-off	scMet-Seq
P1	F	65	ovarian cancer	IV	Y	N	Positive	14.6	Y	Positive
P2	F	86	ovarian cancer	IV	Y	N	Positive	42.5	Y	Positive
P3	F	57	ovarian cancer	IV	Y	N	Positive	6.8	Y	Positive
P4	F	77	ovarian cancer	II	N	Y	Positive	6.0	Y	Positive
P5	F	67	ovarian cancer	IV	Y	N	Positive	2.2	Y	Positive
P6	F	78	ovarian cancer	IV	Y	N	Positive	11.5	Y	Positive
P7	F	52	ovarian cancer	IV	Y	N	Positive	8.2	Y	Positive
P8	F	53	ovarian cancer	IV	Y	N	Positive	46.8	Y	Positive
P9	F	67	ovarian cancer	IV	Y	N	Positive	6.3	Y	Positive
P10	F	57	ovarian cancer	IV	Y	Y	Positive	12.2	Y	Positive
P11	F	56	ovarian cancer	IV	Y	Y	Negative	4.1	Y	Positive
P12	F	67	ovarian cancer	IV	Y	Y	Positive	5.3	Y	Positive
P13	F	50	ovarian cancer	IV	Y	N	Negative	12.0	Y	Positive
P14	F	52	ovarian cancer	IV	Y	Y	Positive	5.2	Y	Positive
P15	F	48	ovarian cancer	IV	Y	N	Atypical	10.5	Y	Positive
P16	F	78	ovarian cancer	IV	Y	Y	Positive	4.4	Y	Positive
P17	F	61	ovarian cancer	IV	Y	N	Positive	39.0	Y	Positive
P18	F	62	ovarian cancer	IV	Y	N	Positive	34.0	Y	Positive
P19	F	63	ovarian cancer	IV	Y	N	Positive	17.8	Y	Positive
P20	F	58	ovarian cancer	II	N	N	Positive	15.2	Y	Positive
P21	F	64	ovarian clear cell carcinoma	I	N	N	Positive	5.2	Y	Negative
P22	F	67	cervical cancer	I	N	Y	Atypical	6.8	Y	Positive

P23	F	61	cervical cancer	II	N	Y	Negative	2.4	Y	Positive
P24	F	53	endometrial cancer	III	N	Y	Positive	2.1	Y	Positive
P25	F	33	endometrial cancer	IV	Y	N	Negative	0.0	N	Negative
P26	M	62	colorectal cancer	IV	Y	Y	Positive	52.4	Y	Positive
P27	M	70	colorectal cancer	III	N	Y	Negative	10.6	Y	Positive
P28	F	74	colorectal cancer	IV	Y	N	Negative	3.0	Y	Positive
P29	M	59	colorectal cancer	IV	Y	Y	Negative	0.0	N	Negative
P30	M	30	colorectal cancer	IV	Y	Y	Positive	39.5	Y	Positive
P31	M	56	colorectal cancer	IV	Y	Y	Positive	4.2	Y	Positive
P32	M	69	colorectal cancer	IV	Y	Y	Negative	5.4	Y	Negative
P33	M	66	colorectal cancer	IV	Y	Y	Positive	10.0	Y	Positive
P34	M	59	small intestine cancer	IV	Y	Y	Negative	5.8	Y	Positive
P35	F	47	gastric cancer	IV	Y	Y	Negative	2.5	Y	Positive
P36	F	58	gastric cancer	IV	Y	N	Positive	2.3	Y	Positive
P37	M	82	gastric cancer	IV	Y	N	Positive	8.6	Y	Positive
P38	F	65	gastric cancer	IV	Y	Y	Atypical	7.0	Y	Positive
P39	F	55	gastric cancer	IV	Y	Y	Positive	2.4	Y	Negative
P40	M	56	gastric cancer	IV	Y	N	Positive	2.2	Y	Positive
P41	F	70	gastric cancer	IV	Y	Y	Negative	0.3	N	Negative
P42	F	51	gastric cancer	IV	Y	Y	Positive	6.0	Y	Positive
P43	F	55	gastric cancer	IV	Y	Y	Positive	14.6	Y	Positive
P44	F	56	gastric cancer	IV	Y	Y	Atypical	1.0	N	Negative
P45	F	68	gastric cancer	IV	Y	N	Atypical	9.6	Y	Positive
P46	F	53	gastric cancer	IV	Y	Y	Atypical	0.2	N	Negative
P47	M	59	esophageal cancer	IV	Y	Y	Negative	0.6	N	Negative

P48	M	65	esophagogastric junction carcinoma	IV	Y	N	Positive	2.4	Y	Positive
P49	F	58	pancreatic cancer	IV	Y	N	Positive	13.0	Y	Positive
P50	F	69	pancreatic cancer	IV	Y	N	Negative	0.2	N	Negative
P51	F	70	pancreatic cancer	IV	Y	N	Positive	2.2	Y	Positive
P52	M	61	pancreatic cancer	IV	Y	Y	Positive	10.9	Y	Positive
P53	M	68	pancreatic cancer	IV	Y	N	Negative	3.9	Y	Positive
P54	M	48	hepatocellular carcinoma	IV	Y	N	Negative	3.5	Y	Positive
P55	F	51	hepatocellular carcinoma	IV	Y	N	Negative	2.4	Y	Positive
P56	F	69	hepatocellular carcinoma	IV	Y	N	Negative	15.7	Y	Positive
P57	F	37	hepatocellular carcinoma	IV	Y	Y	Negative	2.6	Y	Negative
P58	M	88	cholangiocarcinoma	IV	Y	N	Negative	7.2	Y	Positive
P59	M	81	cholangiocarcinoma	IV	Y	N	Negative	4.9	Y	Positive
P60	F	57	cholangiocarcinoma	IV	Y	Y	Negative	5.8	Y	Positive
P61	F	65	gallbladder cancer	IV	Y	N	Positive	6.6	Y	Positive
P62	F	53	breast cancer	IV	Y	N	Positive	246.0	Y	Positive
P63	F	56	breast cancer	IV	Y	Y	Atypical	12.1	Y	Negative
P64	F	57	lung cancer	IV	Y	Y	Positive	2.5	Y	Positive
P65	M	71	nasopharyngeal cancer	IV	Y	Y	Negative	2.4	Y	Negative
P66	F	65	primary peritoneal mesothelioma	I	N	Y	Atypical	6.0	Y	Negative
P67	F	85	lymphoma	IV	Y	N	Atypical	40.6	Y	Positive
P68	M	75	lymphoma	IV	Y	Y	Positive	6.4	Y	Positive
P69	F	46	leiomyosarcoma	IV	Y	N	Negative	3.1	Y	Negative
P70	F	51	cancers of unknown primary	IV	Y	N	Negative	3.4	Y	Positive
P71	F	54	cancers of unknown primary	IV	Y	N	Atypical	2.8	Y	Positive

Supplementary Table 5. Clinicopathological characteristics, HK2-derived sCTC counts and scMet-Seq results of patients with benign ascites (BA) in the validation cohort. The sCTC counts greather than the threshold ares shown in red.

No	Sex	Age	Etiology	Cytology	sCTC# / ml	sCTC# > cut-off	scMet-Seq
B1	M	53	hepatic diseases	Negative	7.3	Y	Negative
B2	F	79	hepatic diseases	Negative	0.0	N	Negative
B3	F	86	hepatic diseases	Negative	0.7	N	Negative
B4	F	64	hepatic diseases	Negative	0.0	N	Negative
B5	M	71	hepatic diseases	Negative	0.0	N	Negative
B6	M	34	hepatic diseases	Negative	0.0	N	Negative
B7	F	82	hepatic diseases	Negative	6.7	Y	Negative
B8	M	83	hepatic diseases	Negative	0.0	N	Negative
B9	F	45	hepatic diseases	Negative	0.0	N	Negative
B10	M	75	hepatic diseases	Negative	0.1	N	Negative
B11	F	65	hepatic diseases	Negative	0.1	N	Negative
B12	F	50	hepatic diseases	Negative	0.6	N	Negative
B13	M	63	hepatic diseases	Negative	0.0	N	Negative
B14	M	57	hepatic diseases	Negative	0.6	N	Negative
B15	F	75	inflammation	Negative	0.1	N	Negative
B16	F	75	inflammation	Negative	0.1	N	Negative
B17	F	52	inflammation	Negative	2.1	Y	Negative
B18	F	64	inflammation	Negative	0.2	N	Negative
B19	M	47	inflammation	Negative	7.0	Y	Negative
B20	F	52	inflammation	Negative	0.0	N	Negative
B21	M	78	inflammation	Negative	0.4	N	Negative
B22	M	62	others	Negative	0.0	N	Negative
B23	F	24	others	Negative	0.0	N	Negative
B24	F	53	others	Negative	5.7	Y	Negative
B25	F	47	others	Negative	0.0	N	Negative
B26	F	25	others	Negative	0.2	N	Negative
B27	F	62	others	Negative	0.2	N	Negative
B28	M	62	others	Negative	0.0	N	Negative
B29	M	67	others	Negative	1.2	N	Negative
B30	F	70	others	Negative	0.0	N	Negative
B31	F	69	others	Negative	0.0	N	Negative
B32	F	55	others	Negative	0.0	N	Negative

Supplementary Table 6. Results of cytology, scMet-Seq and a combination of cytology and scMet-Seq for ascites diagnosis in the validation cohort. PPV, positive predicative value; NPV, negative predicative value.

	Cytology			scMet-SEq			Combined		
	MA	BA	Total	MA	BA	Total	MA	BA	Total
Positive	37	0	37	56	0	56	58	0	58
Negative	34	32	66	15	32	47	13	32	45
Total	71	32	103	71	32	103	71	32	103
Sensitivity	0.52 (95% CI: 0.40-0.64)			0.79 (95% CI: 0.69-0.88)			0.82 (95% CI: 0.73-0.91)		
Specificity	1 (95% CI: 1-1)			1 (95% CI: 1-1)			1 (95% CI: 1-1)		
PPV	1 (95% CI: 1-1)			1 (95% CI: 1-1)			1 (95% CI: 1-1)		
NPV	0.48 (95% CI: 0.36-0.61)			0.68 (95% CI: 0.55-0.81)			0.71 (95% CI: 0.58-0.84)		

Supplementary Table 7. Clinicopathological characteristics and sCTC counts of participants in the SCLC training cohort, including 26 treatment naïve SCLC patients (top) and 20 high-risk controls (bottom). Metastatic patients with sCTC counts lower than 3.0/ml are shown in red.

No	Age	Sex	TNM Stage	ED/LD stage	Distant metastasis	CTC#/ml
ED1	55	M	IV	Extensive	Bone, pleura	5.8
ED2	71	M	IV	Extensive	Bone	8.4
ED3	59	M	IV	Extensive	Bone, liver, pleura	10.8
ED4	64	F	IV	Extensive	Brain, adrenal gland	13.0
ED5	71	M	IV	Extensive	Bone	10.4
ED6	65	M	IV	Extensive	Lung, brain	12.6
ED7	69	M	IV	Extensive	Brain	9.2
ED8	65	M	IV	Extensive	Brain	24.4
ED9	73	M	IV	Extensive	Pleura	**1.4**
ED10	68	M	IV	Extensive	Brain, adrenal gland	7.4
ED11	77	F	IV	Extensive	Lung, bone	13.8
ED12	62	M	IV	Extensive	Brain	30.0
ED13	59	M	IV	Extensive	Brain	35.2
ED14	58	M	IV	Extensive	Pleura	**2.8**
ED15	62	F	IV	Extensive	Bone, liver	13.2
ED16	52	M	IV	Extensive	Bone, liver	9.6
ED17	77	F	IV	Extensive	Lung, bone	4.8
ED18	77	M	IIIC	Extensive	None	1.6
ED19	70	M	IIIC	Extensive	None	1.2
ED20	52	M	IIIB	Extensive	None	2.4
LD1	66	M	IIIA	Limited	None	2.6
LD2	51	M	IIIA	Limited	None	0.2
LD3	53	M	IIB	Limited	None	1.8
LD3	66	M	IIIA	Limited	None	0.0
LD5	60	F	IIIA	Limited	None	1.4
LD6	79	M	IIB	Limited	None	0.4

No	Age	Sex	CTC#/ml	Risk	No	Age	Sex	CTC#/ml	Risk
H1	43	M	0.0	current smoker	H11	47	M	0.0	current smoker
H2	77	M	0.0	current smoker	H12	56	M	0.0	current smoker
H3	75	F	0.0	current smoker	H13	58	M	0.0	benign nodule
H4	38	F	0.0	current smoker	H14	70	F	0.0	benign nodule
H5	63	M	0.0	current smoker	H15	61	M	0.0	benign nodule
H6	63	F	0.0	current smoker	H16	67	F	0.0	benign nodule
H7	36	M	0.0	current smoker	H17	55	F	0.0	benign nodule
H8	52	M	0.0	current smoker	H18	39	M	0.0	benign nodule

H9	58	M	0.0	current smoker	H19	74	M	0.6	benign nodule
H10	66	F	0.0	current smoker	H20	70	M	0.0	benign nodule

Supplementary Table 8. Clinicopathological characteristics, CTC counts and scMet-Seq results of SCLC patients in the validation cohort. The false negative results of scMet-Seq in diagnosing metastatic SCLC are shown in red.

No	Age	Sex	TNM Stage	ED/LD stage	Distant metastasis	CTC#/ml	CTC#>cut-off (3.0/ml)	scMet-Seq
LC1	65	M	IV	Extensive	Liver	6.6	Y	Positive
LC2	77	M	IV	Extensive	Bone, liver, pleura	16.0	Y	Positive
LC3	63	M	IV	Extensive	Lung	35.6	Y	Positive
LC4	63	M	IV	Extensive	Bone, liver, pleura	22.0	Y	Positive
LC5	73	M	IV	Extensive	Bone, adrenal gland	13.8	Y	Positive
LC6	61	M	IV	Extensive	Bone	7.4	Y	Positive
LC7	70	M	IIIC	Extensive	None	1.2	N	Negative
LC8	71	M	IV	Extensive	Pleaura	2.6	N	**Negative**
LC9	47	F	IV	Extensive	Bone, pleura	15.4	Y	Positive
LC10	61	M	IV	Extensive	Adrenal gland	10.8	Y	Positive
LC11	68	F	IV	Extensive	Bone	6.4	Y	Positive
LC12	66	M	IIB	Limited	None	0.4	N	Negative
LC13	75	F	IIIA	Limited	None	1.2	N	Negative
LC14	70	M	IIIA	Limited	None	2.2	N	Negative
LC15	54	F	IIIA	Limited	None	0.8	N	Negative
