# Supplementary material for: Single-cell low-pass whole genome sequencing accurately detects circulating tumor cells for liquid biopsy-based multi-cancer diagnosis
Source: NPJ Precis Oncol. 2024 Feb 6;8:30. doi: 10.1038/s41698-024-00520-1 (PMC10847465; doi:10.1038/s41698-024-00520-1)
Supplement: Supplementary file 2 — Reporting summary [file 41698_2024_520_MOESM2_ESM.pdf]

nature portfolio

Corresponding author(s): Qihui Shi, Haimiao Xu, Ziming Li

Last updated by author(s): Dec 5, 2023

Reporting Summary

Nature Portfolio wishes to improve the reproducibility of the work that we publish. This form provides structure for consistency and transparency in reporting. For further information on Nature Portfolio policies, see our Editorial Policies and the Editorial Policy Checklist.

Statistics

For all statistical analyses, confirm that the following items are present in the figure legend, table legend, main text, or Methods section.

n/a	Confirmed	
☐	☒	The exact sample size (*n*) for each experimental group/condition, given as a discrete number and unit of measurement
☐	☒	A statement on whether measurements were taken from distinct samples or whether the same sample was measured repeatedly
☐	☒	The statistical test(s) used AND whether they are one- or two-sided<br>*Only common tests should be described solely by name; describe more complex techniques in the Methods section.*
☒	☐	A description of all covariates tested
☐	☒	A description of any assumptions or corrections, such as tests of normality and adjustment for multiple comparisons
☐	☒	A full description of the statistical parameters including central tendency (e.g. means) or other basic estimates (e.g. regression coefficient) AND variation (e.g. standard deviation) or associated estimates of uncertainty (e.g. confidence intervals)
☐	☒	For null hypothesis testing, the test statistic (e.g. *F*, *t*, *r*) with confidence intervals, effect sizes, degrees of freedom and *P* value noted<br>*Give P values as exact values whenever suitable.*
☒	☐	For Bayesian analysis, information on the choice of priors and Markov chain Monte Carlo settings
☒	☐	For hierarchical and complex designs, identification of the appropriate level for tests and full reporting of outcomes
☐	☒	Estimates of effect sizes (e.g. Cohen's *d*, Pearson's *r*), indicating how they were calculated

Our web collection on statistics for biologists contains articles on many of the points above.

Software and code

Policy information about availability of computer code

Data collection	No software or codes were used to collect data.
Data analysis	Data analysis, statistical testing and visualization were conducted in Graphpad Prism (v.9.3.1), ImageJ (v1.52a), Microsoft Excel 2010, Origin8 Pro SR4. For genome sequencing data quality control, FastQC (v0.11.9) and Trimmomatic (v0.39) were used. For copy number alternation analysis, BWA (v0.7.17), Samtools (v1.11) and HMMcopy (v0.1.1) were used.

For manuscripts utilizing custom algorithms or software that are central to the research but not yet described in published literature, software must be made available to editors and reviewers. We strongly encourage code deposition in a community repository (e.g. GitHub). See the Nature Portfolio guidelines for submitting code & software for further information.

Data

Policy information about availability of data

All manuscripts must include a data availability statement. This statement should provide the following information, where applicable:

- Accession codes, unique identifiers, or web links for publicly available datasets
- A description of any restrictions on data availability
- For clinical datasets or third party data, please ensure that the statement adheres to our policy

Single-cell whole genome sequencing data and bulk DNA whole genome sequencing data of tumor tissues generated in this study have been deposited in the Genome Sequence Archive (GSA) under accession number HRA004452. Other data are available in the main text or the supplementary materials.

Research involving human participants, their data, or biological material

Policy information about studies with human participants or human data. See also policy information about sex, gender (identity/presentation), and sexual orientation and race, ethnicity and racism.

Reporting on sex and gender	Information of patient sex was provided in the Table of the main text and Supplementary Tables.
Reporting on race, ethnicity, or other socially relevant groupings	None of such information was collected in this study.
Population characteristics	Information of patient age, cancer diagnosis, treatment history and results of cytology was collected and provided in the Supplementary Tables.
Recruitment	For the ascites study, a total of 149 patients (≥18 years) with ascites were enrolled in this study with written informed consent, including 40 patients in the training cohort and 109 consecutive patients in the validation cohort. For the SCLC study, a total of 44 SCLC patients and 20 high-risk controls were enrolled in the study with written informed consent.
Ethics oversight	The ascites study was conducted at Zhejiang Cancer Hospital and approved by the institutional review board (#IRB2022145). The SCLC study was conducted at Shanghai Chest Hospital and approved by the institutional review board (#IS21109).

Note that full information on the approval of the study protocol must also be provided in the manuscript.

Field-specific reporting

Please select the one below that is the best fit for your research. If you are not sure, read the appropriate sections before making your selection.

☒ Life sciences ☐ Behavioural & social sciences ☐ Ecological, evolutionary & environmental sciences

For a reference copy of the document with all sections, see nature.com/documents/nr-reporting-summary-flat.pdf

Life sciences study design

All studies must disclose on these points even when the disclosure is negative.

Sample size	Due to the exploratory nature of the study, it is not appropriate to pre-specify the sample size. The sample size of the ascites cohort was based on the number of ascites samples available from Zhejiang Cancer Hospital and our previous experience on assessing diagnostic performance of biomarker-based tests. Likewise, the sample size of small-cell lung cancer (SCLC) cohort was based the number of SCLC patients available from Shanghai Chest Hospital and our precious experience with CTC detection.
Data exclusions	Six ascites samples and three blood samples were excluded from the analyses due to unobtainable diagnosis or sequencing results.
Replication	Bioinformatic analysis of copy number alternations were performed in eight different cancer types (ovarian, pancreatic, gastic, esophagela, colon, breast, LUAD and LUSC). Comparison of single-cell sequencing between the Tn5-based protocol and MALBAC protocol was performed in two different cell lines (H1650, H1975) and detected tumor cells from a patient sample (P8). Assaying HK2 intensity and HK2-CK combination intensity was performing in four different cell lines (H1650, H1975, HCC827 and H2228). For single-cell genome sequencing of clinical samples, our method requires sequencing of multiple cells and at least two cells exhibiting consistent copy number alternation profiles are considered as positive result. Thus, single-cell genome sequencing of clinical samples had multiple replicates. .
Randomization	Randomization is not applicable in this study.
Blinding	The clinical diagnosis of patients and results of cytological analysis on ascites were blinded to the operators who performed the immunostaining assay and single-cell genome sequencing. The investigators were not blinded during the training cohort study, but were blinded during the validation cohort study.

Reporting for specific materials, systems and methods

We require information from authors about some types of materials, experimental systems and methods used in many studies. Here, indicate whether each material, system or method listed is relevant to your study. If you are not sure if a list item applies to your research, read the appropriate section before selecting a response.

Materials & experimental systems

n/a	Involved in the study
☐	☒ Antibodies
☐	☒ Eukaryotic cell lines
☒	☐ Palaeontology and archaeology
☒	☐ Animals and other organisms
☒	☐ Clinical data
☒	☐ Dual use research of concern
☒	☐ Plants

Methods

n/a	Involved in the study
☒	☐ ChIP-seq
☒	☐ Flow cytometry
☒	☐ MRI-based neuroimaging

Antibodies

Antibodies used	Anti-HK2 primary antibody: Abcam (#ab209847);<br>Pan Cytokeratin Monoclonal Antibody (AE1/AE3) eFluor 570: Thermo Fisher Scientific (#41-9003-82);<br>Pan Cytokeratin Monoclonal Antibody (AE1/AE3), Alexa Fluor 488: Thermo Fisher Scientific (#53-9003-82);<br>Alexa Fluro 488-conjugated goat-anti-rabbit secondary antibody: Thermo Fisher Scientific (#A11008);<br>APC-conjugated anti-CD45: Thermo Fisher Scientific (#17-0459-42).
Validation	The antibodies were commercially available and validated by the manufacturer. APC-conjugated anti-CD45 was further validated with PBMC from human blood as a positive control and lung cancer cell lines as negative controls. Pan-CK antibodies and anti-HK2 were further validated with lung cancer cell lines as positive controls and PBMC from human blood as a negative control.

Eukaryotic cell lines

Policy information about cell lines and Sex and Gender in Research

Cell line source(s)	Lung cancer cell lines (H1650, H1975, HCC827, H2228, PC-9) used in this study were purchased from American Type Culture Collection (ATCC). Bladder cancer cell line RT4 was purchased from Cell Bank of the Chinese Academy of Sciences (Shanghai, China).
Authentication	Cell lines were authenticated by DNA short tandem repeat (STR) profiling analysis.
Mycoplasma contamination	Cell lines were tested negative for mycoplasma contamination.
Commonly misidentified lines<br>(See ICLAC register)	RT4 is listed in the database of commonly misidentified cell lines. RT4 used in this study was obtained from authentic stock and authenticated by DNA short tandem repeat (STR) profiling analysis. In the study, RT4 cells were used for validating the single-cell whole genome sequencing (WGS) protocols rather than biological function investigation. A variety of cell lines were used to validated the single-cell WGS protocols in this study.

Plants

Seed stocks	None of seed stocks or plant material were used in this study.
Novel plant genotypes	None
Authentication	None
